# Impact of RNA-seq data analysis algorithms on gene expression estimation and downstream prediction

**DOI:** 10.1038/s41598-020-74567-y

**Published:** 2020-10-21

**Authors:** Li Tong, Po-Yen Wu, John H. Phan, Hamid R. Hassazadeh, SEQC Consortium, Wendell D. Jones, Leming Shi, Matthias Fischer, Christopher E. Mason, Sheng Li, Joshua Xu, Wei Shi, Jian Wang, Jean Thierry-Mieg, Danielle Thierry-Mieg, Falk Hertwig, Frank Berthold, Barbara Hero, Yang Liao, Gordon K. Smyth, David Kreil, Paweł P. Łabaj, Dalila Megherbi, Gary Schroth, Hong Fang, Weida Tong, May D. Wang

**Affiliations:** 1 Department of Biomedical Engineering, Georgia Institute of Technology and Emory University, Atlanta, GA USA; 2 School of Electrical and Computer Engineering, Georgia Institute of Technology, Atlanta, GA USA; 3 School of Computational Science and Engineering, Georgia Institute of Technology, Atlanta, GA USA; 4 Genomic Laboratories, Q2 Solutions - EA Genomics, Morrisville, NC USA; 5 National Center for Toxicological Research, US Food and Drug Administration, Jefferson, AR USA; 6 School of Pharmacy and School of Life Sciences, Fudan University, Shanghai, China; 7 Department of Pediatric Oncology and Hematology and Center of Molecular Medicine Cologne, University of Cologne, Cologne, Germany; 8 Department of Physiology and Biophysics, Weill Cornell Medical College, New York, NY USA; 9 The HRH Prince Alwaleed Bin Talal Bin Abdulaziz Alsaud Institute for Computational Biomedicine, Weill Cornell Medical College, New York, NY USA; 10 Olivia Newton-John Cancer Research Institute, Heidelberg, VIC Australia; 11 School of Cancer Medicine, La Trobe University, Heidelberg, VIC Australia; 12 The Walter and Eliza Hall Institute of Medical Research, 1G Royal Parade, Parkville, VIC Australia; 13 School of Computing and Information Systems, The University of Melbourne, Parkville, VIC Australia; 14 Research Informatics, Lilly Corporate Center, Eli Lilly and Company, Indianapolis, IN USA; 15 National Center for Biotechnology Information, National Library of Medicine, National Institutes of Health, Bethesda, MD USA; 16 Bioinformatics Division, The Walter and Eliza Hall Institute of Medical Research, Parkville, VIC Australia; 17 Department of Computing and Information Systems, The University of Melbourne, Parkville, VIC Australia; 18 School of Mathematics and Statistics, The University of Melbourne, Parkville, VIC Australia; 19 Chair of Bioinformatics Research Group, Boku University Vienna, Vienna, Austria; 20 School of Life Sciences, University of Warwick, Coventry, UK; 21 Małopolska Centre of Biotechnology, Jagiellonian University, ul. Gronostajowa 7A, 30-387 Kraków, Poland; 22 Department of Electrical and Computer Engineering, CMINDS Research Center, University of Massachusetts, Lowell, MA USA; 23 Illumina Inc., Hayward, CA USA

**Keywords:** Gene expression analysis, Data processing, Predictive medicine, Next-generation sequencing, RNA sequencing

## Abstract

To use next-generation sequencing technology such as RNA-seq for medical and health applications, choosing proper analysis methods for biomarker identification remains a critical challenge for most users. The US Food and Drug Administration (FDA) has led the Sequencing Quality Control (SEQC) project to conduct a comprehensive investigation of 278 representative RNA-seq data analysis pipelines consisting of 13 sequence mapping, three quantification, and seven normalization methods. In this article, we focused on the impact of the joint effects of RNA-seq pipelines on gene expression estimation as well as the downstream prediction of disease outcomes. First, we developed and applied three metrics (i.e., accuracy, precision, and reliability) to quantitatively evaluate each pipeline’s performance on gene expression estimation. We then investigated the correlation between the proposed metrics and the downstream prediction performance using two real-world cancer datasets (i.e., SEQC neuroblastoma dataset and the NIH/NCI TCGA lung adenocarcinoma dataset). We found that RNA-seq pipeline components jointly and significantly impacted the accuracy of gene expression estimation, and its impact was extended to the downstream prediction of these cancer outcomes. Specifically, RNA-seq pipelines that produced more accurate, precise, and reliable gene expression estimation tended to perform better in the prediction of disease outcome. In the end, we provided scenarios as guidelines for users to use these three metrics to select sensible RNA-seq pipelines for the improved accuracy, precision, and reliability of gene expression estimation, which lead to the improved downstream gene expression-based prediction of disease outcome.

## Introduction

The first phase of the FDA-led microarray quality control project (MAQC-I) investigated the reliability of microarray platforms for gene expression estimation^1^. The second phase of the project, MAQC-II, studied 30,000 + microarray data analysis pipelines to assess the reproducibility of microarray-based predictive models^2^. Given the rise of the significance of next-generation sequencing in gene expression analysis, the FDA initiated the sequencing quality control project (SEQC) as a continuing MAQC effort to conduct an in-depth assessment of RNA-seq by combining the objectives of both MAQC-1 and MAQC-II^3–6^. Specifically, the goal of SEQC was to conduct a comprehensive evaluation of both RNA-seq technology and RNA-seq data analysis pipelines, which was similar to the objectives of MAQC-I and MAQC-II for microarrays. While Su et al. summarized the RNA-seq technology investigation^7^, this complementary article focuses on the RNA-seq data analysis pipelines targeting medical and health applications. Specifically, this article examines the effect of RNA-seq pipelines on gene expression with three critical metrics (i.e., accuracy, precision, and reliability), and further on downstream gene expression-based prediction of disease outcomes. Although other analyses of RNA-seq are possible (e.g., differential expression analysis^8–10^, alternative splicing^11–13^, and RNA fusion^14,15^), we focus on gene expression because it is the most widely used genetic variations in biomedical and health applications.

For medical and health applications, choosing a proper RNA-seq gene expression analysis pipeline remains a critical challenge due to its relative immaturity (i.e., fewer standards reported compared with microarrays), complexity, and diverse applicability^16,17^. We performed a literature survey on RNA-seq pipelines consisting of sequence mapping^18–29^, expression quantification^30–33^, and expression normalization^6,34–36^. For evaluation of RNA-seq pipelines, the majority strategy is to use some sorts of benchmark datasets, or reference standards to enable quality control (e.g., natural well-characterized genetic materials or synthetic spike-in controls)^37^. For benchmark dataset-based evaluation, multiple comparative investigations exist for focusing on individual components of RNA-seq pipelines, such as mapping alone^25,38–42^, quantification only^33,43^, or normalization^34,44,45^. Correspondingly, the joint impact of each of these three components is less understood. For examples, a previous analysis of 50 RNA-seq pipelines examined combinations of ten mapping and five quantification algorithms, but it did not study the effect of different normalization methods’ impact and the interaction effect among pipeline components^46^; another study investigated three mapping, two quantification, and five differentially expressed gene (DEG) detection methods, but it did not report the interaction effect among components either^47^; a third study by Sahraeian et al. examined 120 combinations of 39 tools^14^ for RNA variant calling, RNA editing, RNA fusion, gene expression, and differential expression, but it did not provide an assessment on their impact on gene-expression-based downstream prediction. Most FDA approvals on medical genomics would be relevant to gene expression applications. To the best of our knowledge, no studies have comprehensively examined the joint effect of RNA-seq pipeline components on gene expression and its downstream prediction of disease outcomes. Thus, this article dedicates to this goal.

**Figure 1:**
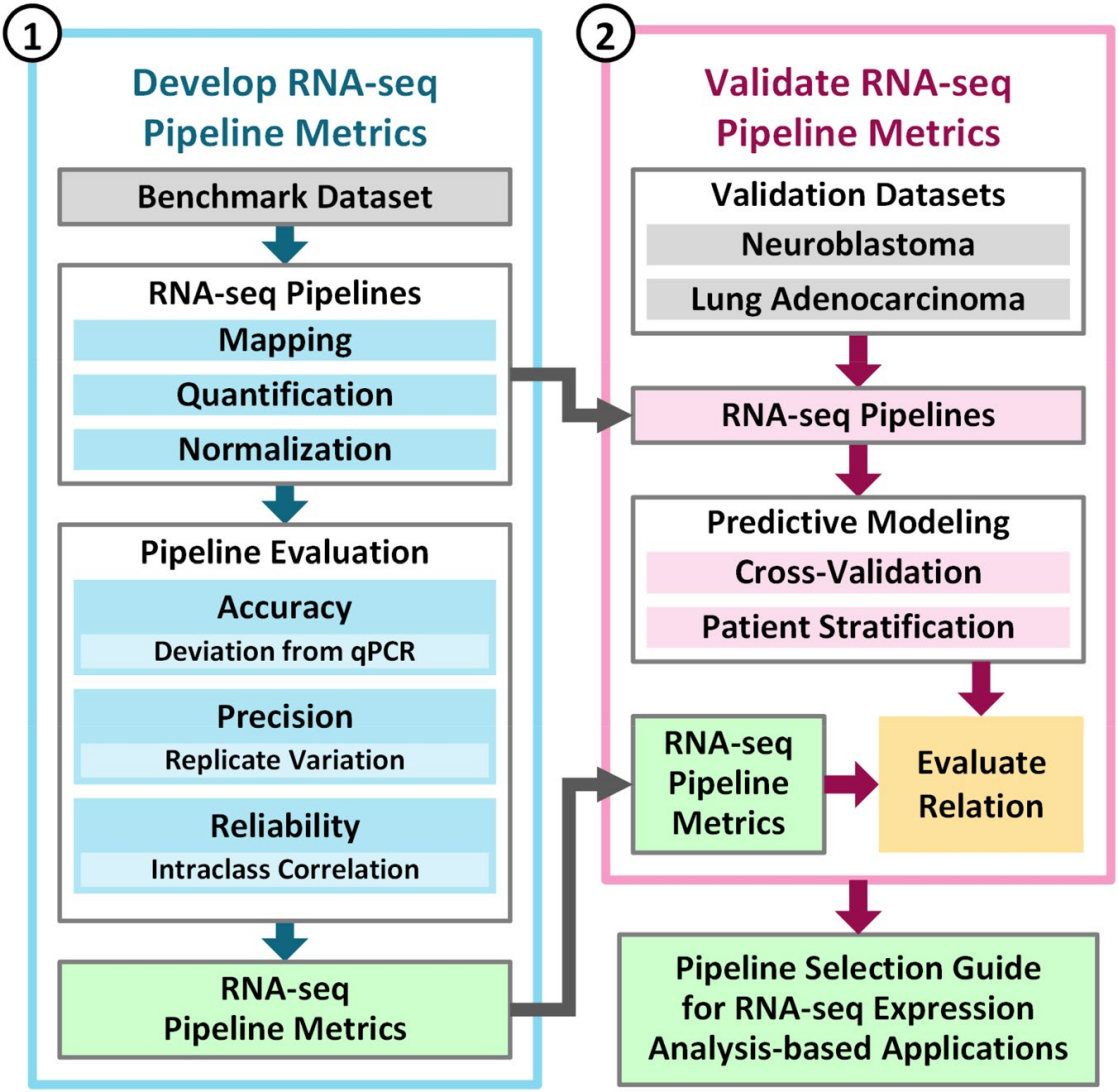
The SEQC consortium developed and validated a guideline for selecting RNA-seq pipelines for gene expression-based predictive modeling using the SEQC-benchmark, SEQC-neuroblastoma, and TCGA-lung-adenocarcinoma datasets. Phase-1 of the investigation developed the metrics that captured the accuracy, precision, and reliability of RNA-seq pipelines (the blue box). Using the SEQC-neuroblastoma and TCGA-lung-adenocarcinoma datasets, Phase-2 of the investigation determined that RNA-seq pipeline metrics can be used to select pipelines that result in better performance in terms of predicting cancer outcome (the pink box).

The FDA coordinated multiple sites of SEQC to generate a multi-replicate benchmark dataset (referred to as SEQC-benchmark)^7^ and a clinical dataset consisting of neuroblastoma patient samples (referred to as SEQC-neuroblastoma)^48^. In addition, we have another real-world clinical dataset on lung adenocarcinoma (referred to as TCGA-lung-adenocarcinoma) from The Cancer Genome Atlas (TCGA). These datasets were used to investigate the joint impact of pipeline components on downstream gene expression-based prediction in a two-phase study:

1. *Phase-1* we developed three metrics—accuracy, precision, and reliability—for assessing the performance of a representative set of 278 RNA-seq pipelines (Fig. 1, blue box) using the SEQC-benchmark dataset (i.e., A, B, C and D where C is 75/25 of A/B while D is 25/75 of A/B).
2. *Phase-2* we validated the benchmark metrics by quantifying gene expression in the SEQC-neuroblastoma dataset and the TCGA-lung-adenocarcinoma dataset, and demonstrated that the benchmark metrics are informative for inferring downstream prediction of disease outcome (Fig. 1, pink box).

Our comprehensive investigation revealed that RNA-seq pipeline components—mapping, quantification, and normalization—jointly impacted the accuracy, precision, and reliability of gene expression, and consequently, affected the downstream performance of predicting neuroblastoma and lung adenocarcinoma outcome. RNA-seq pipelines that performed well in gene expression estimation also performed well in downstream prediction of disease outcome.

## Results

### Phase-1: assessing the joint impact of pipeline components on gene expression estimation with the benchmark metrics

We systematically investigated 278 RNA-seq pipelines (Supplementary Table S1) that included combinations of mapping, quantification, and normalization components listed in Supplementary Tables S2–S4. Sequence mapping algorithms were further categorized based on mapping strategy (i.e., un-spliced and spliced) and mapping reporting (i.e., single-hit and multi-hit) (refer to the “Method” section “Sequence mapping”). To gain insight into these pipelines, we used the SEQC-benchmark dataset and a quantitative PCR (qPCR) benchmark dataset. Because the qPCR results vary among platforms^7^, we filtered them to keep the genes that fit the titration ratio of A–D samples (Supplementary Fig. S1). After filtering with the titration ratio, we only used 10,222 genes (out of a total of 20,801 genes assayed with qPCR) as a benchmark reference. Supplementary Tables S5 and S6 summarize SEQC benchmark samples and datasets. Supplementary Table S7 summarizes the three metrics—accuracy, precision, and reliability—used to evaluate each pipeline. These metrics are detailed in the “Method” section. The joint effect of mapping, quantification, and normalization with respect to these three metrics was assessed comprehensively for all genes (the entire set of 10,222 genes, referred as AllGenes hereafter) and for the low-expression genes (2044, the subset of AllGenes, referred as LowExpressGenes hereafter) as described in Supplementary Fig. S1 and “Method” section.

**Figure 2:**
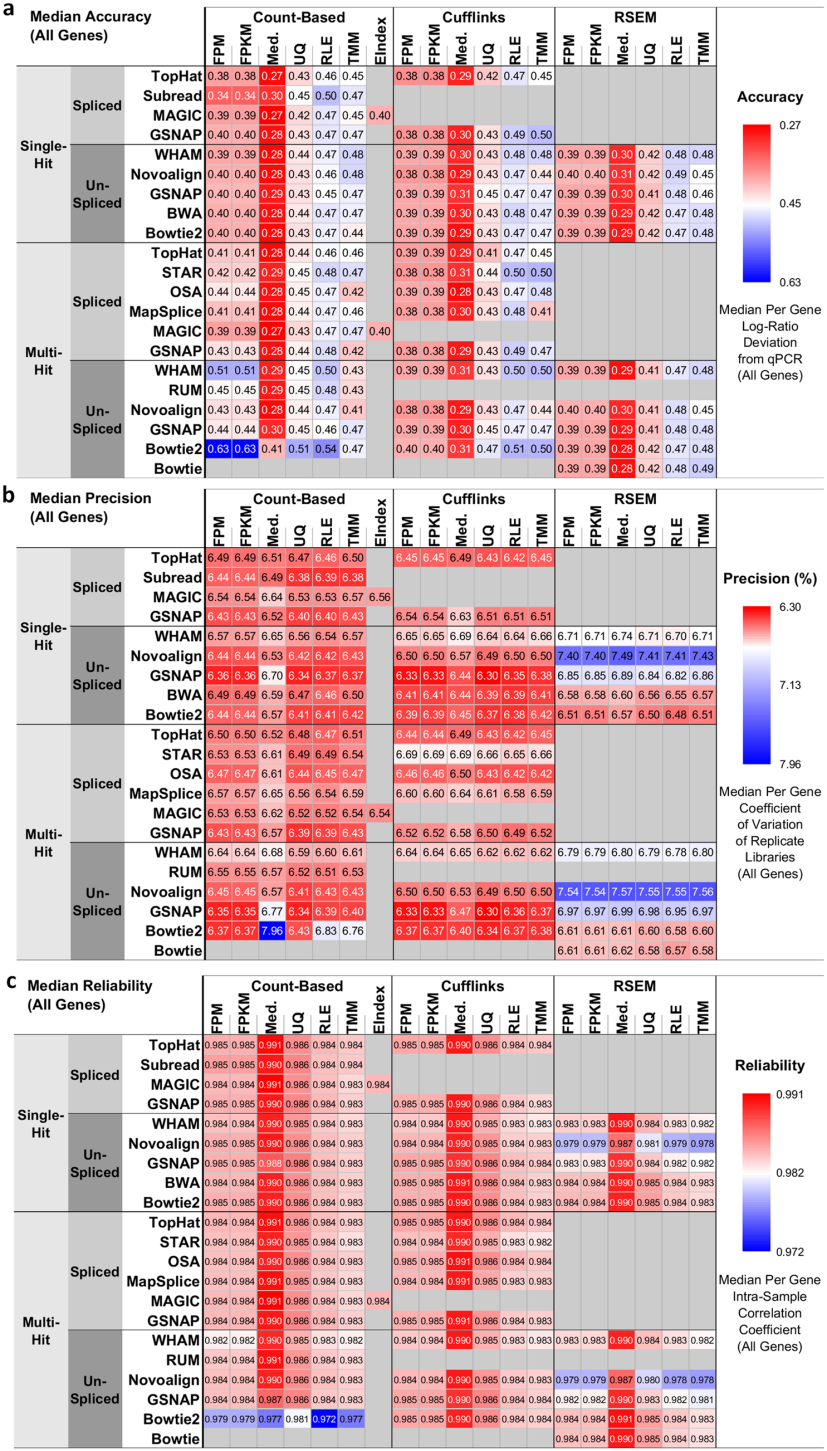
The 278 RNA-seq pipelines applied to the SEQC-benchmark dataset differ in terms of gene expression accuracy, precision, and reliability. In each heatmap, the rows are different settings for 13 aligners and the columns are combinations of three quantification and seven normalization methods. (**a**) Accuracy is defined as the deviation of pipeline-derived log ratios of gene expression from the corresponding qPCR-based log ratios. Median accuracy of all genes (i.e., 10,222 genes) is encoded as color, with red representing the highest accuracy, or the lowest deviation from qPCR. (**b**) Precision is defined as the coefficient of variation (CoV) of gene expression over replicate libraries. Median precision of all genes is encoded as color, with red indicating the highest precision, or the lowest CoV. (**c**) Reliability is defined as the intraclass (or intra-sample in our context) correlation that quantifies how similar replicate libraries of a sample are to one another using analysis of variance techniques. Median reliability of all genes is encoded as color, with red representing the highest reliability, or the highest intraclass correlation. Refer to the “Method” section for mathematical definitions of accuracy, precision, and reliability in the context of RNA-seq pipelines.

**Figure 3:**
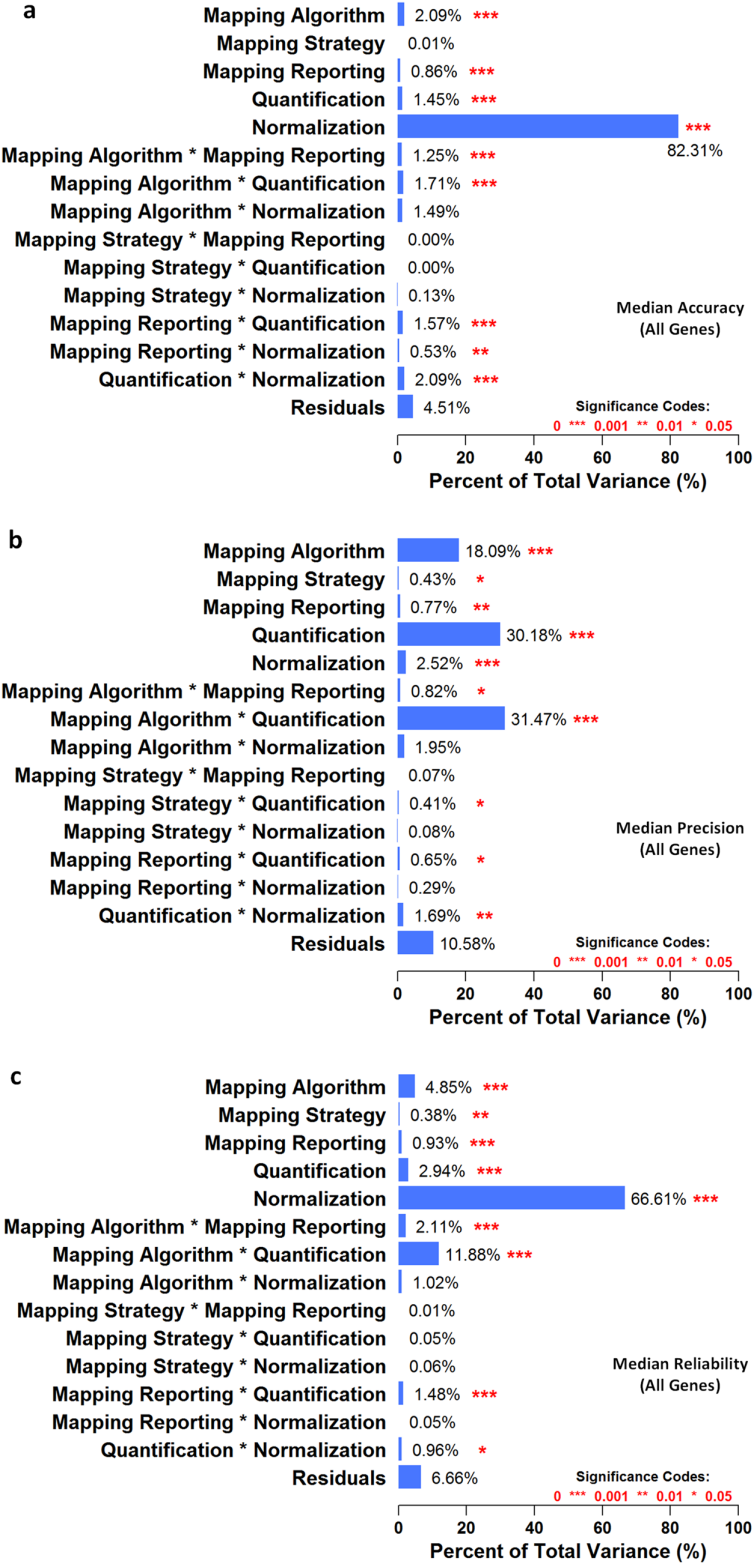
Analysis of variance decomposes the overall variance in (**a**) median accuracy of all genes, (**b**) median precision of all genes, and (**c**) median reliability of all genes into various factors considered, including five RNA-seq pipeline components (i.e., mapping algorithm, mapping strategy, mapping reporting, quantification, and normalization) and nine associated two-way interactions. The statistical significance of each component’s or interaction’s contribution is denoted by red asterisks, with ‘***’ indicating p-values are smaller than 0.001, ‘**’ indicating p-values are smaller than 0.01, and ‘*’ indicating p-values are smaller than 0.05.

We defined the *accuracy* metric as the deviation of RNA-seq pipeline-derived log ratios of gene expression from the corresponding qPCR-based log ratios and visualized the median accuracy of AllGenes and LowExpressGenes using heatmaps (Fig. 2a, Supplementary Fig. S2). We observed the following results:

1. Using AllGenes, the log-ratio deviation between RNA-seq and qPCR ranged from 0.27 to 0.63 (Fig. 2a). A smaller deviation represents higher accuracy. Median normalization exhibited the lowest deviation, or the highest accuracy, compared with all other normalization methods. In addition, for all mapping-quantification combinations, the [Bowtie2 multi-hit + count-based] pipelines showed the largest deviation. Moreover, pipelines with multi-hit mapping and count-based quantification generally showed a larger deviation than other pipelines. Among all pipeline factors, normalization was the largest statistically significant (p < 0.05) source of variation (Fig. 3a).
2. The log-ratio deviation using LowExpressGenes was larger than that using AllGenes, and it ranged from 0.45 to 0.69 (Supplementary Fig. S2). The trends of pipeline performance were similar to those using AllGenes, and normalization was also the largest statistically significant (p < 0.05) source of variation (Supplementary Fig. S3).
3. In summary, median normalization with most mapping and quantification algorithms, except for the [Bowtie2 multi-hit + count-based] pipelines, was the best choice for quantifying genes with high accuracy, or low deviation from qPCR.

We defined the *precision* metric as the coefficient of variation (CoV) of gene expression across replicate libraries, and visualized the median precision of AllGenes and LowExpressGenes using heatmaps (Fig. 2b, Supplementary Fig. S4). We observed the following results:

1. Using AllGenes, the CoV ranged from 6.30 to 7.96% (Fig. 2b). Smaller CoV represents higher precision. Pipelines with any of Novoalign, GSNAP un-spliced, or WHAM mapping, and RSEM quantification resulted in higher CoV, or lower precision, despite the choice of normalization methods. In addition, the [Bowtie2 multi-hit + count-based + med.] pipeline led to the largest CoV. Moreover, for each mapping-normalization combination, pipelines with either count-based or Cufflinks quantification reported higher precision than those with RSEM quantification, except the [Bowtie2 multi-hit + count-based + med.] pipeline mentioned previously. Quantification, mapping algorithm, and their interaction were the largest statistically significant (p < 0.05) sources of variation (Fig. 3b).
2. The CoV using LowExpressGenes was larger than that using AllGenes, and it ranged from 11.0 to 15.6% (Supplementary Fig. S4). The trends of pipeline performance were similar to those using AllGenes, except that the [Bowtie2 multi-hit + count-based] pipelines exhibited the highest precision among others. Again, quantification, mapping algorithm, and their interaction were the largest statistically significant (p < 0.05) source of variation (Supplementary Fig. S5).
3. In summary, pipelines with any of Bowtie2 multi-hit, GSNAP un-spliced, or Subread mapping and either count-based or Cufflinks quantification, except for the [Bowtie2 multi-hit + count-based + med.] pipeline, were the best choice for quantifying genes with high precision, or low CoV.

We defined the *reliability* metric as the intra-class (i.e., intra-sample in the context of the SEQC-benchmark dataset) correlation (ICC) of gene expression, and visualized the median reliability of AllGenes and lowExpressGenes using heatmaps (Fig. 2c, Supplementary Fig. S6). We observed the following results:

1. Using AllGenes, the ICC ranged from 0.972 to 0.991 (Fig. 2c). Larger ICC represents higher reliability. Median normalization exhibited the highest ICC, or the highest reliability, compared with all other normalization methods. In addition, pipelines with Novoalign mapping and RSEM quantification resulted in lower ICC for all but Median normalization. Moreover, the [Bowtie2 multi-hit + count-based] pipelines showed the lowest ICC. Furthermore, for each mapping-normalization combination, pipelines with either count-based or Cufflinks quantification always reported higher ICC than those with RSEM quantification, except the [Bowtie2 multi-hit + count-based] pipelines mentioned previously. Normalization was the largest statistically significant (p < 0.05) source of variation (Fig. 3c), followed by two-way [mapping algorithm*quantification] interaction.
2. The ICC using LowExpressGenes was smaller than that using AllGenes, and it ranged from 0.938 to 0.975 (Supplementary Fig. S6). The trends of pipeline performance were similar to those using AllGenes, except that the [Novoalign + RSEM] pipelines exhibited the lowest ICC, followed by the [Bowtie2 multi-hit + count-based] pipelines. Normalization, two-way [mapping algorithm*quantification] interaction, quantification, and mapping algorithm were the largest statistically significant (p < 0.05) sources of variation (Supplementary Fig. S7).
3. In summary, median normalization along with most mapping and quantification algorithms, except for the [Bowtie2 multi-hit + count-based] and [Novoalign + RSEM] pipelines, was the best choice for quantifying genes with high reliability, or high ICC.

We also examined whether the performance of metrics depended on the characteristics of sequence mapping results. We used M-estimation with Huber weighting to fit robust linear models that capture the relationship between the benchmark metrics and alignment profiles (see the “Method” section for details). The accuracy metric correlated with the number of mismatches per mapped read, and the precision and reliability metrics correlated with the number of mapped fragments (Supplementary Fig. S12). Fewer mismatches per read and more mapped fragments tended to lead to more accurate, precise, and reliable gene expression.

In summary, the *Phase-1* investigation using the SEQC-benchmark dataset demonstrated that gene expression estimation is significantly impacted by the joint effect of multiple RNA-seq pipeline components (Figs. 2, 3).

### Phase-2: the impact of RNA-seq pipeline on the disease outcome prediction performance

We used the SEQC-neuroblastoma and TCGA-lung-adenocarcinoma datasets to assess the impact of upstream RNA-seq pipeline components on the downstream prediction of disease outcome using gene expression (Fig. 1). The SEQC-neuroblastoma dataset, provided by the SEQC consortium, contains RNA-seq data of 176 primary neuroblastomas obtained from high-risk patients with well-annotated clinical data^48^, in which survival information, including event-free survival (EFS) and overall survival (OS), was used for defining group labels (Supplementary Table S9). The TCGA-lung-adenocarcinoma dataset contains RNA-seq data of patients with known survival time used for defining group labels (Supplementary Table S10).

We used the same set of 278 RNA-seq pipelines to process the SEQC-neuroblastoma dataset (we used only 156 out of the 278 pipelines for the TCGA-lung-adenocarcinoma dataset). For each set of estimated gene expression (278 for neuroblastoma and 156 for lung adenocarcinoma), we performed nested cross-validation (Supplementary Fig. S13, “Method” section) using three classifiers—adaptive boosting, logistic regression, and support vector machines, which are proven to be robust and mostly used in machine learning. For each clinical endpoint—neuroblastoma EFS, neuroblastoma OS, and lung adenocarcinoma survival—we calculated the AUC (area under the ROC curve) and MCC (Matthews correlation coefficient), and visualized these using heatmaps (Supplementary Figs. S14–S16). We observed the following results:

1. For the neuroblastoma EFS endpoint, pipelines using count-based quantification with TMM, RLE, upper quartile, or median normalization tended to achieve high AUC and MCC; while those with FPM or FPKM normalization tended to perform poorly. In addition, Novoalign with Cufflinks and Bowtie2 or BWA with RSEM led to poor AUC and MCC, especially when combining with FPM or FPKM normalization (Supplementary Fig. S14).
2. For the neuroblastoma OS endpoint, median normalization led to higher AUC and MCC than other normalization methods for most mapping-quantification combinations. GSNAP un-spliced mapping performed well with count-based or Cufflinks quantification but not RSEM quantification. In addition, pipelines with RSEM quantification and any of upper quartile, RLE, or TMM normalization tended to result in poor AUC and MCC (Supplementary Fig. S15).
3. For the lung adenocarcinoma survival endpoint, pipelines with count-based quantification and TMM normalization tended to achieve high AUC and MCC. TopHat alignment with either count-based or Cufflinks quantification also performed well. In contrast, pipelines with any of Novoalign single-hit, STAR, GSNAP un-spliced multi-hit, or Bowtie2 multi-hit and Cufflinks resulted in lower AUC and MCC (Supplementary Fig. S16).
4. ANOVA for each neuroblastoma endpoint showed that normalization was the largest statistically significant (p < 0.05) source of variation, followed by mapping algorithm, two-way [mapping algorithm*quantification] interaction, and two-way [quantification*normalization] interaction (Supplementary Figs. S17, S18). For the lung adenocarcinoma endpoint, several pipeline components and their interactions contributed more evenly to the overall variance that may be due to only 156 pipelines were conducted (Supplementary Fig. S19). All ANOVA reported large residual variance that should be explained by higher order interactions.

These results suggested that the choice of upstream RNA-seq pipeline components significantly impacted the performance of downstream prediction of disease outcome. Supplementary Tables S11 and S12 summarize the predictive modeling performance for the 278 and 156 RNA-seq pipelines applied to the SEQC-neuroblastoma and TCGA-lung-adenocarcinoma datasets, respectively.

**Figure 4:**
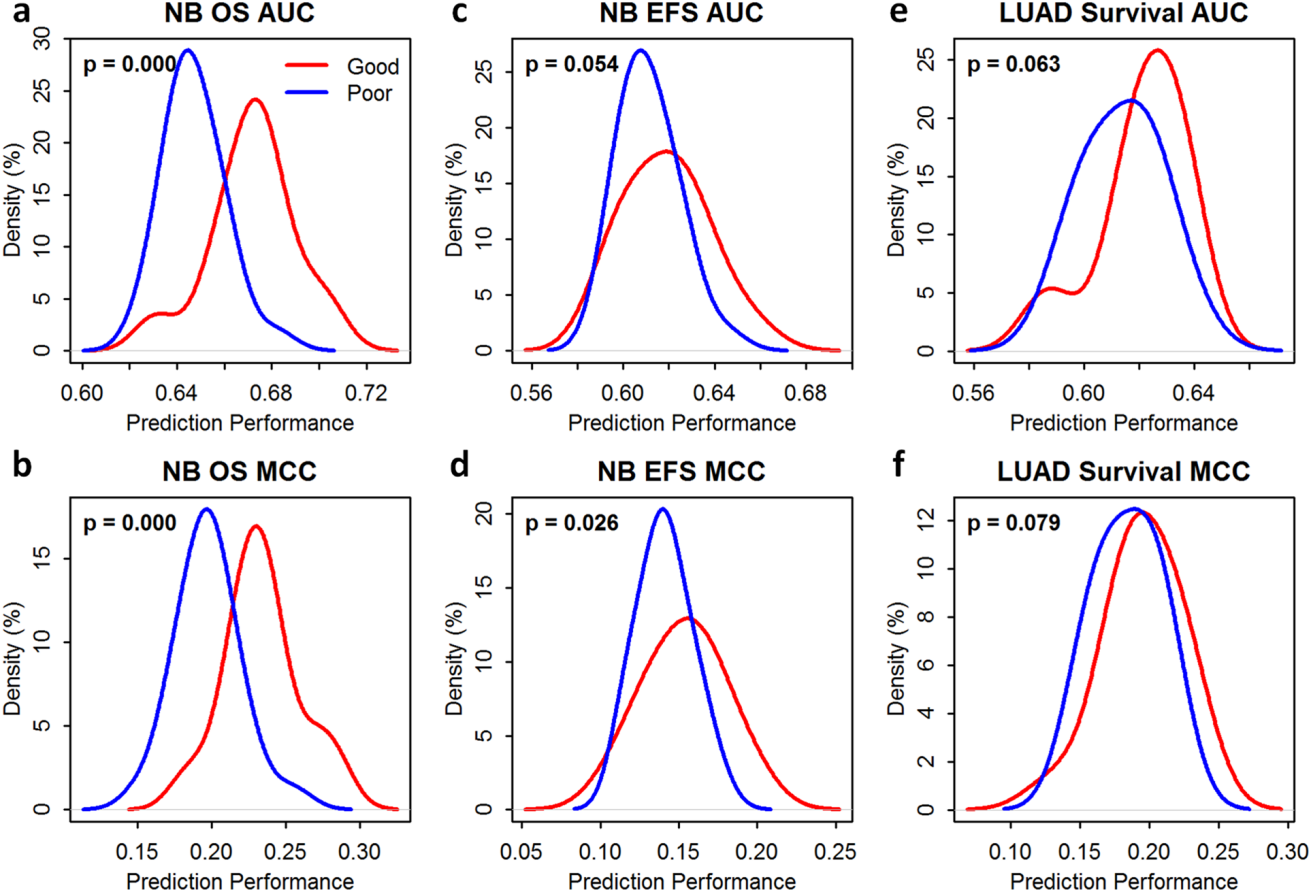
RNA-seq pipelines selected based on benchmark metrics (i.e., accuracy, precision, and reliability) were informative for inferring the performance of gene-expression-based prediction of disease outcome—(**a**) prediction performance measured by the area under the receiver operating characteristic curve (AUROC, or AUC) for the overall survival (OS) endpoint of the SEQC-neuroblastoma (NB) dataset; (**b**) prediction performance measured by the Matthews correlation coefficient (MCC) for the OS endpoint of the SEQC-NB dataset; (**c**) prediction performance measured by the AUC for the event-free survival (EFS) endpoint of the SEQC-NB dataset; (**d**) prediction performance measured by the MCC for the EFS endpoint of the SEQC-NB dataset; (**e**) prediction performance measured by the AUC for the survival endpoint of the TCGA-lung-adenocarcinoma (LUAD) dataset; and (**f**) prediction performance measured by the MCC for the survival endpoint of the TCGA-LUAD dataset. The red line in each panel shows the probability density of the prediction performance of good-performing RNA-seq pipelines selected based on benchmark metrics; and the blue line demonstrates that of poor-performing pipelines selected based on the same. Statistical significance (i.e., p-values) was determined using the one-sided Wilcoxon rank-sum test. (**a**,**b**,**d**) show statistically significant difference (*p* < 0.05) between the two groups (i.e., the prediction performance of good-performing pipelines vs. that of poor-performing pipelines). The good-performing (Top 10%) and poor-performing pipelines (Bottom 10%) were determined based on the average rank of each RNA-seq pipeline over all benchmark metrics of both all and low-expressing genes.

We ranked the 278 RNA-seq pipelines base on the average rank of a combination of the three metrics. The top 10% pipelines were chosen as the good-performing pipelines while the bottom 10% pipelines were chosen as the poor-performing pipelines. We then compared good-performing versus poor-performing pipelines in conducting gene-expression-based prediction of disease outcome and the success rates of patient stratification for the three endpoints (see the “Method” section for details). The comparison was assessed with the one-sided Wilcoxon rank-sum test (Fig. 4).

For the prediction of neuroblastoma OS endpoint, average prediction performance (i.e., AUC and MCC) of good-performing pipelines was statistically significantly (p < 0.05) larger than that of poor-performing pipelines (Fig. 4a,b). For the prediction of neuroblastoma EFS endpoint, average MCC of good-performing pipelines was statistically significantly (p < 0.05) larger than that of poor-performing pipelines (Fig. 4c) and the average AUC of good-performing pipelines was larger than that of poor-performing pipelines with p slightly larger than 0.05 (Fig. 4d). For the prediction of LUAD survival endpoint, average prediction performance (i.e., AUC and MCC) of good-performing pipelines was larger than that of poor-performing pipelines (Fig. 4e,d) but also with p slightly larger than 0.05.

**Figure 5:**
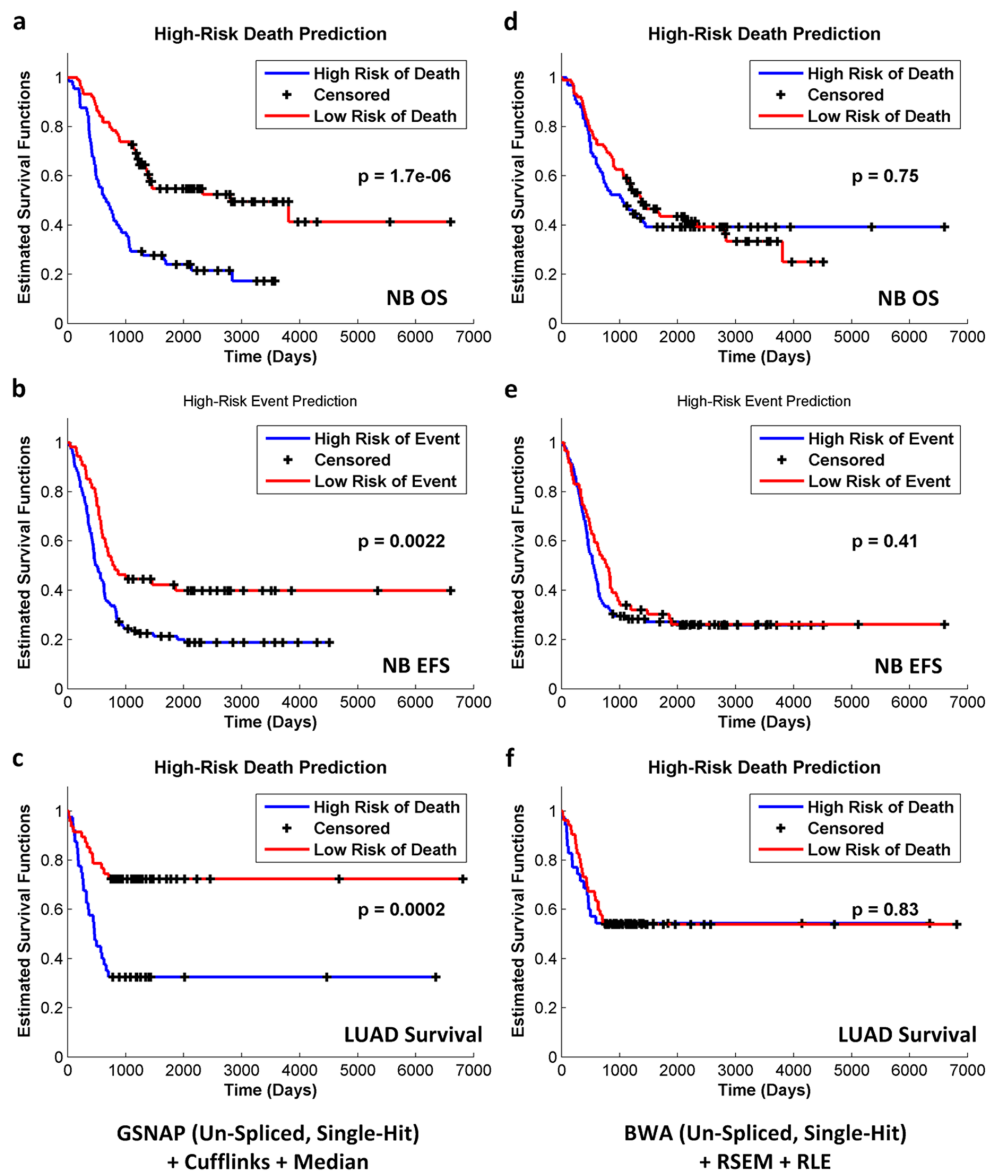
The RNA-seq pipeline selection guide was validated by assessing the ability of pipelines to stratify patients based on Kaplan–Meier survival analysis. For each pipeline, patients were grouped by predictive labels (i.e., high risk vs. low risk), and two Kaplan–Meier curves were plotted. The two-tailed log-rank test was used to determine the statistical significance of the seperation between the two curves. For good-performing pipelines selected based on benchmark metrics, the success rates of patient stratification (i.e., predictive labels led to a statistically significant separation of Kaplan–Meier curves) were higher. For example, the success rates of the [GSNAP (un-spliced, single-hit) + Cufflinks + Median] pipeline were 93%, 70%, and 67% for the SEQC-NB OS, SEQC-NB-EFS, and TCGA-LUAD-Survival endpoints, respectively (Supplementary Table S13). (**a**–**c**) demonstrate the most statistically significant separation of the two Kaplan–Meier curves for each endpoint. In contrast, poor-performing pipelines led to lower success rates of patient stratification. For instance, the success rates of the [BWA (un-spliced, single-hit) + RSEM + RLE] pipeline were 33%, 30%, and 33% for the SEQC-NB OS, SEQC-NB-EFS, and TCGA-LUAD-Survival endpoints, respectively (Supplementary Table S13). (**d**–**f**) demonstrate the least statistically significant separation of the two Kaplan–Meier curves for each endpoint.

In addition, good-performing pipelines (e.g., the [GSNAP un-spliced single-hit + Cufflinks + median] pipeline) tended to result in higher success rates of patient stratification than poor-performing pipelines (e.g., the [BWA + RSEM + RLE] pipeline). Figure 5 demonstrates Kaplan–Meier estimated survival functions for high-risk and low-risk patients for all endpoints. Good-performing pipelines tended to achieve statistically significant separation (p < 0.05) of the two patient groups (Fig. 5a–c), while poor-performing pipelines were more likely to fail (Fig. 5d–f). Supplementary Table S13 summarizes the success rates of patient stratification (i.e., p < 0.05 based on the two-tailed log-rank test) for the 278 and 156 RNA-seq pipelines applied to the SEQC-neuroblastoma and TCGA-lung-adenocarcinoma datasets, respectively.

## Discussion

We performed a systematic investigation of the 278 representative RNA-seq pipelines in two sequential phases. In Phase-1**,** we developed three metrics to characterize RNA-seq pipelines using the SEQC-benchmark dataset: (1) *accuracy* measures gene expression estimation against the qPCR ground truth results, providing justification to support downstream biological interpretation; (2) *precision* assesses the fluctuation of such a measurement across replicates, estimating the measurement behavior; (3) *reliability* the consistency of such a measurement among all the samples, offering the confidence of such a measurement. All these metrics are of great value to support the downstream biological interpretation of gene expression results. We observed that RNA-seq pipeline components jointly affected gene expression estimation (Figs. 2, 3). This comprehensive investigation provides a framework to assist pipeline selection, which had not previously been reported where individual RNA-seq pipeline components are usually focused (e.g., mapping^42^).

Supplementary Table S15 summarizes and compares the results of our study to previous studies focusing on individual pipeline components. For example, previous studies observed that RUM, GSNAP spliced, STAR, and MapSplice mapping led to more accurate base-level alignment and splice junction detection^25,39^. In addition, BWA, Bowtie, and Bowtie2 mapping were reported to be robust to sequencing errors and indels^38^. We similarly observed considerable differences in alignment profiles among mapping algorithms, and such the differences led to variations in the benchmark metrics (Supplementary Fig. S12, “Method” section). For example, Bowtie2 multi-hit mapping aligned many more reads, a higher percentage of which were sub-optimal mapping variants (i.e., secondary mappings, mismatches, insertions, deletions, and splicing), than WHAM single-hit mapping (Supplementary Fig. S20a–c). Consequently, pipelines with Bowtie2 multi-hit mapping resulted in a larger deviation from the qPCR reference, or lower accuracy, than those with WHAM single-hit mapping. However, such the observation applied to only count-based quantification but not Cufflinks or RSEM (Fig. 2a, Supplementary Fig. S2). In addition to the observations corresponding to previous literature, we also observed a joint effect between mapping and quantification components.

Variations in mapping performance propagated to the quantification stage. The quantification strategy for multi-hit mappers may explain the variation in gene expression accuracy. For example, Cufflinks and RSEM use Poisson distribution-based models and assign probabilities to each mapping while HTSeq simply counts total mapped reads regardless of quality. Thus, Cufflinks and RSEM are better able to handle multi-hit information, resulting in a smaller deviation from the qPCR reference (Fig. 2a, Supplementary Figs. S2, S20d). It is worthwhile to point out that, although variation in performance was observed for different pipelines investigated, singling out one pipeline for general application is not justified. This is specifically true by giving the fact that the joint effect of multiple components plays a role. Therefore, we aim to demonstrate the importance of pipelines selection and their impact on downstream analysis, such as classification.

In Phase-2, using the SEQC-neuroblastoma and TCGA-lung-adenocarcinoma datasets, we showed that RNA-seq pipelines with better performance in gene expression estimation (using average ranks of three metrics) resulting in a better downstream prediction of disease outcome. Subsequently, we demonstrated the effect of pipeline selection on patient stratification and provided a guideline for pipeline selection. Compared to previous RNA-seq pipeline evaluation studies that were restricting on genetic variations (e.g., gene fusions)^14^, we have extended the scope to downstream applications such as survival predictions using gene expression. The results revealed that RNA-seq pipelines jointly impact the gene expression estimation, and the influence will carry on to downstream applications. Most importantly, we found that RNA-seq pipelines that produced more accurate gene expression resulted in better survival prediction performance.

**Figure 6:**
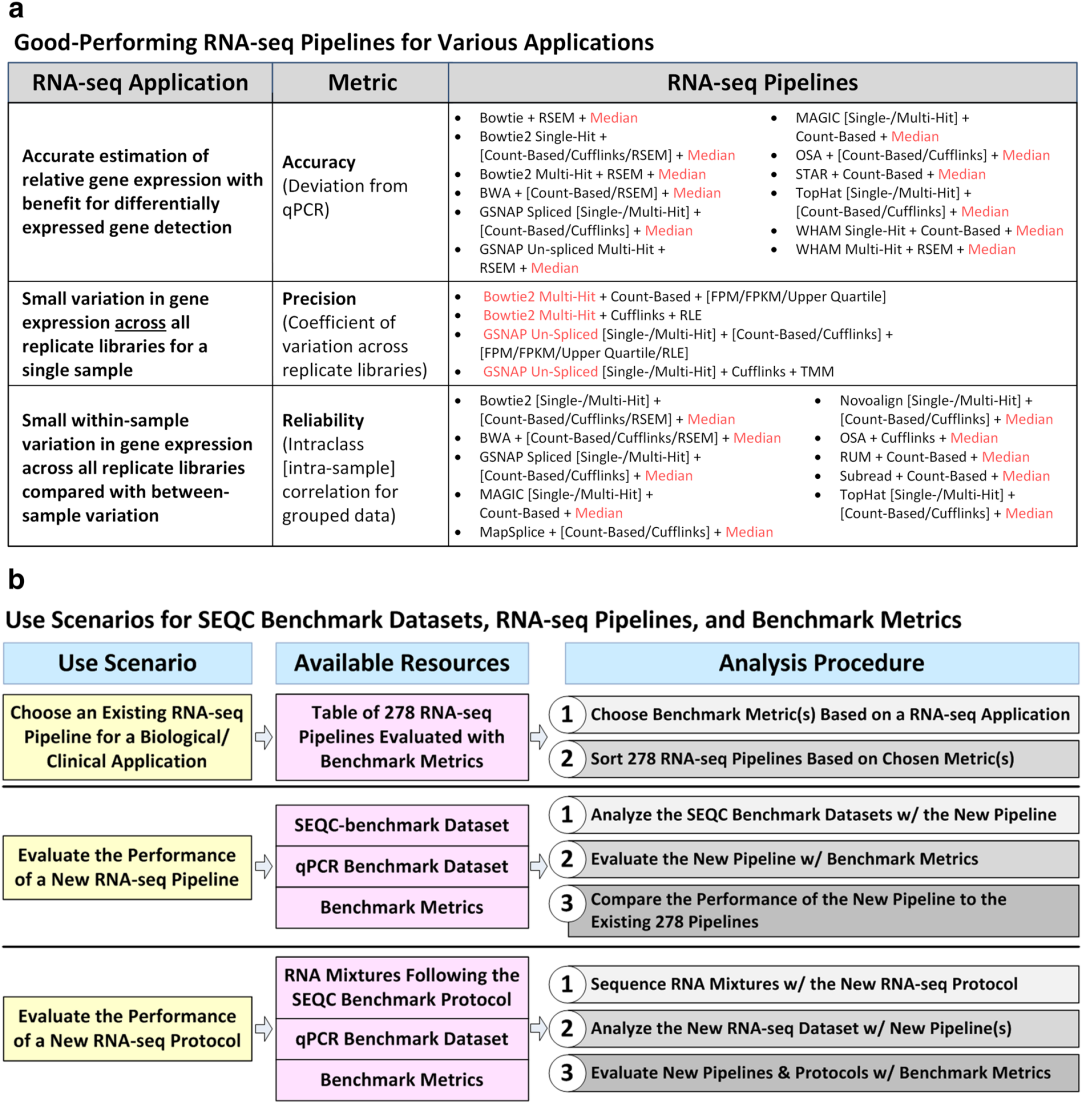
The resources provided by this study (i.e., the 278 RNA-seq pipelines, the benchmark metrics, and the SEQC-benchmark datasets) can serve as guidelines for biological and clinical researchers as well as for bioinformaticians and biotechnologists. (**a**) Depending on the gene expression application, the three metrics (i.e., accuracy, precision, and reliability) may be used to choose a pipeline. We have associated each metric with an RNA-seq application and listed the top-performing pipelines for each metric. The red-highlighted component in each listed RNA-seq pipeline indicates components that occur frequently among the top-performing pipelines for each metric. (**b**) Biological or clinical researchers who want to analyze Illumina RNA-seq data (or data from similar platforms with short, fixed-length reads) can choose an existing RNA-seq pipeline using the provided table of 278 pipelines ranked by accuracy, precision, or reliability. Bioinformaticians that are developing a new RNA-seq pipeline for Illumina data (or data from similar platforms) can use the SEQC-benchmark datasets and benchmark metrics to evaluate the new pipeline and assess its performance relative to the 278 pipelines. Bioinformaticians or biotechnologists that are developing new RNA-seq protocols can first sequence the same RNA mixture samples (i.e., samples A, B, C, and D), and then evaluate associated data analysis pipelines using the qPCR benchmark dataset and the benchmark metrics.

Putting our results in the context of real-world application, we offer the following scenarios by taking advantage of our findings:

In scenario 1 where researchers need to select a pipeline to analyze an Illumina dataset (or similar short-read sequence dataset), they may refer to Supplementary Table S8 to choose a pipeline by following these steps (Fig. 6):

1. Select a metric based on the requirements of the clinical application (not necessarily predictive modeling).
2. Sort the pipelines based on this metric and choose the top pipeline.

For example, depending on the application, different weights can be assigned to the three metrics instead of using the average rank. Researchers who want to conduct initial filtering of genes to identify DEGs may want to stress the importance of correct quantification of relative gene expression. Thus, they may want to focus on the accuracy metric. Top and bottom pipelines in terms of accuracy are listed in Fig. 6a. Median normalization is the frequently occurring component in the most accurate pipelines. If experimental conditions limit the sample size, small variation among replicate libraries may be important to better estimate gene expression. The precision and reliability metrics were designed to capture variation in gene expression from different perspectives. Researchers who need small variation in gene expression across replicate libraries for a single sample may want to weight the precision metric. Bowtie2 multi-hit and GSNAP un-splice mapping are the frequently occurring components in the most precise pipelines. Researchers who need small within-sample variation in gene expression relative to between-sample variation may want to emphasize on the reliability metric. Again, median normalization is the frequently occurring component in the most reliable pipelines.

In scenario 2 where researchers want to evaluate a newly developed RNA-seq pipeline for an Illumina dataset, they may use the SEQC-benchmark dataset, qPCR benchmark dataset, and the three metrics as follows (Fig. 6b):

1. Analyze the SEQC-benchmark dataset with the new RNA-seq pipeline.
2. Evaluate the new pipeline with the three metrics.
3. Compare the performance of the new pipeline to the 278 RNA-seq pipelines.

The 278 RNA-seq pipelines serve as a representative sample, or benchmark, of pipelines for gene expression estimation that include both good-performing and poor-performing pipelines.

In the third scenario, researchers who want to evaluate a new RNA-seq protocol may use the RNA samples from the SEQC protocol (i.e., samples A, B, C, and D), the qPCR benchmark dataset, and the three metrics as follows:

1. Sequence the RNA samples with the new RNA-seq protocol.
2. Analyze the new dataset with new pipelines specifically designed for the new RNA-seq protocol.
3. Evaluate the new pipelines and the new RNA-seq protocol with the three metrics.

Ideally, existing pipelines could be applied as fixed variables to both old and new RNA-seq protocols to attribute any changes in gene expression estimation performance to the change in RNA-seq protocols.

It is worthwhile to mention that, in the Phase-II experiment, we set a threshold to divide patients in each dataset into two groups to conduct downstream predictions. This choice is to balance the numbers of samples in two groups, and also to balance the computational complexity with the biological meaningfulness. With RNA-seq pipeline evaluation as the main focus of this study, we want to minimize the sample size imbalance introduced bias in the downstream biological applications. With that said, the investigation can be improved if the choice of threshold is more towards to the real-world clinical scenarios. Moreover, we did not investigate the underlying genes that contribute to the prediction. The biological and medical contexts of the gene signature are critical for the causality assessment and clinical application in explaining the disease outcome predictions. Thus, more sophisticated down-stream applications of RNA-seq can be explored in the future to evaluate the impact of RNA-seq pipelines.

In summary, we showed that upstream RNA-seq pipelines that performed well in gene expression estimation generally performed well for downstream gene expression-based prediction of disease outcome. Our study represents a large-scale and objective assessment of the predictive performance of various RNA-seq pipelines and should prove useful in moving RNA-seq-based predictive models closer to clinical applications.

## Methods

### FDA SEQC benchmark datasets

The FDA SEQC-benchmark dataset (Gene Expression Omnibus accession number GSE47792) includes paired-end RNA-seq data generated using the Illumina HiSeq 2000 platform with the read length of 100 nucleotides^7^. We used a subset of the SEQC-benchmark dataset sequenced at two sites—Beijing Genomics Institute (BGI) and Mayo Clinic (MAY). We used four samples (i.e., A, B, C, and D), each with four replicate libraries prepared at the sequencing sites. Sample A contains the Universal Human Reference RNA (UHRR), sample B contains the Human Brain Reference RNA (HBRR), sample C contains a mixture of A and B (75% A and 25% B), and sample D contains a mixture of A and B (25% A and 75% B). We used data from two lanes of a single flow cell for each sample replicate. The SEQC also provided a quantitative PCR (qPCR) benchmark dataset that includes 20,801 genes assayed with PrimePCR (Bio-Rad, Hercules, California). Each PrimePCR gene was assayed once for each of the four samples (i.e., A, B, C, and D). The FDA SEQC benchmark datasets and samples are summarized in Supplementary Tables S5 and S6.

### Neuroblastoma and lung adenocarcinoma datasets

We used a 176-sample neuroblastoma dataset (a subset of a larger 498-sample dataset; referred to as SEQC-neuroblastoma in this manuscript) to assess the performance of RNA-seq pipelines in terms of gene expression-based prediction of disease outcome. These samples were provided by the University Children’s Hospital of Cologne and sequenced at BGI using the Illumina platform^48^. All 176 samples were taken from high-risk patients that were defined as those either with stage 4 neuroblastoma and age > 18 months or with MYCN-amplified tumors of any stage or age. The SEQC-neuroblastoma dataset was deposited to the Gene Expression Omnibus with accession number GSE47792.

We predicted two clinical endpoints—event-free survival (EFS), that is, the occurrence of events such as progress, relapse, or death, and overall survival (OS), that is, death. For both endpoints, patients were partitioned into two groups (i.e., high risks versus low risks). High-risk patients experienced an event or died before a predefined survival-time threshold, while low-risk patients experienced an event or died after the threshold, or their last follow-up exceeded the threshold. Survival-time thresholds for EFS and OS were two and three years, respectively. The thresholds were chosen to balance the number of high-risk and low-risk patients. Details of the SEQC-neuroblastoma dataset are provided in Supplementary Table S9.

We also used an 87-sample lung adenocarcinoma RNA-seq dataset from The Cancer Genome Atlas (TCGA) repository. The prediction endpoint was also survival, and we used the same criteria to define high-risk and low-risk groups with the survival-time threshold of two years. The two-year threshold was chosen to balance the number of high-risk and low-risk patients. Details of the TCGA-lung-adenocarcinoma dataset are provided in Supplementary Table S10.

### Filtering the qPCR benchmark dataset to produce a reference set of genes

Because of variability in qPCR measurements and disagreements among qPCR platforms^7^, we filtered the qPCR benchmark dataset to retain genes that exhibited “correct” behavior. We then used these genes to calculate the benchmark metrics (i.e., accuracy, precision, reliability, and reproducibility). Such the filtering process is summarized in Supplementary Fig. S1.

Starting with the initial set of 20,801 genes assayed with PrimePCR, we filtered these genes to retain only genes that were quantified as non-zero (i.e., detected) and with Ct (cycle threshold) values ≤ 35 (35 indicates detection of only a single molecule in a sample). Filtering PrimePCR data resulted in 14,014 genes that also matched with the AceView transcriptome used for mapping the SEQC-benchmark RNA-seq dataset.

Subsequently, we filtered the 14,014 qPCR genes to retain only 12,610 genes that exhibited the correct titration order (TO) and expected mixing ratios (EMR). Details of this process are in the “Filtering qPCR genes by titration order and expected mixing ratios” section.

Lastly, since some benchmark metrics such as accuracy and precision are sensitive to zero- or very low-expressing genes, we further selected genes that were expressed as non-zero in all replicates of all samples of all sequencing sites and all 278 RNA-seq pipelines. The final reference set contains only 10,222 qPCR genes (referred to as “all genes”) that were used to compute all three benchmark metrics for RNA-seq pipelines.

Based on the previous study, the genes with lower expression are more likely to be inconsistent among pipelines^49^. Thus, we also identified a set of low-expressing genes in the 10,222 genes based on the average qPCR expression of samples A, B, C, and D. The lowest 20% of the 10,222 genes (i.e., 2044 genes, referred to as “low-expressing genes”) were also used to compute the same set of benchmark metrics for RNA-seq pipelines. This design enabled us to investigate the capability of RNA-seq pipelines in estimating low-expressing gene expression.

### Filtering qPCR genes by titration order and expected mixing ratios

The SEQC-benchmark datasets (RNA-seq and qPCR) have unique properties that enable the assessment of quantification correctness. After identifying detectable (i.e., non-zero and Ct ≤ 35) and AceView-matched qPCR genes, we used two metrics (TO and EMR) to further filter the benchmark qPCR dataset, leaving only “correct” qPCR genes. The TO and EMR metrics capture unique mixing properties of the data, that is,

$$C= \frac{3}{4}A+\frac{1}{4}B\, \text{and }\,= \frac{1}{4}A+\frac{3}{4}B.$$

Because of this property, all genes are expected to be expressed in one of the following orders, depending on the relative expression of samples A and B:

$$A\ge C\ge D\ge B \,\text{or }\, A\le C\le D\le B.$$

The TO metric determines if genes are expressed in the correct order. The expression value of a qPCR gene is defined as ${q}_{s,n,k}$, where $s\in \left\{A,B,C,D\right\}$ indicates the sample, $n=1\dots N$ indicates the replicate, and $k=1\dots K$ indicates the gene (for the PrimePCR dataset, *N* = 1 and *K* = 10,222). For a qPCR dataset with multiple replicates, given that the mean expression value for gene *k* and sample *s* over all replicates is

$${\stackrel{-}{q}}_{s,\cdot ,k}=\frac{1}{N}\sum_{n=1}^{N}{q}_{s,n,k,}$$

the set of qPCR genes that follow the correct titration order is

$${K}_{TO}=\left\{k|\left({\stackrel{-}{q}}_{A,\cdot ,k}\ge {\stackrel{-}{q}}_{C,\cdot ,k}\ge {\stackrel{-}{q}}_{D,\cdot ,k}\ge {\stackrel{-}{q}}_{B,\cdot ,k}\right)\vee \left({\stackrel{-}{q}}_{A,\cdot ,k}\le {\stackrel{-}{q}}_{C,\cdot ,k}\le {\stackrel{-}{q}}_{D,\cdot ,k}\le {\stackrel{-}{q}}_{B,\cdot ,k}\right)\right\}$$

For a single replicate qPCR dataset (e.g., the PrimePCR dataset we analyzed), the inherent variability of a single qPCR measurement may result in some false negative genes that follow the correct TO but fail to be identified. From the literature^50,51^, the coefficient of variation for replicate qPCR measurements is generally 15% or larger, so we used this number to adjust the margin for determining whether a gene follows the correct TO. Mathematically, we calculated the range of plus and minus one standard deviation from each qPCR measurement and used it as the margin. The revised equations for ${K}_{TO}$ are as follow:

$${K}_{TO,A\ge B}=\left\{k|\left(a\cdot {\stackrel{-}{q}}_{A,k}\ge {b\cdot \stackrel{-}{q}}_{C,k})\wedge (a{\cdot \stackrel{-}{q}}_{C,k}\ge {b\cdot \stackrel{-}{q}}_{D,k})\wedge (a\cdot {\stackrel{-}{q}}_{D,k}\ge {b\cdot \stackrel{-}{q}}_{B,k}\right)\right\}$$

$${K}_{TO,A\le B}=\left\{k|\left(b\cdot {\stackrel{-}{q}}_{A,k}\le {a\cdot \stackrel{-}{q}}_{C,k})\wedge (b{\cdot \stackrel{-}{q}}_{C,k}\le {a\cdot \stackrel{-}{q}}_{D,k})\wedge (b\cdot {\stackrel{-}{q}}_{D,k}\le {a\cdot \stackrel{-}{q}}_{B,k}\right)\right\}$$

$${K}_{TO}={K}_{TO,A\ge B}\cup {K}_{TO,A\le B,}$$

where $$a=1.15, b=0.85$$

Besides TO, samples should additionally exhibit a specific mixing ratio. Given that the ratio between samples A and B is

$${R}_{A,B}=\frac{A}{B}$$

the EMR between samples C and D is

$$EM{R}_{C,D}=\frac{3z\cdot {R}_{A,B}+1}{z\cdot {R}_{A,B}+3}\cdot \frac{z+3}{3z+1}$$

where $z=\frac{\textrm{mRNA \, Concentration \, in\, A}}{\textrm{mRNA \, Concentration\, in\, B}}=1.43$, a correction factor for the difference in mRNA concentration between samples A and B^7^.

The EMR metric examines if ${\text{EMR}}_{\text{C},\text{D}}$ of a gene is close enough to observed ${{\text{R}}_{{\text{C}},{\text{D}}}} = \frac{{\text{C}}}{{\text{D}}}$ of the same gene. As described previously, the PrimePCR dataset contains only a single measurement for each sample, and thus, wider margins are needed for EMR metric calculation. Using the same technique, we calculated the width of one standard deviation of each ratio as

$${R}_{A,B}\in \left[b\cdot {R}_{A,B}, a\cdot {R}_{A,B}\right]\equiv [{R}_{A,B}^{Lower},{ R}_{A,B}^{Upper} ],$$

$${R}_{C,D}\in \left[b\cdot {R}_{C,D}, a\cdot {R}_{C,D}\right]\equiv \left[{R}_{C,D}^{Lower},{ R}_{C,D}^{Upper} \right],\text{ and}$$

$$EM{R}_{C,D}\in \left[b\cdot {EMR}_{C,D}, a\cdot EM{R}_{C,D}\right]\equiv [{EMR}_{C,D}^{Lower},{ EMR}_{C,D}^{Upper} ]),$$

and finally determines a set of genes that satisfies the EMR criterion as follows:

$${K}_{EMR}=\left\{k|\left({{R}_{C,D}^{Lower}\le {EMR}_{C,D}^{Upper}|}_{{k, R}_{C,D}\ge EM{R}_{C,D}}\right)\vee \left({{R}_{C,D}^{Upper}\ge {EMR}_{C,D}^{Lower}|}_{{k, R}_{C,D}\le EM{R}_{C,D}}\right)\right\}$$

### RNA-seq data analysis pipelines—mapping, quantification, and normalization

We investigated 278 RNA-seq pipelines that included thirteen sequence mapping algorithms^18–29^, three categories of expression quantification algorithms^31–33^, and seven expression normalization methods. Supplementary Tables S2–S4 summarize all options considered for each pipeline component (sequence mapping, expression quantification, and expression normalization). The thirteen mapping algorithms investigated are Bowtie^18^, Bowtie2^19^, BWA^20^, GSNAP^21^, Magic^22^ (a new pipeline developed by NCBI for the SEQC project: ftp://ftp.ncbi.nlm.nih.gov/repository/acedb/Software/Magic), MapSplice^23^, Novoalign (a commercialized package developed by Novocraft: https://www.novocraft.com/products/novoalign/), OSA^24^, RUM^25^, STAR^26^, Subread^27^, TopHat^28^, and WHAM^29^. Some use un-spliced mapping of reads to the transcriptome, and some others perform spliced mapping to the genome. Magic uses both in parallel and compares the quality of each alignment to keep the best across multiple targets. Mapping algorithms may report only unique mapping, or allow for multiple mapping locations per read. Quantification algorithms include simple count-based methods (i.e., HTSeq^31^) and Poisson distribution-based probabilistic methods applied to either genomic (i.e., Cufflinks^32^) or transcriptomic mapping data (i.e., RSEM^33^). The Magic, RUM, and Subread (i.e., featureCounts^52^) pipelines include embedded quantification methods that fall into the category of simple count-based methods. Normalization methods include simple scaling methods (i.e., fragments per million mapped fragments [FPM], fragments per kilobase of gene length per million mapped fragments [FPKM], median, and upper quartile), robust scaling methods (i.e., relative log expression [RLE] and trimmed mean of m-values [TMM]), and methods embedded in specific pipelines (i.e., Magic expression index).

### Sequence mapping

We mapped sequences to each reference in successive steps using either un-spliced or spliced mapping algorithms. Un-spliced mapping refers to algorithms that align entire read sequences (e.g., Bowtie2, BWA, and Novoalign) whereas spliced mapping refers to algorithms that split reads into segments to accommodate long gaps or introns in a read (e.g., TopHat and MapSplice). In the first step of un-spliced mapping, we attempted to map all paired-end sequences to the ERCC/MT/rRNA reference (i.e., External RNA Controls Consortium sequences, the mitochondrial genome, and ribosomal RNA sequences). All unmapped read pairs were then mapped to the AceView transcriptome. Finally, all read pairs that did not map to either the ERCC/MT/rRNA or AceView references were mapped to the human genome reference. Transcriptomic mapping coordinates were then translated into genomic mapping coordinates and merged with the human genome mapping results to produce the final results (Supplementary Fig. S21, left panel). We used Bowtie2 as the mapper for the first step of all spliced mapping pipelines (Supplementary Fig. S21, right panel). Spliced mapping algorithms either directly mapped reads to the human genome (e.g., MapSplice and GSNAP) or mapped whole un-spliced reads to the transcriptome and then merged these mapping results with spliced mapping results of the remaining reads to the human genome (e.g., TopHat and OSA). Supplementary Table S2 summarizes all mapping tools investigated in this study.

Bowtie2, GSNAP, Novoalign, TopHat, and WHAM allow control over the number of reported mappings per read pair. By default, these algorithms typically report a single best mapping location per read pair. However, some quantification algorithms can use information about multiple ambiguous mapping locations to improve gene expression estimation. Thus, in addition to single-hit reporting, we also generated mapping results that reported up to 200 hits per read (multi-hit). We also included the Bowtie mapping pipeline with mapping parameters specific for quantification with RSEM, as described in the following section^33^.

Command-line options for all sequence alignment tools are detailed in Supplementary Note 1.

### Gene expression quantification

The quantification stage included three categories of quantifiers—count-based quantifiers (i.e., HTSeq and built-in quantifiers for the Magic, RUM, and Subread pipelines), probability model-based quantifiers for genomic mapping (i.e., Cufflinks), and probability model-based quantifiers for transcriptomic mapping (i.e., RSEM). The key characteristics of these quantifiers are summarized in Supplementary Table S3. Cufflinks is a Poisson model-based quantifier that estimates read assignment probabilities based on the alignment information^32^. It is capable of both assembling transcripts and quantifying gene or transcript expressions. In this study, we disabled the assembly function and provided the genome annotation GTF file as a quantification reference. HTSeq is a naïve count-based quantifier that assigns mapped reads to genes^31^. HTSeq is able to quantify gene expression, but not transcript expression. RSEM is also a Poisson model-based quantifier that is similar in concept to Cufflinks^33^. Information from multi-hit reads is important for both Cufflinks and RSEM. These algorithms use multi-hit read information to more accurately estimate gene or transcript expression.

Mapping results from alignment pipelines were not always compatible with the three categories of quantifiers. Cufflinks requires that alignment results are sorted by alignment coordinates and multi-hit reads are marked with the ‘NH’ tag in the attribute field of the SAM file. HTSeq requires that the alignment results are sorted by read names and that the ‘NH’ tag is absent in the SAM file. RSEM only quantifies transcriptomic mapping, that is, reads mapped and reported in transcriptomic coordinates. Moreover, RSEM only handles un-gapped alignments. Thus, filtering is required to remove gapped alignments. Because of these requirements, we pre-processed all alignment results before quantification. In summary, twenty alignment pipelines, including spliced, un-spliced, single-hit, and multi-hit pipelines, were suitable for count-based quantification. Sixteen alignment pipelines were suitable for Cufflinks, and only ten were suitable for RSEM. RSEM is specifically designed to work well with Bowtie. Thus, we also included this embedded mapping and quantification pipeline.

Command-line options for all quantification tools are detailed in Supplementary Note 1.

### Gene expression normalization

RNA-seq data normalization enables inter-sample comparison. Generally, normalization methods correct library size (i.e., the total number of reads in a sample), which is the primary source of inter-sample variance. We investigated seven normalization methods—fragments per million mapped fragments (FPM), fragments per kilobase of gene length per million mapped fragments (FPKM), median (Med.), upper quartile (UQ), relative log expression (RLE), trimmed mean of M-values (TMM), and expression index (EIndex, which is specific to the Magic pipeline) (see Supplementary Table S4). We describe each of these normalization methods based on the following mathematical description of the SEQC-benchmark dataset.

The raw count of a sample is defined as $x_{s,n,k}$, where $s \in \left\{ {A,B,C,D} \right\}$ indicates the sample, $n = 1 \ldots N$ indicates the replicate, and $k = 1 \ldots K$ indicates the gene. For the SEQC-benchmark dataset, *N* = 4 and *K* = 55,874. We normalized data within each of the two sites (i.e., BGI and MAY), each of which contains 16 samples (four replicates for each of the four samples). Because we used AceView as the transcriptome reference, a large portion of the genes had no counts. Moreover, the number of genes with zero counts was highly variable across samples and replicates. Thus, for each RNA-seq pipeline, we identified and used only present genes during normalization. Given that the mean of the counts for gene *k* and sample *s* over all replicates is

$$\overline{x}_{s, \cdot ,k} = \frac{1}{N}\mathop \sum \limits_{n = 1}^{N} x_{s,n,k}$$

we defined the set of present genes to be

$$K_{p,BGI} \in \left \{ k|\left (\overline{x}_{A, \cdot ,k} > 1 \vee \overline{x}_{B, \cdot ,k} > 1 \right ) \wedge \overline{x}_{C, \cdot ,k} > 1 \wedge \overline{x}_{D, \cdot ,k} > 1 \right \}, {\text{where }}x_{s,n,k} {\text{ is from the BGI site}};$$

$$K_{p,MAY} \in \left \{ k|\left (\overline{x}_{A, \cdot ,k} > 1 \vee \overline{x}_{B, \cdot ,k} > 1 \right ) \wedge \overline{x}_{C, \cdot ,k} > 1 \wedge \overline{x}_{D, \cdot ,k} > 1 \right \}, {\text{where }}x_{s,n,k} {\text{ is from the MAY site}};$$

and the final present gene set is

$$K_{p} = K_{p,BGI} \cap K_{p,MAY} .$$

We used the same set of present gens for all normalization methods for a RNA-seq pipeline.

The total count of present genes for a given sample *s* and replicate *n* is

$$x_{s,n} = \mathop \sum \limits_{{k \in K_{p} }} x_{s,n,k} ,$$

and the average total count of present genes for all data from a single site is

$$\bar{x} = \frac{1}{4}\frac{1}{N}\mathop \sum \limits_{s} \mathop \sum \limits_{{n = 1}}^{N} x_{{s,n}}.$$

Thus, we defined FPM-normalized expression for each sample *s*, replicate *n*, and gene *k* as

$$y_{s,n,k}^{FPM} = \frac{{x_{s,n,k} \cdot \overline{x}}}{{x_{s,n} }}.$$

Similarly, we defined $\tilde{x}_{s,n}$ and $\hat{x}_{s,n}$ as the median and upper quartile of counts, respectively, of all present genes in sample *s* and replicate *n*:

$$\tilde{x} = \frac{1}{4}\frac{1}{N}\mathop \sum \limits_{s} \mathop \sum \limits_{n = 1}^{N} \tilde{x}_{s,n} {\text{ and }}\hat{x} = \frac{1}{4}\frac{1}{N}\mathop \sum \limits_{s} \mathop \sum \limits_{n = 1}^{N} \hat{x}_{s,n} .{ }$$

Median- and upper-quartile-normalized expression for each sample *s*, replicate *n*, and gene *k* are then defined as

$$y_{s,n,k}^{Med} = \frac{{x_{s,n,k} \cdot \tilde{x}}}{{\tilde{x}_{s,n} }}{\text{and }}y_{s,n,k}^{UQ} = \frac{{x_{s,n,k} \cdot \hat{x}}}{{\hat{x}_{s,n} }}.{ }$$

For FPKM normalization, we defined the length of a gene *k* as $\ell_{k}$, which is the length of the union of all exons related to the gene as defined by the AceView transcriptome. The original formulation of FPKM arbitrarily used scaling factors of 1 × 10^3^ for the gene length and 1 × 10^6^ for the total number of mapped fragments. In order to maintain comparable dynamic range among all normalization methods, we instead scaled by the average gene length and average total count for all present genes. The average length of all present genes is

$$\overline{\ell } = \frac{1}{{\left| {K_{p} } \right|}}\mathop \sum \limits_{{k \in K_{p} }} \ell_{k} .$$

Thus, rescaled FPKM-normalized expression for each sample *s*, replicate *n*, and gene *k* is

$$y_{s,n,k}^{FPKM} = \frac{{x_{s,n,k} \cdot \overline{\ell } \cdot \overline{x}}}{{x_{s,n} \cdot \ell_{k} }}.$$

The TMM and RLE normalization methods are similar to the FPM normalization, but introduce an additional scaling factor to adjust the library size. We used the edgeR package in R to estimate a scaling factor for each sample replicate^36,53^. The TMM method selects a reference library from a pool of sample replicate libraries and then calculates gene-wise log expression ratios (M-values) and gene-wise average log expression values (A-values) between the target library and the reference library. Extreme numbers in M-values and A-values are trimmed, and the scaling factor for the target library is the weighted average of remaining M-values. The RLE method determines a scaling factor by first defining the median library as the gene-wise geometric mean across sample replicates^35^. The median ratio of each target library to the median library is taken as the scaling factor. TMM- and RLE-normalized expression for each sample *s*, replicate *n*, and gene *k* are then defined as:

$$y_{s,n,k}^{TMM} = \frac{{x_{s,n,k} \cdot \overline{x}}}{{x_{s,n} \cdot \hat{f}_{s,n}^{TMM} }}{\text{and }}y_{s,n,k}^{RLE} = \frac{{x_{s,n,k} \cdot \overline{x}}}{{x_{s,n} \cdot \hat{f}_{s,n}^{RLE} }}, {\text{respectively}},$$

where $\hat{f}_{s,n}^{TMM}$ and $\hat{f}_{s,n}^{RLE}$ are the scaling factor for sample *s*, replicate *n*.

### RNA-seq pipeline performance metrics

Benchmark metrics for RNA-seq pipelines are summarized in Supplementary Table S7.

### Accuracy measured as deviation from qPCR references

We measured deviation of RNA-seq pipeline-derived inter-sample log ratios from qPCR-based inter-sample log ratios, and defined such the deviation as the accuracy metric. We previously defined qPCR assayed gene expression as ${q}_{s,n,k},$ where $s\in \left\{A,B,C,D\right\}$ indicates the sample, $n=1\dots N$ indicates the replicate, and $k=1\dots K$ indicates the gene (for the PrimePCR dataset, *N* = 1 and *K* = 10,222). The mean expression of a qPCR gene *k* in sample *s* over all replicates is

$${\stackrel{-}{y}}_{s,\cdot ,k}=\frac{1}{N}\sum_{n=1}^{N}{y}_{s,n,k}$$

Given samples A and B, the absolute log-ratio deviation of RNA-seq-based expression from qPCR-based expression for a gene *k* is

$$\Delta_{\frac{A}{B},k} = \left | \log_2\left ( \frac{\bar{x}_{A,.,k}}{\bar{x}_{B,.,k}} \right ) - \log_2 \left ( \frac{\bar{q}_{A,.,k}}{\bar{q}_{B,.,k}} \right ) \right |,$$

and the final accuracy metric was defined as the median of all ${\Delta }_{{\frac{A}{B},k}}$, $k = 1 \ldots K$.

### Precision measured as variation of gene expression across replicate libraries

We computed the coefficient of variation (CoV) for each gene and each sample across four replicate libraries as follows:

$$CoV_{s,k} = \frac{{sd\left( {x_{s, \cdot ,k} } \right)}}{{\overline{x}_{s, \cdot ,k} }},$$

where $k = 1 \ldots K$ indicates the gene and $s \in \left\{ {A,B,C,D} \right\}$ indicates the sample. We then used the median of all CoVs across all genes and all samples as the final measure of precision.

### Reliability measured as intra-sample correlation of gene expression

The reliability of a measurement system can be assessed by the intraclass correlation coefficient (ICC)^54,55^. ICC is applicable to measurements that can be organized into groups, and it describes how similar measurements of the same group are to one another. Modern ICC definition borrows the framework of analysis of variance (ANOVA), or more specifically ANOVA with random effects^55^. The type of ANOVA depends on the experimental design and generally follows the definition in Shrout’s article published in 1979^55^. ICC(1,1) and ICC(1,k) are based on the one-way random effects model and are applicable to the case that each group is assessed by a different set of k raters randomly selected from a larger population of raters. ICC(2,1) and ICC(2,k) are based on the two-way random effects model and are applicable to the case that a random sample of k raters is preselected from a larger population and each rater assesses each group exactly once (i.e., each rater assesses n groups altogether). ICC(3,1) and ICC(3,k) are based on the two-way mixed effects model and are applicable to the case that each group is assessed by each of the same k raters, who are the only raters in the population. The second parameter in ICC([1,2,3],[1,k]) denotes whether the ICC is to measure the reliability of a single measurement or the average of k measurements.

For the SEQC benchmark dataset with replicate libraries for each sample, ICC(1,1) or ICC(1,k) fitted our objective since, for a gene *g*, gene expression of replicate libraries for different samples (or different groups in the previous context) were not assessed under exactly the same conditions (or assessed by the same raters in the previous context). We chose to use ICC(1,k) as replicate libraries are available for most experiments. Mathematically, a one-way random effects model can be formulated as

$$Y_{ij} = \mu + \alpha_{j} +{\varepsilon_{ij}} ,$$

where $Y_{ij}$ is the *i*th observation in the *j*th group, ${\upmu }$ is an unobserved overall mean, $\alpha_{j}$ is the group-specific random effect with zero mean and variance $\sigma_{\alpha }^{2}$, and $\varepsilon_{ij}$ is an unobserved noise term with zero mean and variance ${\sigma_{\varepsilon}^{2}}$. The formula for ICC(1,k) is as follows:

$$ICC\left( {1,k} \right) = \frac{BMS - WMS}{{BMS}},$$

where BMS stands for the between groups mean square formulated as $k\sigma_{\alpha }^{2} + {\sigma_{\varepsilon}^{2}}$ and WMS stands for the within group mean square formulated as ${\sigma_{\varepsilon}^{2}}$. Higher ICC indicates higher reliability.

We calculated ICC for each gene *k*, $k = 1 \ldots K$, and then used the median of all ICCs as the final measure of reliability.

We have also investigated other potential metrics, such as reproducibility, which is defined as the Spearman correlation between two replicate libraries of the same sample (Supplementary Note 2). The Spearman correlation ranged from 0.993 to 0.996 (Supplementary Fig. S8) using AllGenes. We discarded the reproducibility metric because of the relatively small dynamic range.

### Evaluating the utility of the benchmark metrics for RNA-Seq pipeline selection

We ranked RNA-seq pipelines base on the average rank of the three benchmark metrics (i.e., accuracy, precision, and reliability). We then evaluated the utility of the benchmark metrics by examining whether good-performing and poor-performing pipelines identified based on the benchmark metrics were informative for inferring the performance of gene-expression-based prediction of disease outcome and statistical significance of patient stratification for all clinical endpoints (i.e., the SEQC-neuroblastoma EFS and OS endpoints and the TCGA-lung-adenocarcinoma survival endpoint).

First, for the 278 representative RNA-seq pipelines applied to the SEQC-benchmark dataset, we computed the average rank using a subset of the benchmark metrics as the final performance indicator for each pipeline. In total, we had 6 metrics (3 benchmark metrics [accuracy, precision, reliability] × 2 gene sets [2044 low-expressing genes, 10,222 all genes]), and we investigated 12 subsets (4 × 3) of the 6 metrics using the following criteria:

1. Four combinations of the three benchmark metrics with at least two in a subset—one combination with all three benchmark metrics, three combinations with two out of the three benchmark metrics.
2. Three subsets formed by metrics derived from all genes, those derived from low-expressing genes, or a combination of both.

Second, for each of the 278 representative RNA-seq pipelines (156 for the TCGA-lung-adenocarcinoma survival endpoint), we calculated nested cross-validation AUC and MCC, as described in the “Method” section “Neuroblastoma and lung adenocarcinoma predictive modeling,” resulting in 834 (468 for the TCGA-lung-adenocarcinoma survival endpoint) AUC and MCC values for each clinical endpoint (i.e., 278 pipelines × 3 classifiers, or 156 pipelines × 3 classifiers) (Supplementary Tables S11,S12). We also modeled survival functions using Kaplan–Meier analysis for each pipeline, as described in the “Method” section “Kaplan–Meier survival analysis”. For each RNA-seq pipeline, we summarized the performance of gene-expression-based prediction of disease outcome using both the average AUC and MCC across classifiers and the success rate of patient stratification (i.e., statistically significant separation of two Kaplan–Meier curves) across all iterations and classifiers in the nested cross-validation framework.

Finally, we identified the top 10% good-performing pipelines and the bottom 10% poor-performing pipelines based on the average rank of a subset of the three benchmark metrics. The corresponding prediction performance (i.e., AUC and MCC) of the good-performing pipelines was tested against that of the poor-performing pipelines using the one-sided Wilcoxon rank-sum test with the null hypothesis that the median of the former group was not larger than that of the latter group.

### Neuroblastoma and lung adenocarcinoma predictive modeling

We assessed the performance of 278 RNA-seq pipelines in terms of gene expression-based decision-making using the SEQC-neuroblastoma dataset^48^. The SEQC-neuroblastoma dataset and associated clinical endpoints are summarized in Supplementary Table S9. The RNA-seq pipelines were evaluated in terms of predicting neuroblastoma patient outcomes for two clinical endpoints using nested cross-validation (Supplementary Fig. S13)^56,57^. We also similarly assessed the performance of 156 RNA-seq pipelines applied to the TCGA-lung-adenocarcinoma dataset to predict disease outcome. The TCGA-lung-adenocarcinoma dataset and the associated clinical endpoint are summarized in Supplementary Table S10.

Nested cross-validation involves training and testing of an optimal prediction model. This is accomplished using the three-fold optimizing or inner cross-validation, applied to the training subset from the fivefold outer cross-validation. Once the final optimal prediction model parameters (i.e., the classifier hyperparameters and feature size) are identified, the final model is trained using the entire training subset, and then tested using the remaining fold from the fivefold outer cross-validation. This process was repeated for ten iterations. We conducted nested cross-validation separately for each of the three classifiers (i.e., adaptive boosting, logistic regression, and support vector machines) and used the minimum redundancy, maximum relevance (mRMR) feature selection method to choose optimal feature sizes from within the range of 5 to 40 with the step size of 5^58^.

### Kaplan–Meier survival analysis

For each RNA-seq pipeline and classifier (i.e., 278 pipelines × 3 classifiers for the SEQC-neuroblastoma endpoints and 156 pipelines × 3 classifiers for the TCGA-lung-adenocarcinoma survival endpoint), we modeled Kaplan–Meier survival functions based on the predicted labels of each sample. We then used the two-tailed log-rank test to determine if estimated survival curves for each predicted patient group were statistically different.

### Analysis of variance and calculation of the contribution of each RNA-seq pipeline factor to the overall pipeline variance

We used analysis of variance (ANOVA) to determine if each RNA-seq pipeline factor significantly contributes to the variance of each of the three benchmark metrics (i.e., accuracy, precision, and reliability) as well as to the variance of prediction performance (i.e., AUC and MCC). For each of the three benchmark metrics, we used a linear model (R function “lm”) to fit the data from all 278 pipelines using the metric as the dependent variable and the RNA-seq pipeline factors as independent categorical variables. We considered the following factors as independent categorical variables—mapping algorithm, mapping strategy (i.e., spliced vs. un-spliced), mapping reporting (i.e., single-hit vs. multi-hit), quantification algorithm, and normalization algorithm. We included all factors and their two-way interactions in the linear model. For each of the prediction endpoints, we applied the same technique to fit the data from all 278 pipelines using average AUC or MCC as the dependent variable and the same set of RNA-seq pipeline factors as independent categorical variables. We then conducted the ANOVA on the linear model (R function “anova”). ANOVA calculates a “sum of squares” (i.e., variance) attributed to each factor or interaction and uses an F-test to determine if the variance is statistically significant. We calculated the percent that each factor or interaction contributes to the total variance by calculating the ratio of “sum of squares” for each factor to the total sum of squares.

### Regression analysis

We investigated the relationship between alignment profiles or gene expression distribution characteristics and benchmark metrics. The alignment profiles included the total number of mapped fragments, the total number of reads spanning the intronic region, the total number of reads with insertions or deletions, the total number of perfectly matched reads, the total number of reads with at most one mismatch, and the number of mismatches per mapped read. Each alignment algorithm was represented by the average statistics over 2 sequencing sites, 4 samples, 4 replicate libraries, and 2 lanes. Using the “MASS” package in R, we adopted the M-estimation with Huber weighting approach to fit robust linear regression models between a dependent variable (benchmark metric performance) and an explanatory variable (an alignment profile). The M-estimation with Huber weighting approach is a regression method that is robust in the presence of outliers. The gene expression distribution characteristics included the lower quartile, median, upper quartile, maximum, interquartile range, standard deviation, skewness, kurtosis, and entropy of a gene expression distribution. We used the same M-estimation with Huber weighting approach to fit robust linear regression models, and then reported the residual standard error for each model.

### Disclaimer

The views presented in this article do not necessarily reflect current or future opinion or policy of the US Food and Drug Administration. Any mention of commercial products is for clarification and not intended as an endorsement.

## Supplementary information

Supplementary Information 1.

Supplementary Table 8

Supplementary Table 11

Supplementary Table 12

Supplementary Table 13

Supplementary Table 14
