# Supplementary material for: Impact of RNA-seq data analysis algorithms on gene expression estimation and downstream prediction
Source: Sci Rep. 2020 Oct 21;10:17925. doi: 10.1038/s41598-020-74567-y (PMC7578822; doi:10.1038/s41598-020-74567-y)
Supplement: Supplementary file 1 — Supplementary Information 1. [file 41598_2020_74567_MOESM1_ESM.docx]

**Impact of RNA-seq Data Analysis Algorithms on Gene Expression Estimation and Downstream Prediction**

**Li Tong^1,6^, Po-Yen Wu^2,6^, John H. Phan^1,6^, Hamid R. Hassazadeh^3^, SEQC Consortium^4^, Weida Tong^5,7^ & May D. Wang^1,7^**

^1^Department of Biomedical Engineering, Georgia Institute of Technology and Emory University, Atlanta, Georgia, USA

^2^School of Electrical and Computer Engineering, Georgia Institute of Technology, Atlanta, Georgia, USA

^3^School of Computational Science and Engineering, Georgia Institute of Technology, Atlanta, Georgia, USA

^4^A full list of authors and their affiliations appears at the end of the file

^5^National Center for Toxicological Research, US Food and Drug Administration, Jefferson, Arkansas, USA

^6^These authors contributed equally to this work

^7^These authors are co-corresponding senior authors

Correspondence should be addressed to M.D.W. ([maywang@bme.gatech.edu](mailto:maywang@bme.gatech.edu))

**Supplementary**

**Page**

[Supplementary Figure 1: Filtering Benchmark qPCR Genes. 3](#_Toc467078780)

[Supplementary Figure 2: Median Accuracy of Low-Expressing Genes. 4](#_Toc467078781)

[Supplementary Figure 3: ANOVA for the Median Accuracy of Low-Expressing Genes. 5](#_Toc467078782)

[Supplementary Figure 4: Median Precision of Low-Expressing Genes. 6](#_Toc467078783)

[Supplementary Figure 5: ANOVA for the Median Precision of Low-Expressing Genes. 7](#_Toc467078784)

[Supplementary Figure 6: Median Reliability of Low-Expressing Genes. 8](#_Toc467078785)

[Supplementary Figure 7: ANOVA for the Median Reliability of Low-Expressing Genes. 9](#_Toc467078786)

[Supplementary Figure 8: Median Reproducibility of All Genes. 10](#_Toc467078787)

[Supplementary Figure 9: ANOVA for the Median Reproducibility of All Genes. 11](#_Toc467078788)

[Supplementary Figure 10: Median Reproducibility of Low-Expressing Genes. 12](#_Toc467078787)

[Supplementary Figure 11: ANOVA for the Median Reproducibility of Low-Expressing Genes. 13](#_Toc467078788)

[Supplementary Figure 12: Relationship between Alignment Profiles and Benchmark Metrics. 14](#_Toc467078789)

[Supplementary Figure 13: Predictive Modeling Procedure Using Nested Cross-Validation. 15](#_Toc467078790)

[Supplementary Figure 14: Prediction Performance of NB EFS Measured by AUC and MCC. 16](#_Toc467078791)

[Supplementary Figure 15: Prediction Performance of NB OS Measured by AUC and MCC. 17](#_Toc467078792)

[Supplementary Figure 16: Prediction Performance of LUAD Survival Measured by AUC and MCC. 18](#_Toc467078793)

[Supplementary Figure 17: ANOVA for Prediction Performance of NB EFS. 19](#_Toc467078794)

[Supplementary Figure 18: ANOVA for Prediction Performance of NB OS. 20](#_Toc467078795)

[Supplementary Figure 19: ANOVA for Prediction Performance of LUAD Survival. 21](#_Toc467078796)

[Supplementary Figure 20: The Impact of RNA-seq Pipeline Choices on Sequence Mapping and Expression Quantification Outcome. 22](#_Toc467078797)

[Supplementary Figure 21: Un-spliced and Spliced RNA-seq Mapping Pipelines. 23](#_Toc467078798)

[Supplementary Table 1: RNA-seq Pipelines. 24](#_Toc467078799)

[Supplementary Table 2: RNA-seq Mapping Tools. 25](#_Toc467078800)

[Supplementary Table 3: RNA-seq Gene Expression Quantification Tools. 26](#_Toc467078801)

[Supplementary Table 4: RNA-seq Gene Expression Normalization Methods. 27](#_Toc467078802)

[Supplementary Table 5: SEQC Benchmark Datasets. 28](#_Toc467078803)

[Supplementary Table 6: SEQC Benchmark Samples. 28](#_Toc467078804)

[Supplementary Table 7: RNA-seq Pipeline Metrics. 28](#_Toc467078805)

[Supplementary Table 8: Benchmark Metric Performance for the 278 RNA-seq Pipelines. 29](#_Toc467078806)

[Supplementary Table 9: Prediction Endpoints for the SEQC Neuroblastoma Dataset. 29](#_Toc467078807)

[Supplementary Table 10: Prediction Endpoint for the TCGA Lung Adenocarcinoma Dataset. 29](#_Toc467078808)

[Supplementary Table 11: Predictive Modeling Performance for the SEQC Neuroblastoma Dataset. 30](#_Toc467078809)

[Supplementary Table 12: Predictive Modeling Performance for the Lung Adenocarcinoma Dataset. 30](#_Toc467078810)

[Supplementary Table 13: Predictive Modeling Performance Measured by the Patient Stratification Success Rate (P-value Threshold=0.05). 30](#_Toc467078811)

[Supplementary Table 14: Mapping Statistics of SEQC-Benchmark Dataset. 30](#_Toc467078812)

[Supplementary Table 15: Comparison of the SEQC Pipeline Study to Previous Studies. 31](#_Toc467078813)

[Supplementary Note 1: Sequence Mapping and Expression Quantification Protocols. 35](#_Toc467078814)

[Supplementary Note 2: Reproducibility Measured as Inter-replicate Correlation of Gene Expression. 40](#_Toc467078814)


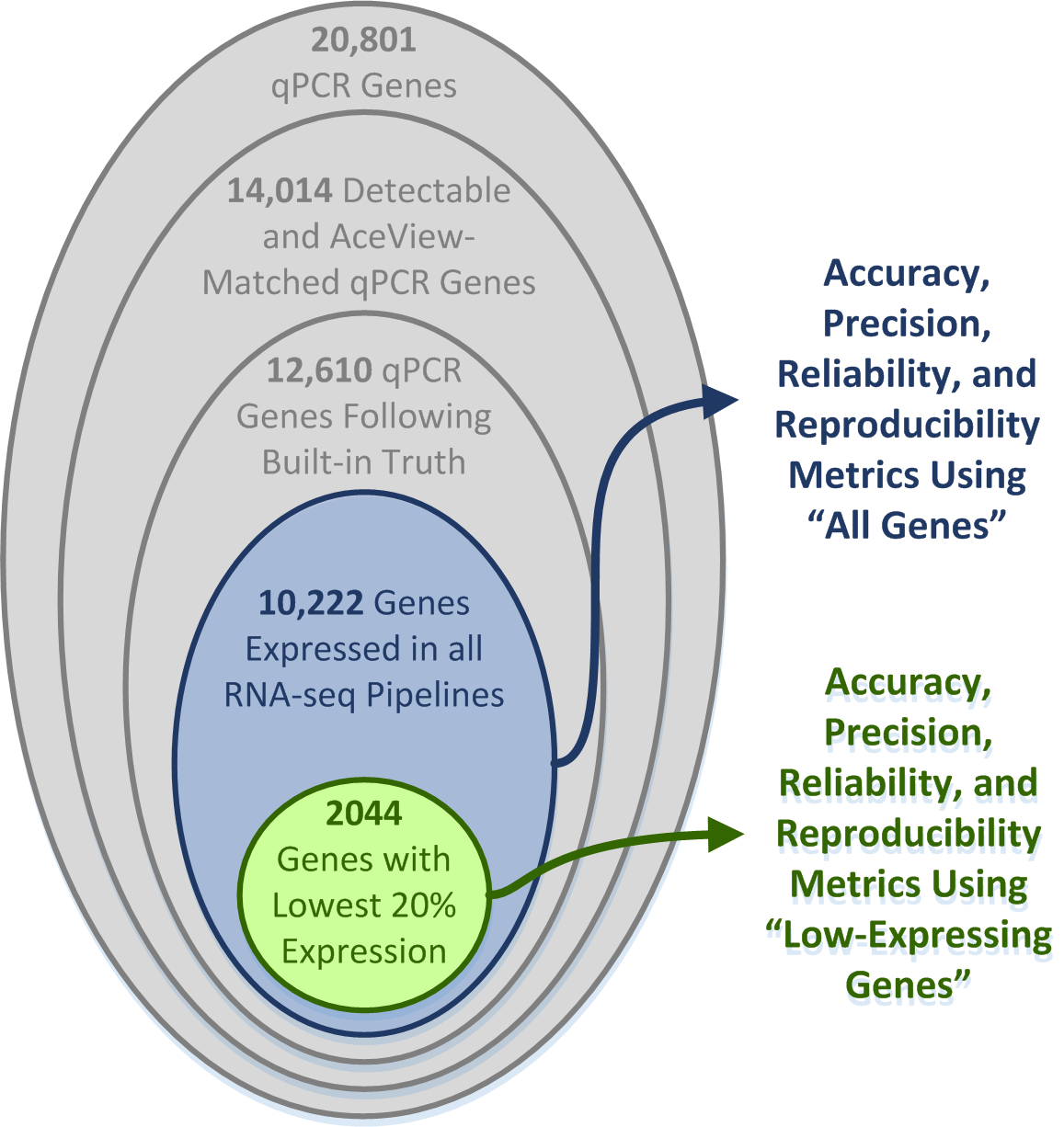


# Supplementary Figure 1: Filtering Benchmark qPCR Genes.

The 20,801 qPCR genes are first filtered to retain only 14,014 genes that are detectable (i.e.,${0<qPCR Cycle Threshold C}_{t}\leq35$) and that match genes in the AceView transcriptome. Subsequently, genes are filtered to retain only the 12,610 genes that exhibit good titration order and expected mixing ratios. Finally, 10,222 genes (denoted as “All Genes”) that expressed (i.e.,$Gene Expression>0$) in all replicates of all samples of all sequencing sites of all 278 RNA-seq pipelines are retained for evaluation metric calculation (i.e., accuracy, precision, reliability, and reproducibility). Among 10,222 genes, the 20% lowest-expressing genes (i.e., 2,044 genes; denoted as “Low-Expressing Genes”) based on the average qPCR measurement of samples A, B, C, and D are selected for calculating the same set of evaluation metrics.

**
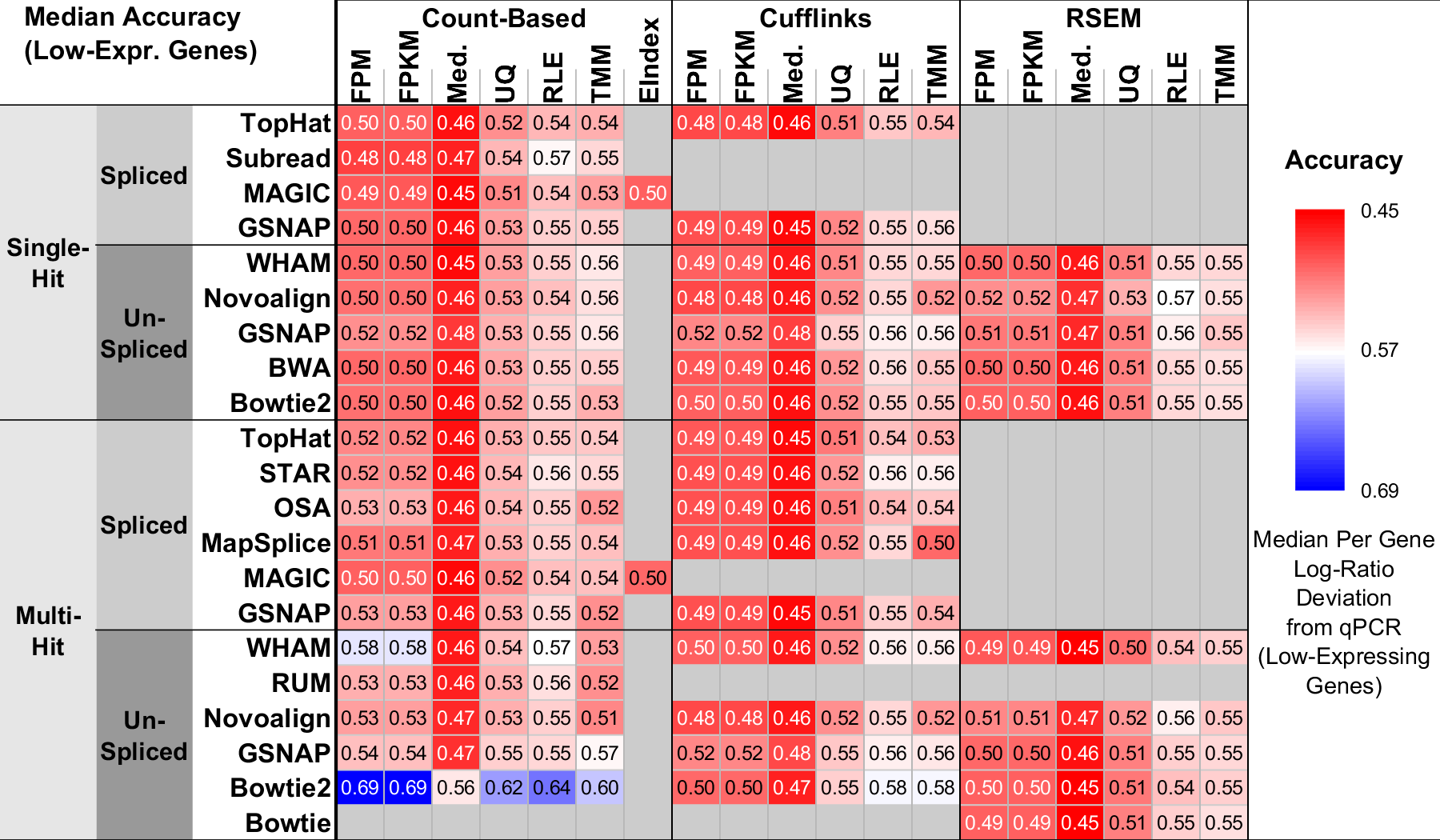
**

# Supplementary Figure 2: Median Accuracy of Low-Expressing Genes.

The 278 RNA-seq pipelines applied to the SEQC-benchmark dataset differ in the median accuracy of low-expressing genes. Accuracy is defined as the deviation of pipeline-derived log ratios from the corresponding qPCR-based log ratios. It is encoded as color, with red representing the highest accuracy, or the lowest deviation from qPCR. “Low-Expressing Genes” refers to the 2,044 qPCR genes after filtering.

**
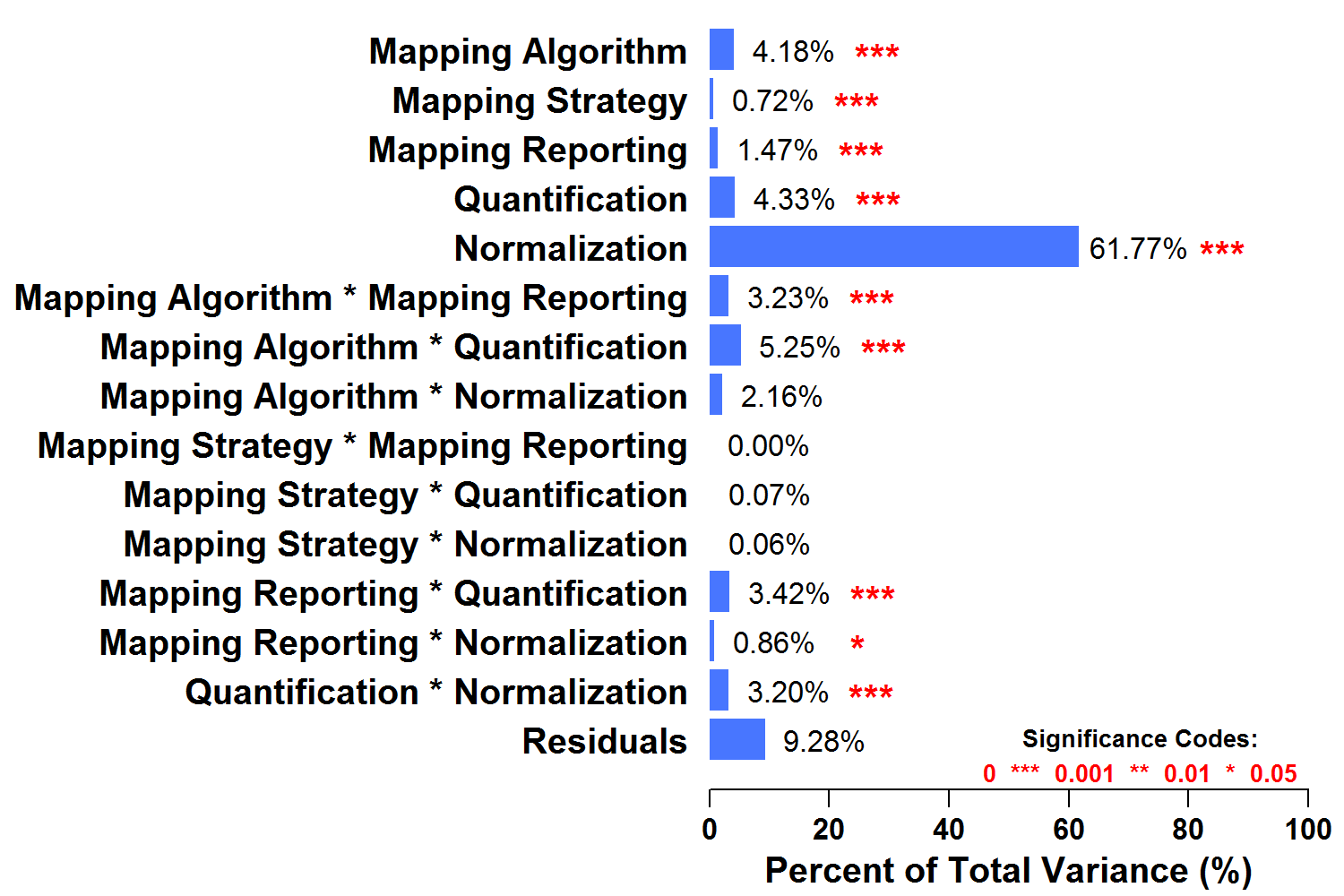
**

# Supplementary Figure 3: ANOVA for the Median Accuracy of Low-Expressing Genes.

Analysis of variance (ANOVA) decomposes the overall variance in the median accuracy of low-expressing genes into various factors considered, including RNA-seq pipeline components and associated two-way interactions. The statistical significance of the contribution of each component and interaction is denoted by red asterisks, with ‘***’ indicates p-values are smaller than 0.001, ‘**’ indicates p-values are smaller than 0.01, and ‘*’ indicates p-values are smaller than 0.05. Among all components and interactions, the normalization contributes the most to the overall variance.

**
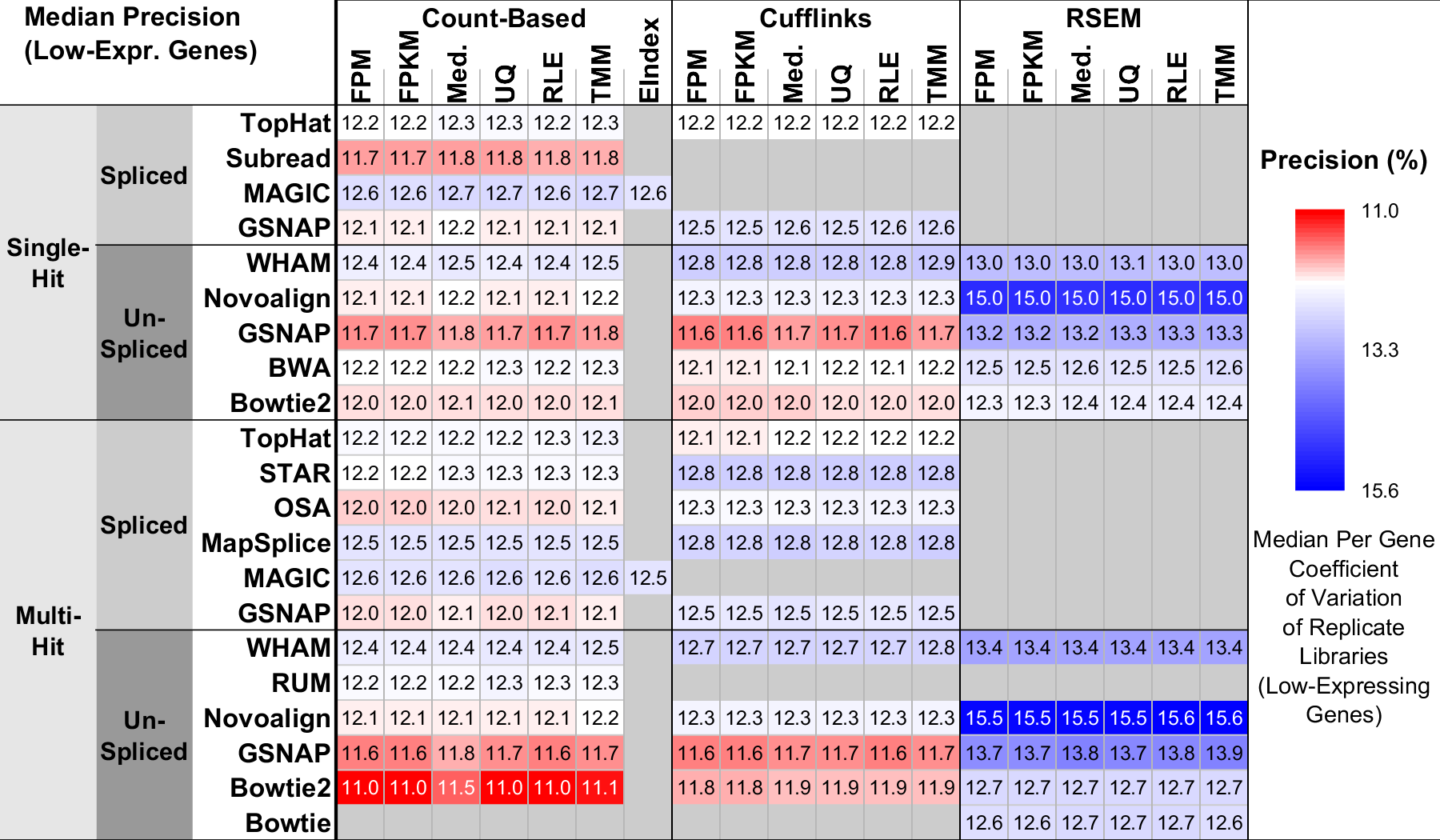
**

# Supplementary Figure 4: Median Precision of Low-Expressing Genes.

The 278 RNA-seq pipelines applied to the SEQC-benchmark dataset differ in the median precision of low-expressing genes. Precision is defined as the coefficient of variation across replicate libraries. It is encoded as color, with red representing the highest precision, or the lowest coefficient of variation. “Low-Expressing Genes” refers to the 2,044 qPCR genes after filtering.

**
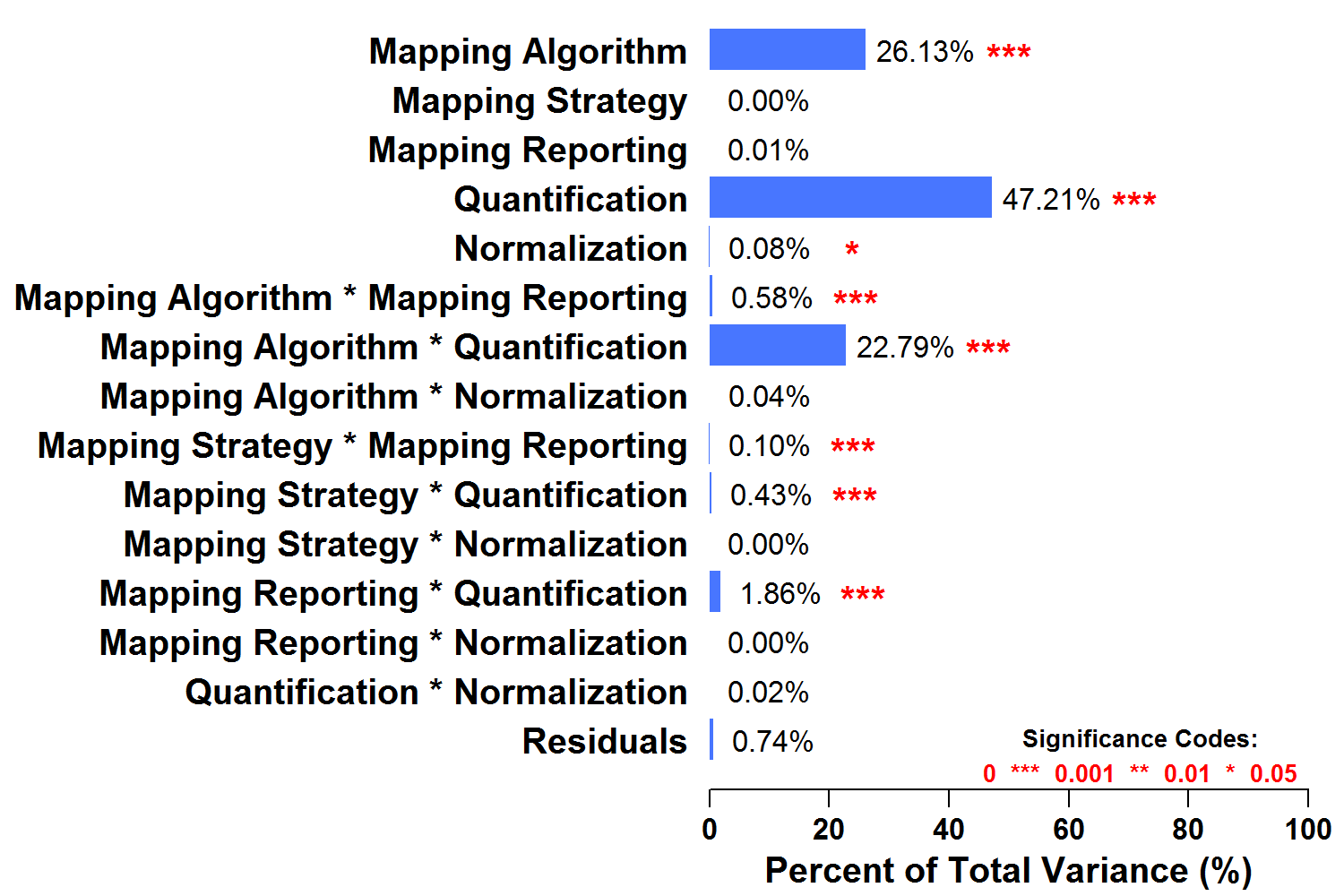
**

# Supplementary Figure 5: ANOVA for the Median Precision of Low-Expressing Genes.

Analysis of variance (ANOVA) decomposes the overall variance in the median precision of low-expressing genes into various factors considered, including RNA-seq pipeline components and associated two-way interactions. The statistical significance of the contribution of each component and interaction is denoted by red asterisks, with ‘***’ indicates p-values are smaller than 0.001, ‘**’ indicates p-values are smaller than 0.01, and ‘*’ indicates p-values are smaller than 0.05. Among all components and interactions, the quantification, mapping algorithm, and mapping algorithm-quantification interaction contribute the most to the overall variance.

**
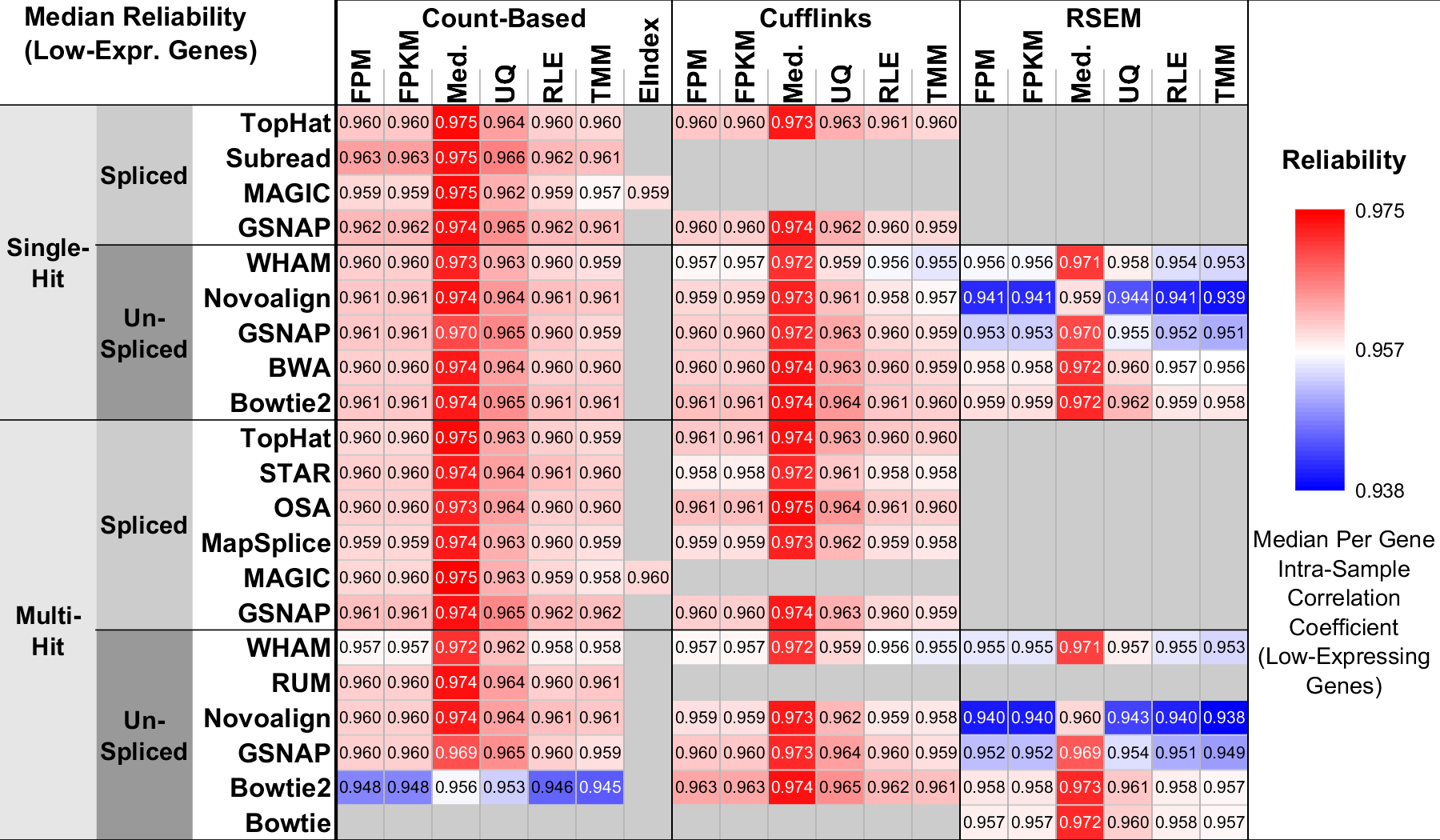
**

# Supplementary Figure 6: Median Reliability of Low-Expressing Genes.

The 278 RNA-seq pipelines applied to the SEQC-benchmark dataset differ in the median reliability of low-expressing genes. Reliability is defined as the intraclass (or intra-sample in our case) correlation that quantifies how similar replicate libraries of a sample are to one another using analysis of variance techniques. It is encoded as color, with red representing the highest reliability, or the highest intraclass correlation. “Low-Expressing Genes” refers to the 2,044 qPCR genes after filtering.

**
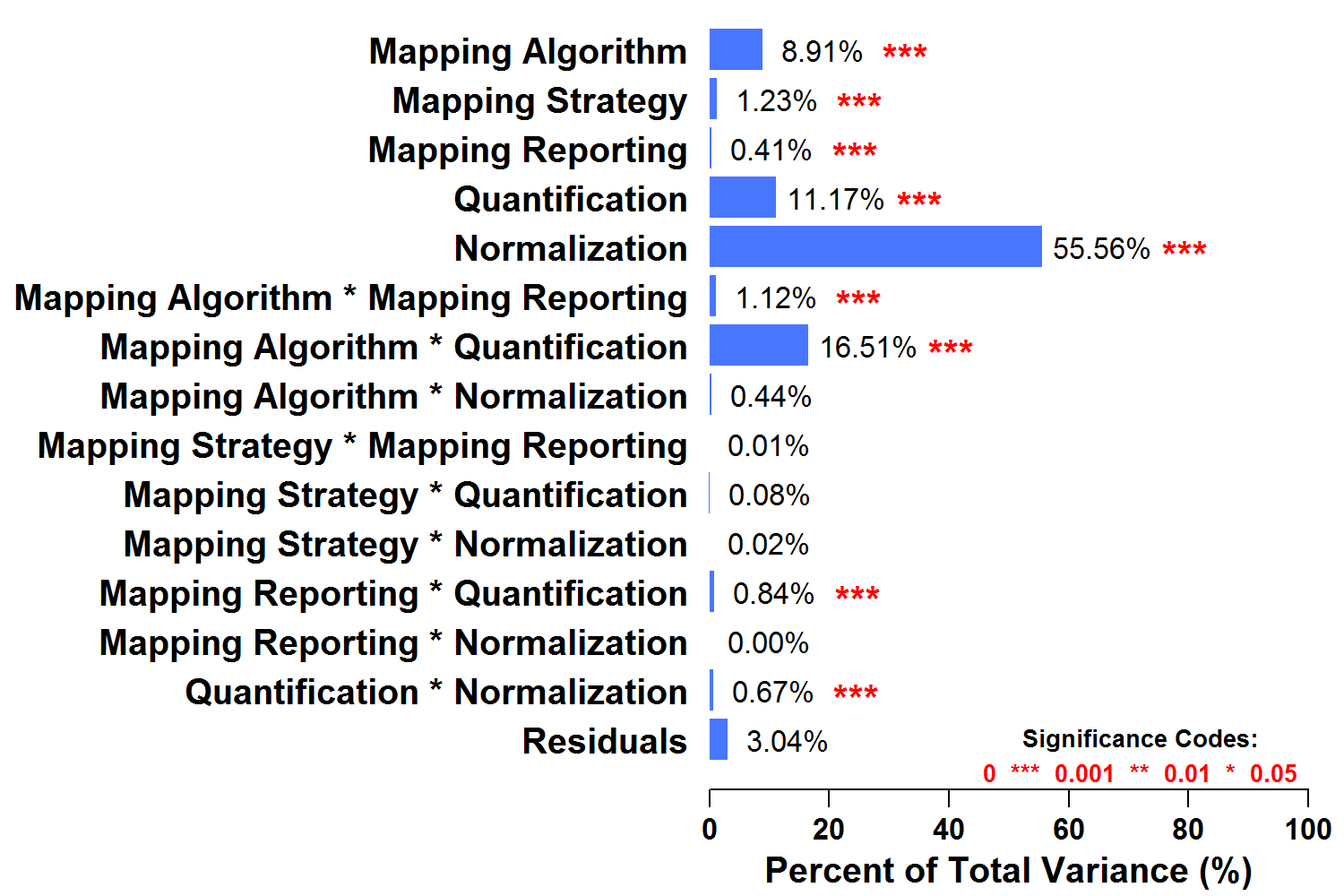
**

# Supplementary Figure 7: ANOVA for the Median Reliability of Low-Expressing Genes.

Analysis of variance (ANOVA) decomposes the overall variance in the median reliability of low-expressing genes into various factors considered, including RNA-seq pipeline components and associated two-way interactions. The statistical significance of the contribution of each component and interaction is denoted by red asterisks, with ‘***’ indicates p-values are smaller than 0.001, ‘**’ indicates p-values are smaller than 0.01, and ‘*’ indicates p-values are smaller than 0.05. Among all components and interactions, the normalization contributes the most to the overall variance.


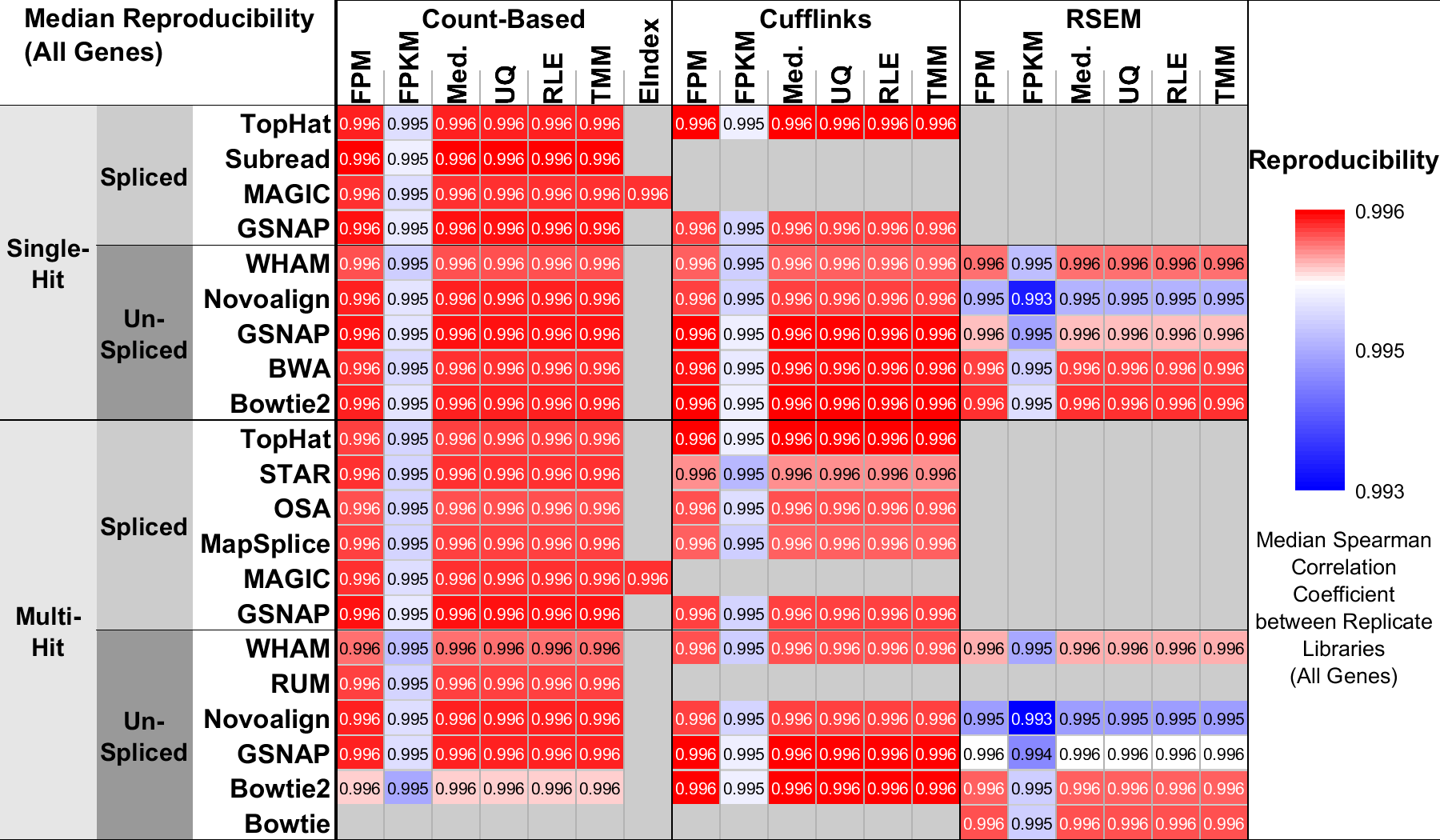


# Supplementary Figure 8: Median Reproducibility of All Genes.

Reproducibility is defined as the Spearman correlation between two replicate libraries of the same sample. It is encoded as color, with red representing the highest reproducibility, or the highest Spearman correlation. “All Genes” refers to the 10,222 qPCR genes after filtering.


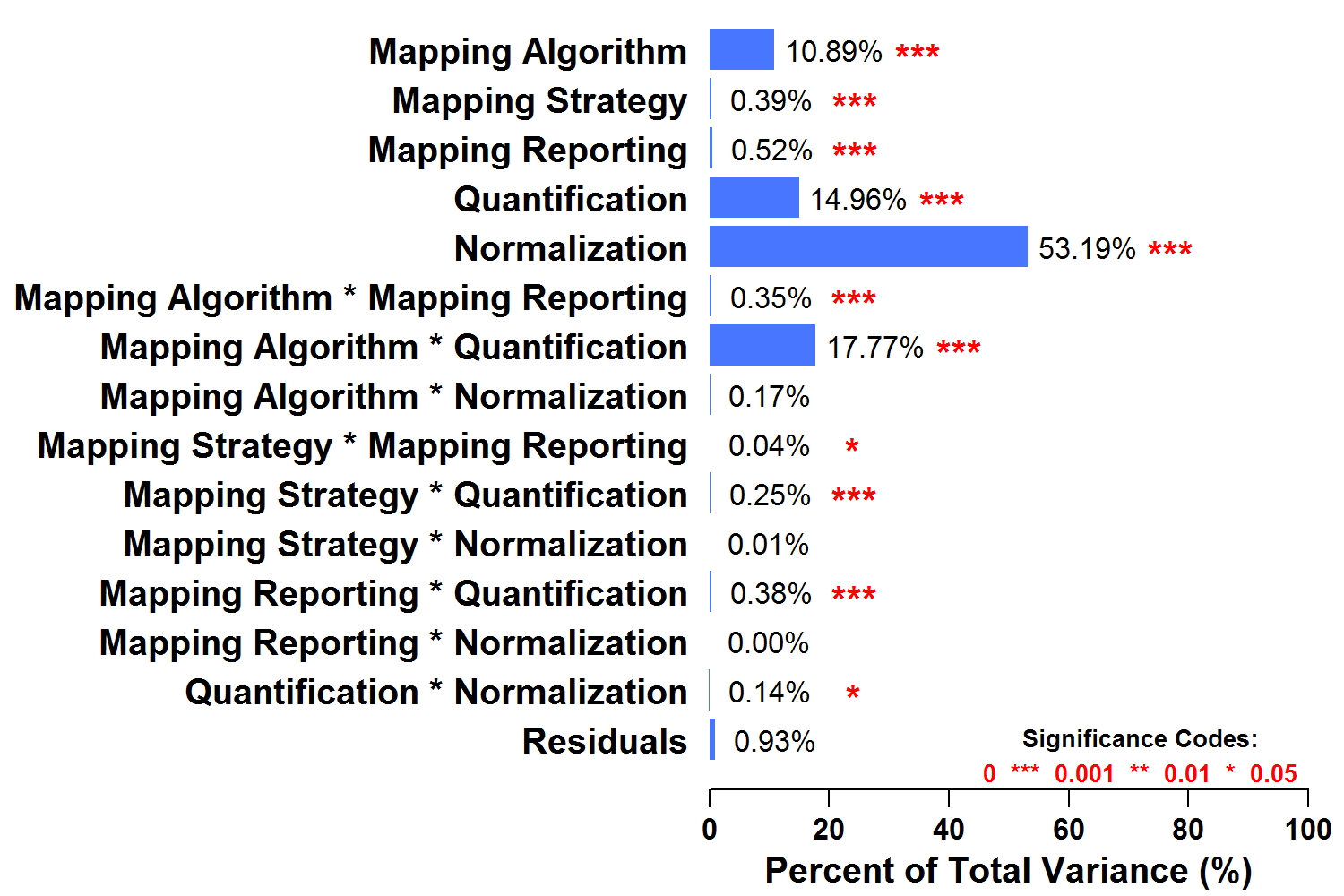


# Supplementary Figure 9: ANOVA for the Median Reproducibility of All Genes.

Analysis of variance (ANOVA) decomposes the overall variance in the median reproducibility of all genes into various factors considered, including RNA-seq pipeline components (i.e., mapping algorithm, mapping strategy, mapping reporting, quantification, and normalization) and associated two-way interactions. The statistical significance of the contribution of each component and interaction is denoted by red asterisks, with ‘***’ indicates p-values are smaller than 0.001, ‘**’ indicates p-values are smaller than 0.01, and ‘*’ indicates p-values are smaller than 0.05. Among all components and interactions, the normalization, quantification, mapping algorithm, and mapping algorithm-quantification interaction contribute the most to the overall variance.

**
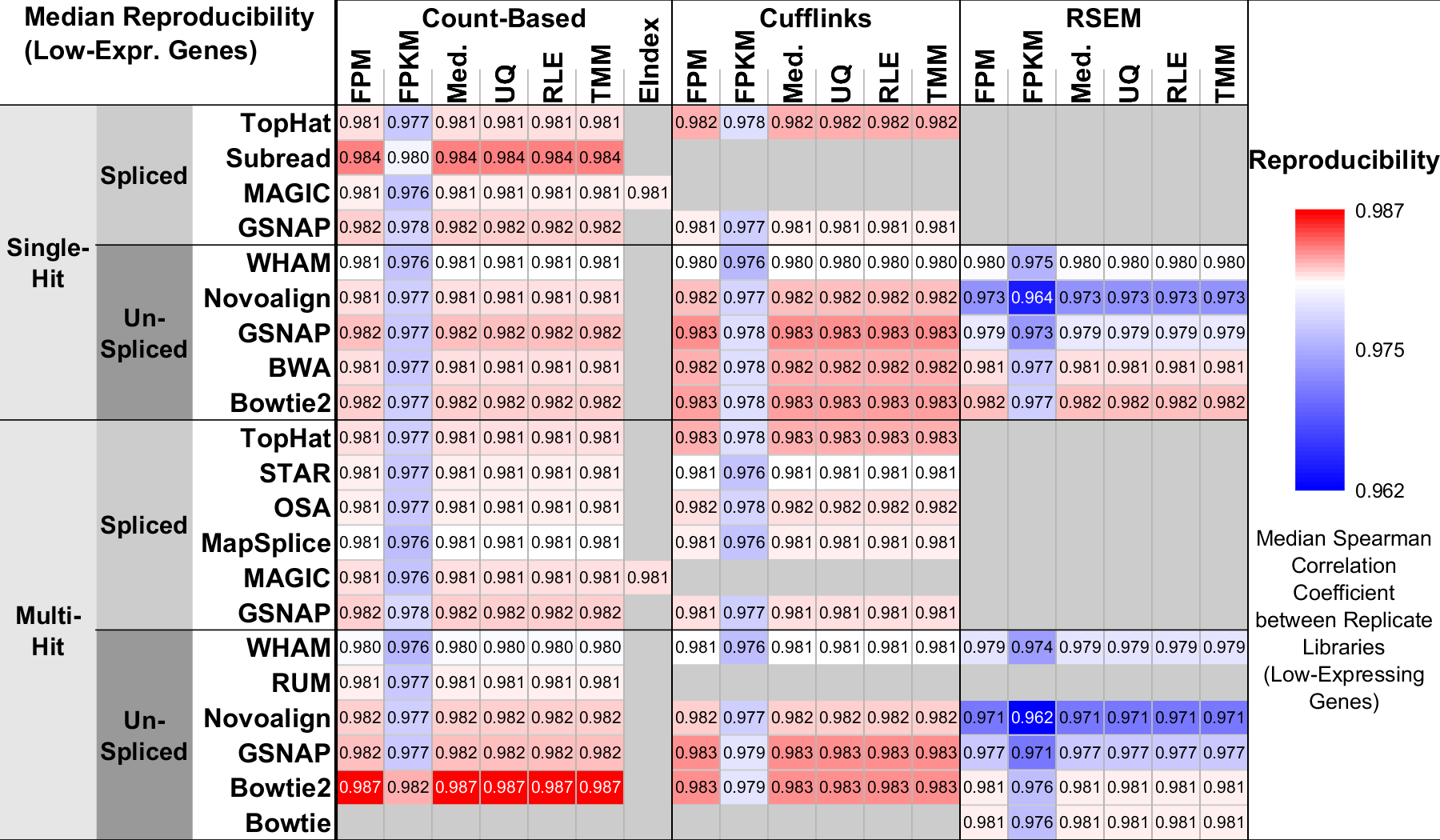
**

# Supplementary Figure 10: Median Reproducibility of Low-Expressing Genes.

The 278 RNA-seq pipelines applied to the SEQC-benchmark dataset differ in the median reproducibility of all genes. Reproducibility is defined as the Spearman correlation between two replicate libraries of the same sample. It is encoded as color, with red representing the highest reproducibility, or the highest Spearman correlation. “Low-Expressing Genes” refers to the 2,044 qPCR genes after filtering.

**
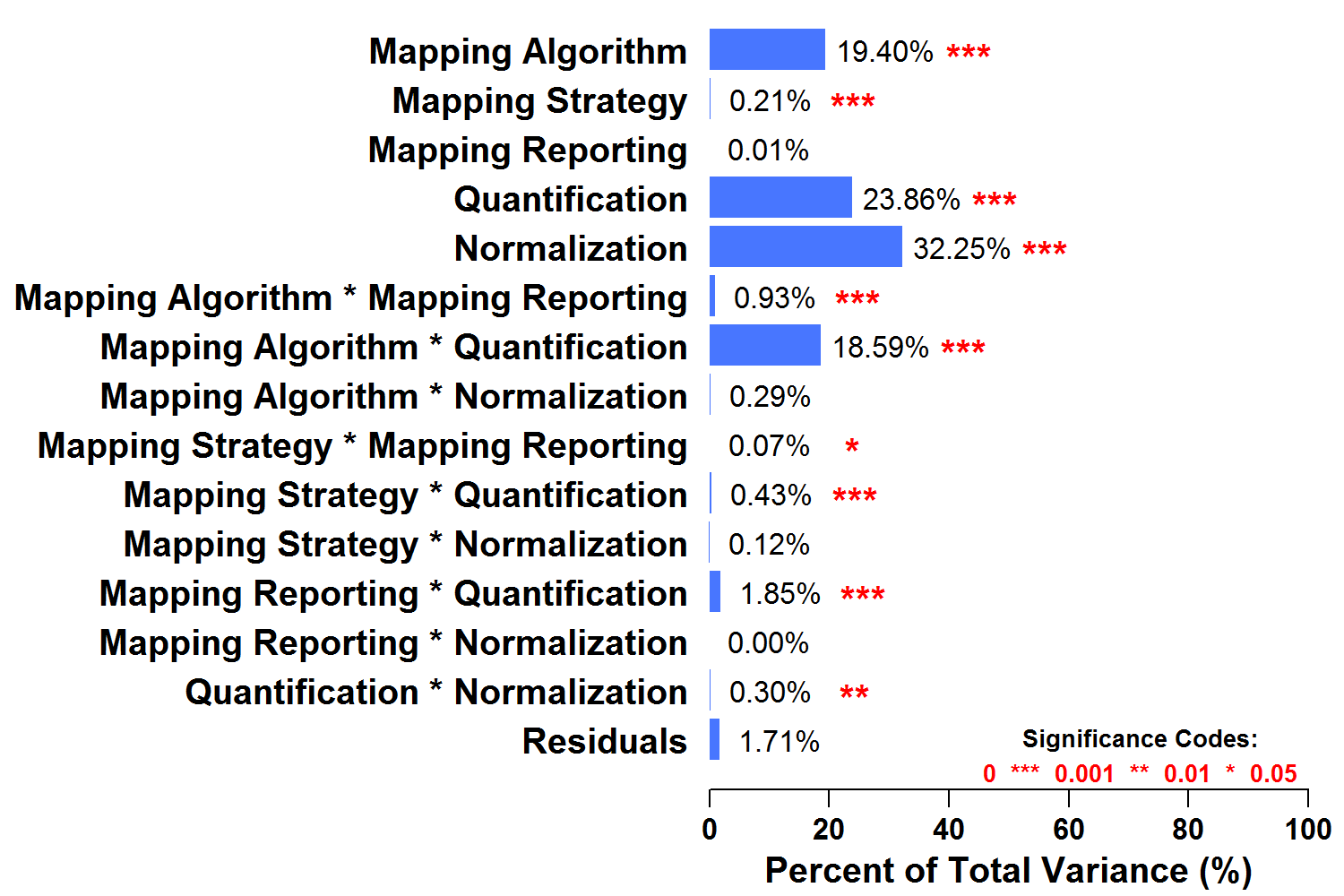
**

# Supplementary Figure 11: ANOVA for the Median Reproducibility of Low-Expressing Genes.

Analysis of variance (ANOVA) decomposes the overall variance in the median reproducibility of low-expressing genes into various factors considered, including RNA-seq pipeline components and associated two-way interactions. The statistical significance of the contribution of each component and interaction is denoted by red asterisks, with ‘***’ indicates p-values are smaller than 0.001, ‘**’ indicates p-values are smaller than 0.01, and ‘*’ indicates p-values are smaller than 0.05. Among all components and interactions, the normalization, quantification, mapping algorithm, and mapping algorithm-quantification interaction contribute the most to the overall variance.


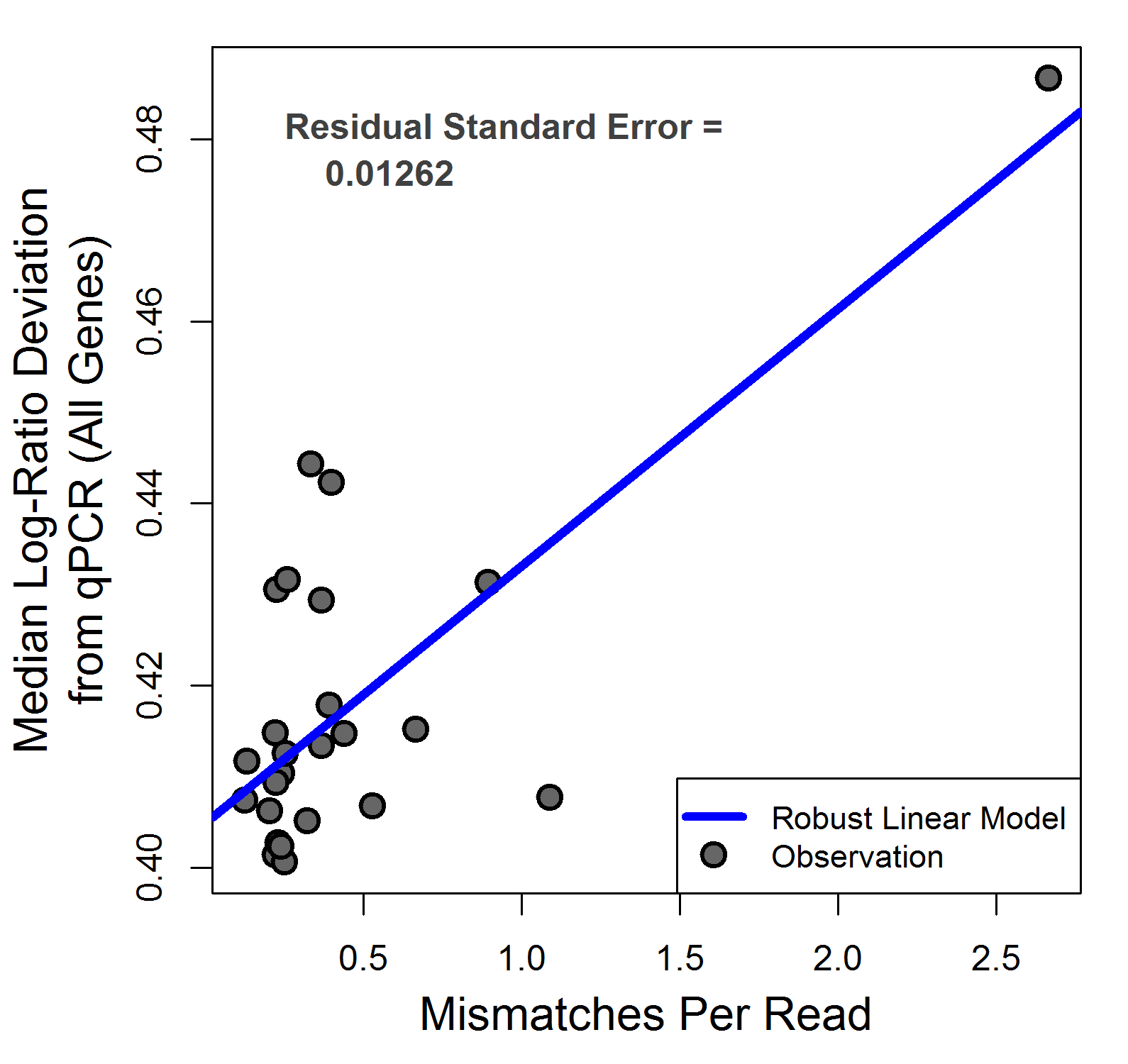

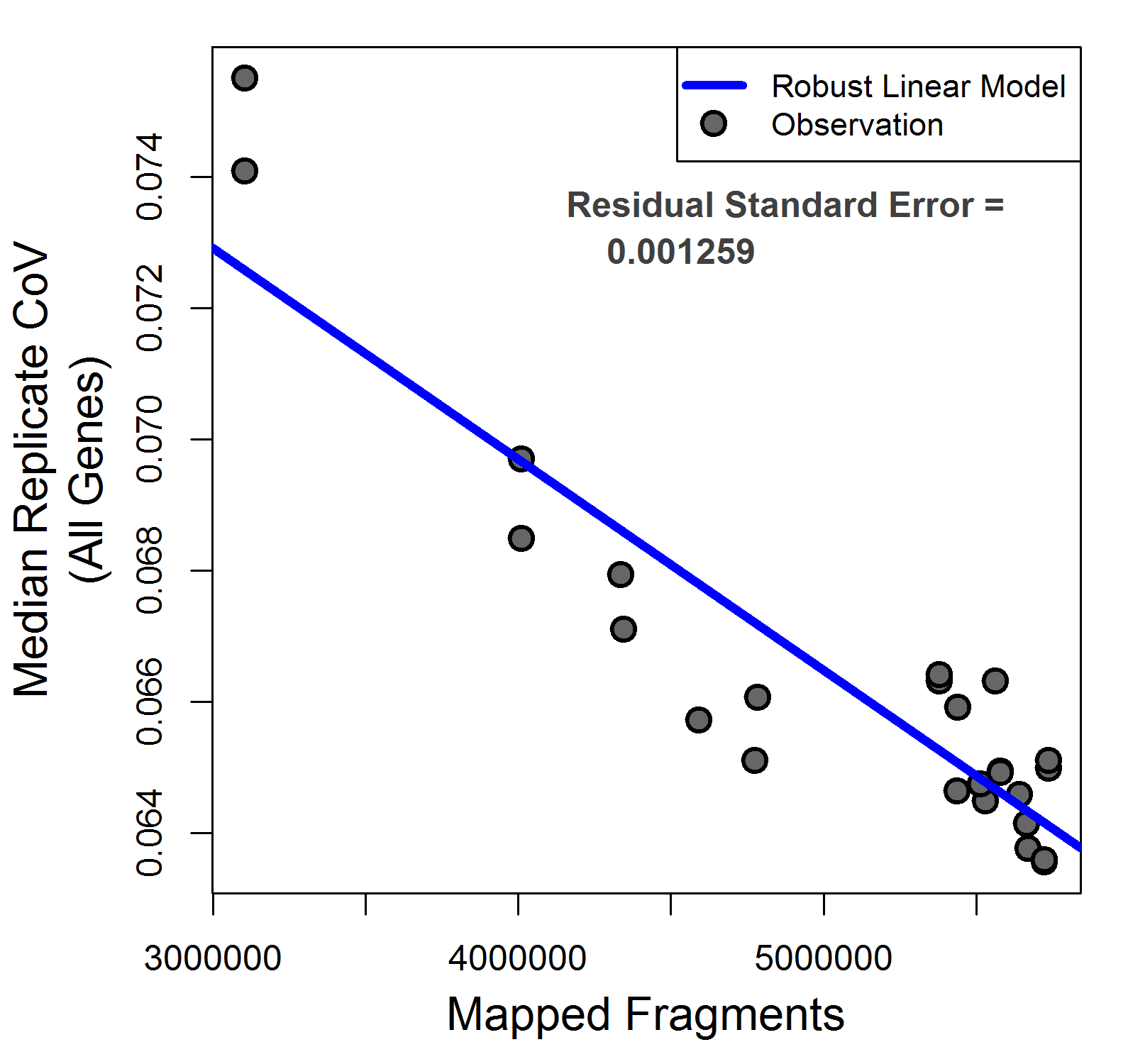

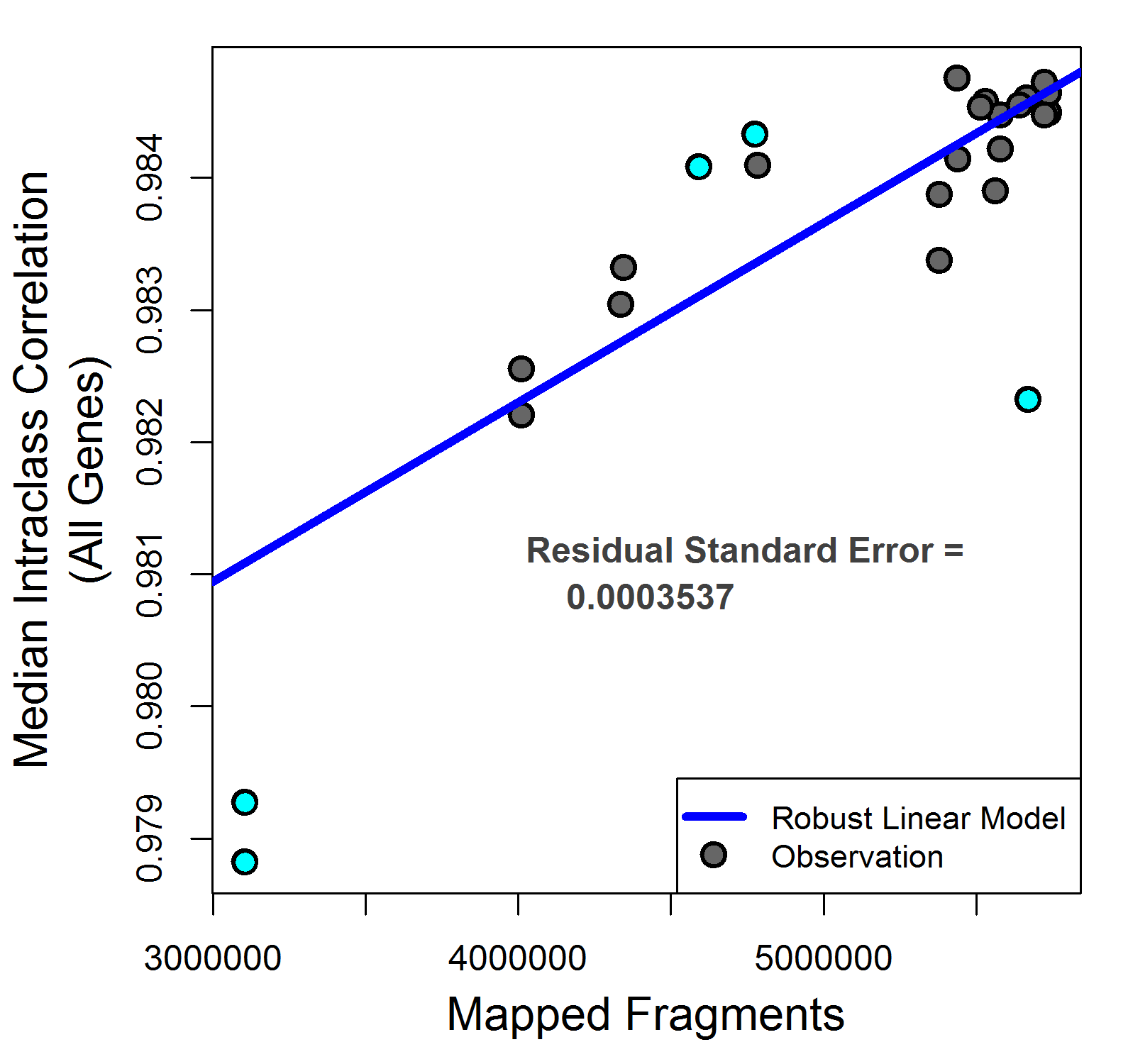

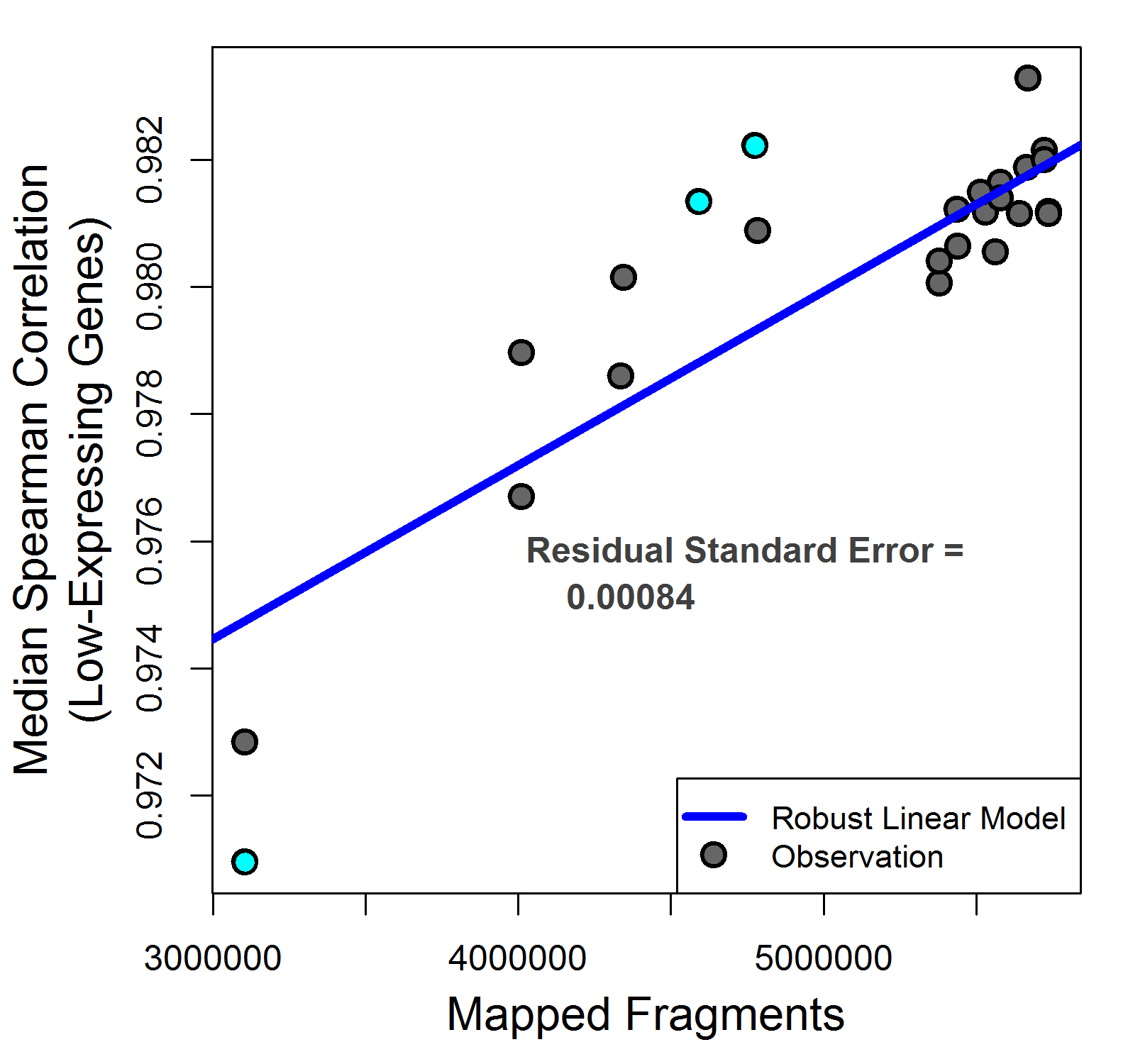


**d**

**c**

**b**

**a**

# Supplementary Figure 12: Relationship between Alignment Profiles and Benchmark Metrics.

The performance of benchmark metrics correlates with alignment profiles. For each panel, the x-axis represents an alignment profile, and the y-axis corresponds to a benchmark metric. Each gray point represents a sequence mapping pipeline, and the blue line depicts the robust linear model using M-estimation with Huber weighting (see online methods for more details). Points in cyan have Huber weights less than 0.5 (i.e., potential outlying points). (a) The median deviation of all genes positively correlates with the number of mismatches per read. More mismatches per read results in higher deviation, or lower accuracy with our metric definition; (b) the median coefficient of variation (CoV) of all genes negatively correlates with the number of mapped fragments. More mapped fragments results in lower CoV, or higher precision with our metric definition; (c) the median intraclass correlation of all genes positively correlates with the number of mapped fragments. More mapped fragments results in higher intraclass correlation, or higher reliability with our metric definition; and finally (d) the median Spearman correlation of low-expressing genes positively correlates with the number of mapped fragments. More mapped fragments results in higher Spearman correlation, or higher reproducibility with our metric definition.

**
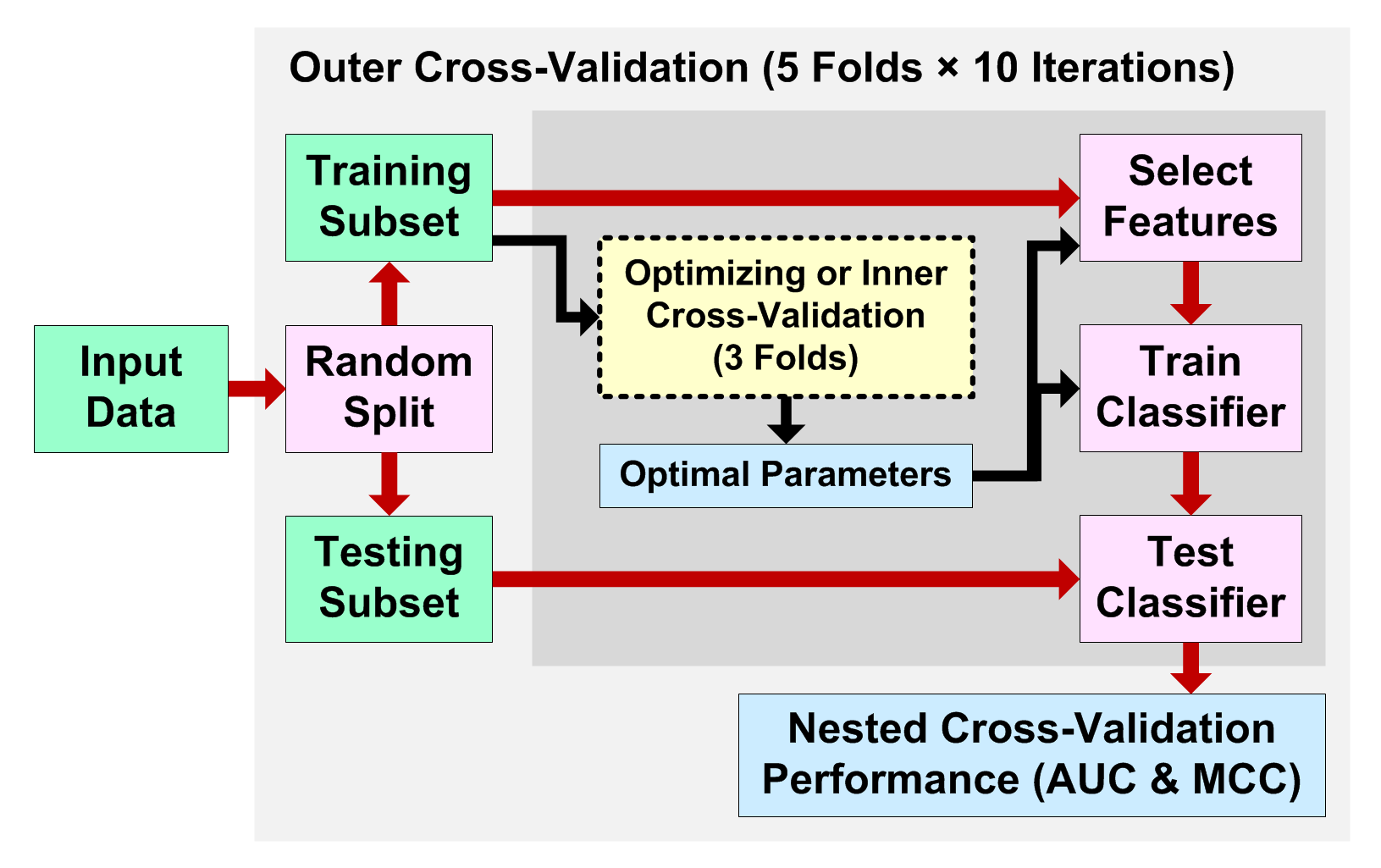
**

# Supplementary Figure 13: Predictive Modeling Procedure Using Nested Cross-Validation.

For the outer cross-validation, input data are randomly split into training and testing subsets (green boxes) following the standard 5-fold cross-validation protocol. For each of the five training subsets, the 3-fold optimizing or inner cross-validation (yellow boxes) is applied to optimize the feature size and hyperparameters for classifiers. The optimal feature size and hyperparameters (blue boxes) are used to train a final classifier (pink boxes) that will be directly applied to the testing subset. The final predictive modeling performance is measured by both the area under the receiver operating characteristic curve (AUROC, or simply AUC) and the Matthews correlation coefficient (MCC).

**a**

**
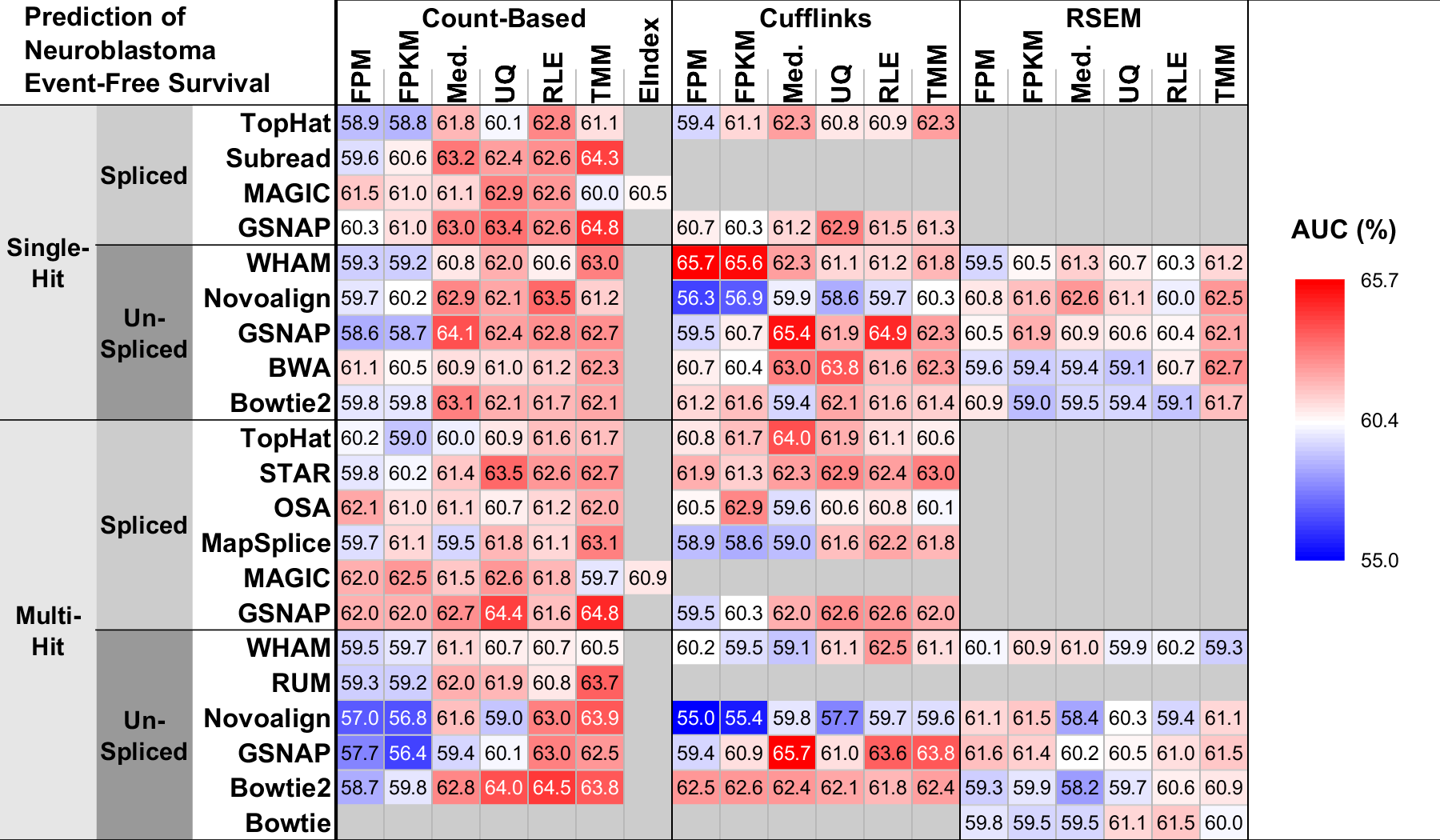
**

**b**

**
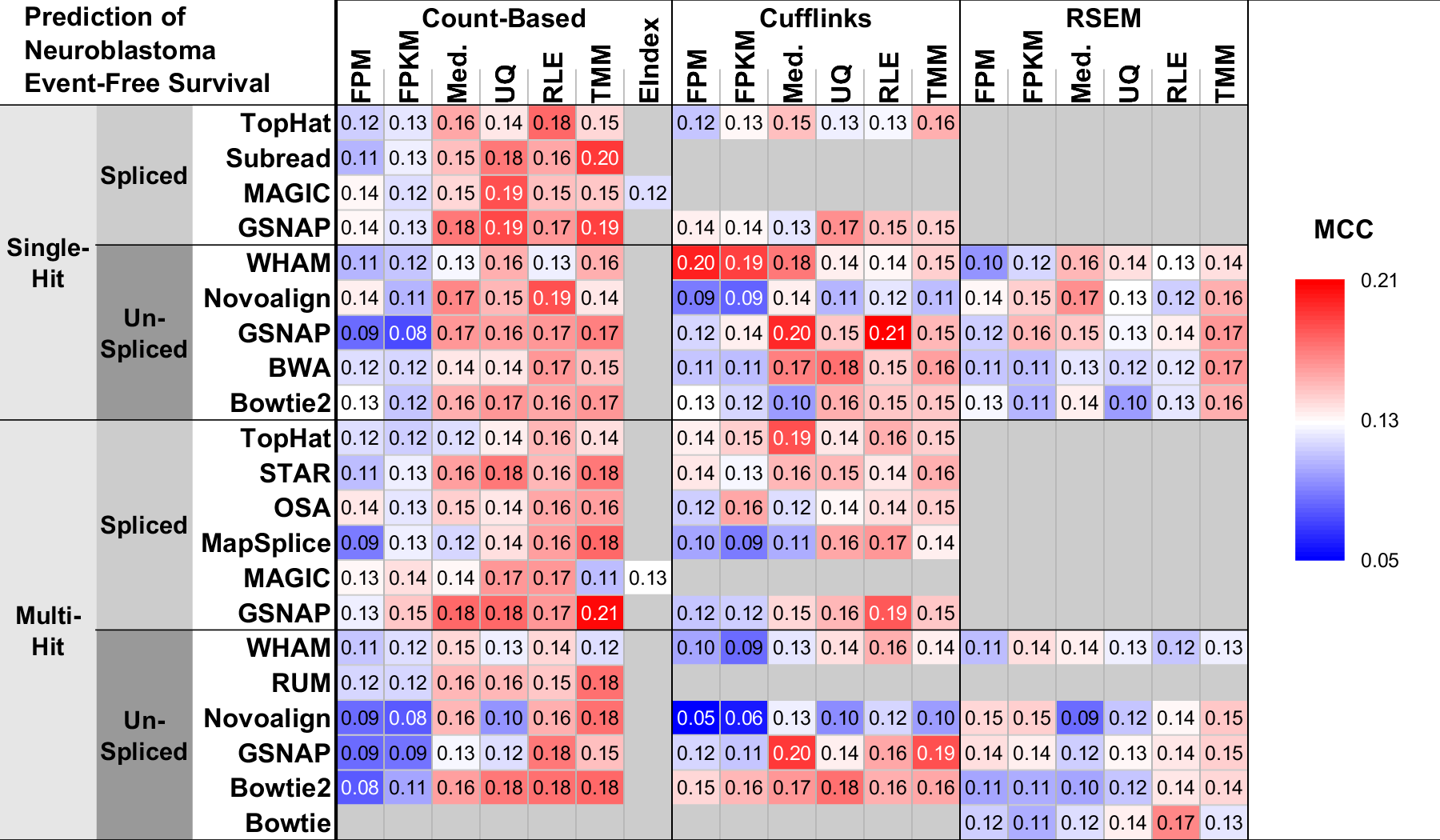
**

# Supplementary Figure 14: Prediction Performance of NB EFS Measured by AUC and MCC.

The 278 RNA-seq pipelines applied to the SEQC-neuroblastoma (NB) dataset differ in terms of prediction performance measured by (a) AUC and (b) MCC. The predictive modeling procedure is detailed in **Supplementary Figure 13**, and the prediction endpoint is dichotomized event-free survival (EFS) with the survival-time threshold of two years. Prediction performance is encoded as color, with red representing the highest AUC or MCC.

**a**

**
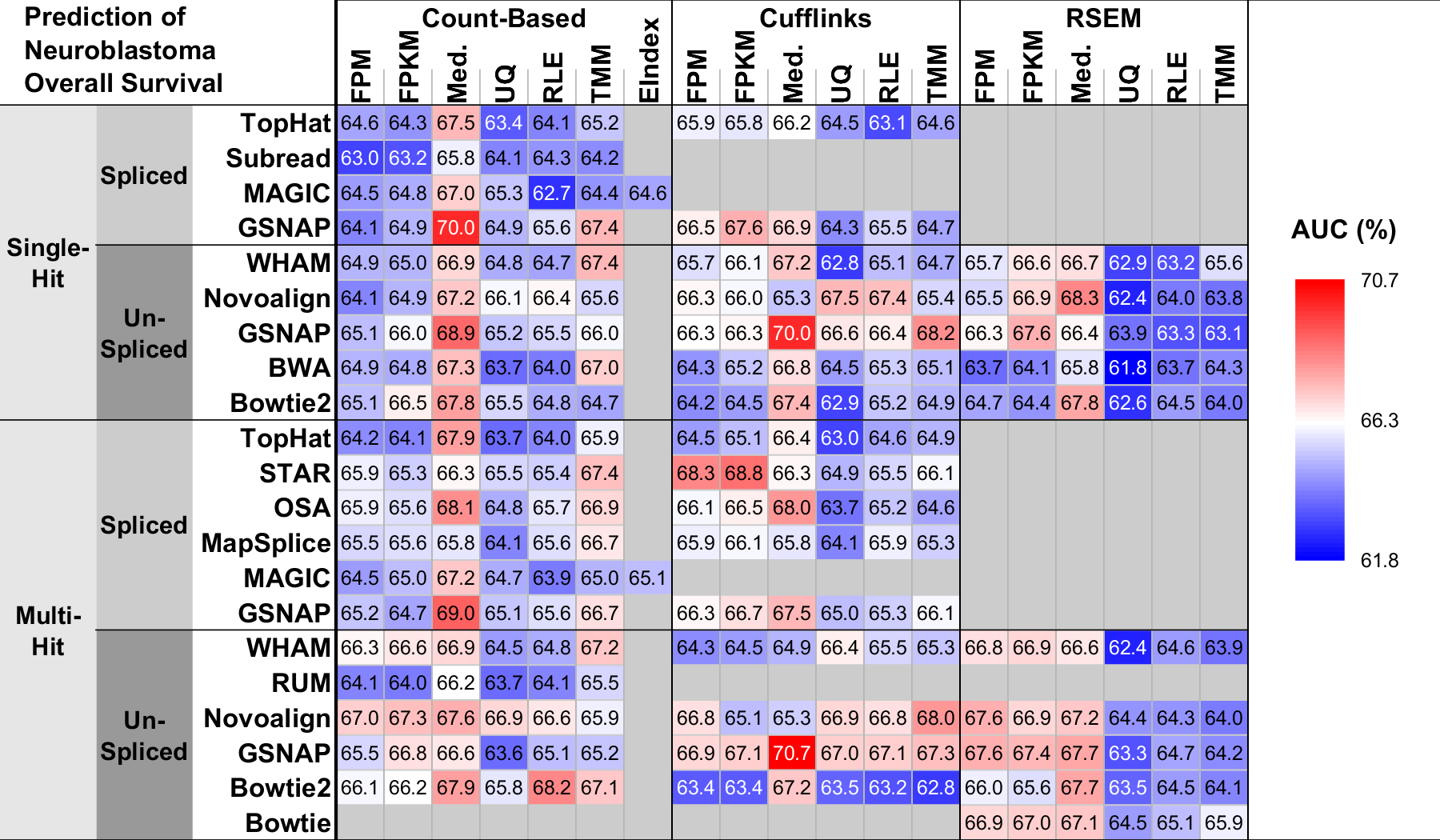
**

**b**

**
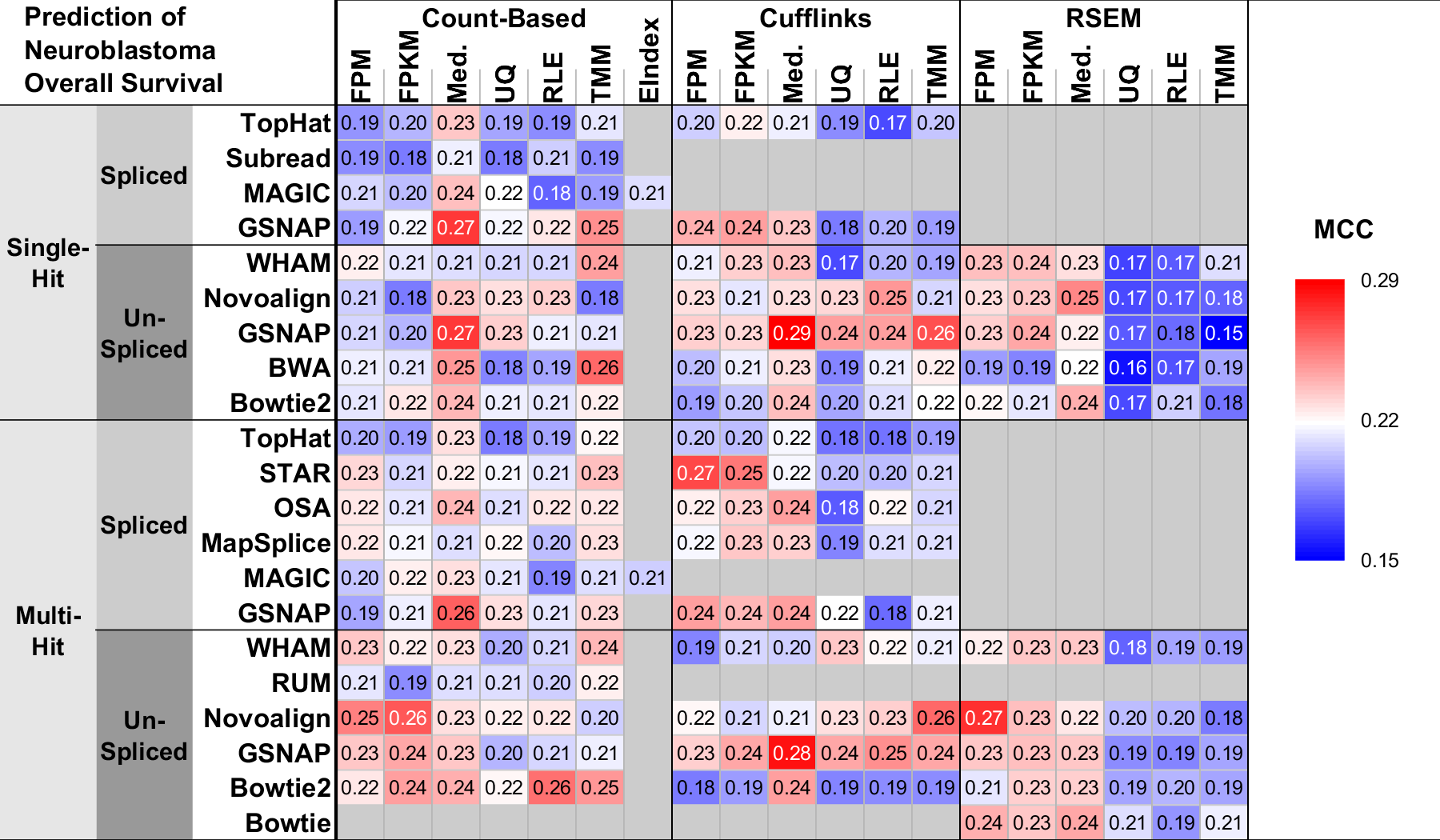
**

# Supplementary Figure 15: Prediction Performance of NB OS Measured by AUC and MCC.

The 278 RNA-seq pipelines applied to the SEQC-neuroblastoma (NB) dataset differ in terms of prediction performance measured by (a) AUC and (b) MCC. The predictive modeling procedure is detailed in **Supplementary Figure 13**, and the prediction endpoint is dichotomized overall survival (OS) with the survival-time threshold of three years. Prediction performance is encoded as color, with red representing the highest AUC or MCC.

**a**

**
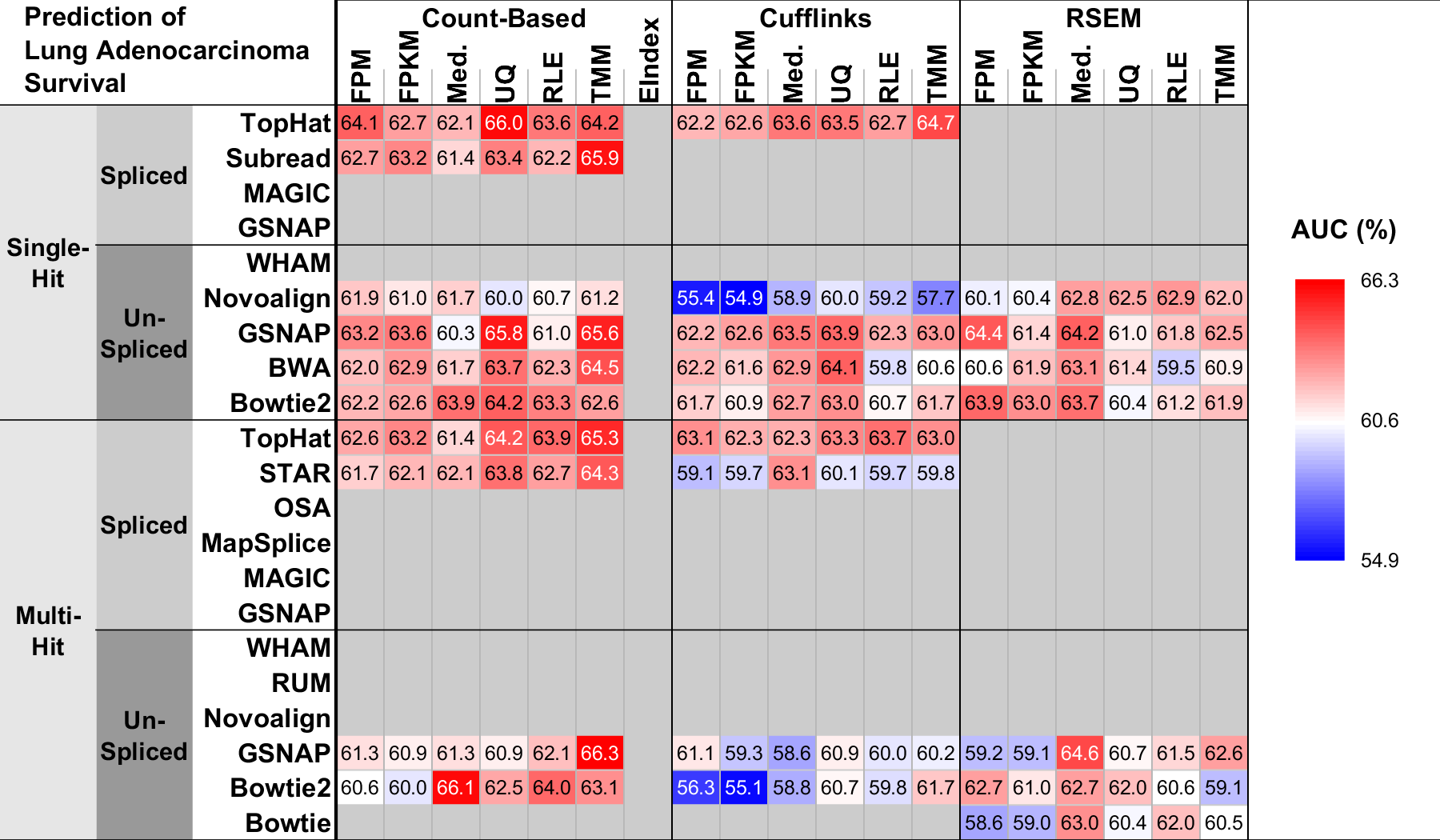
**

**b**

**
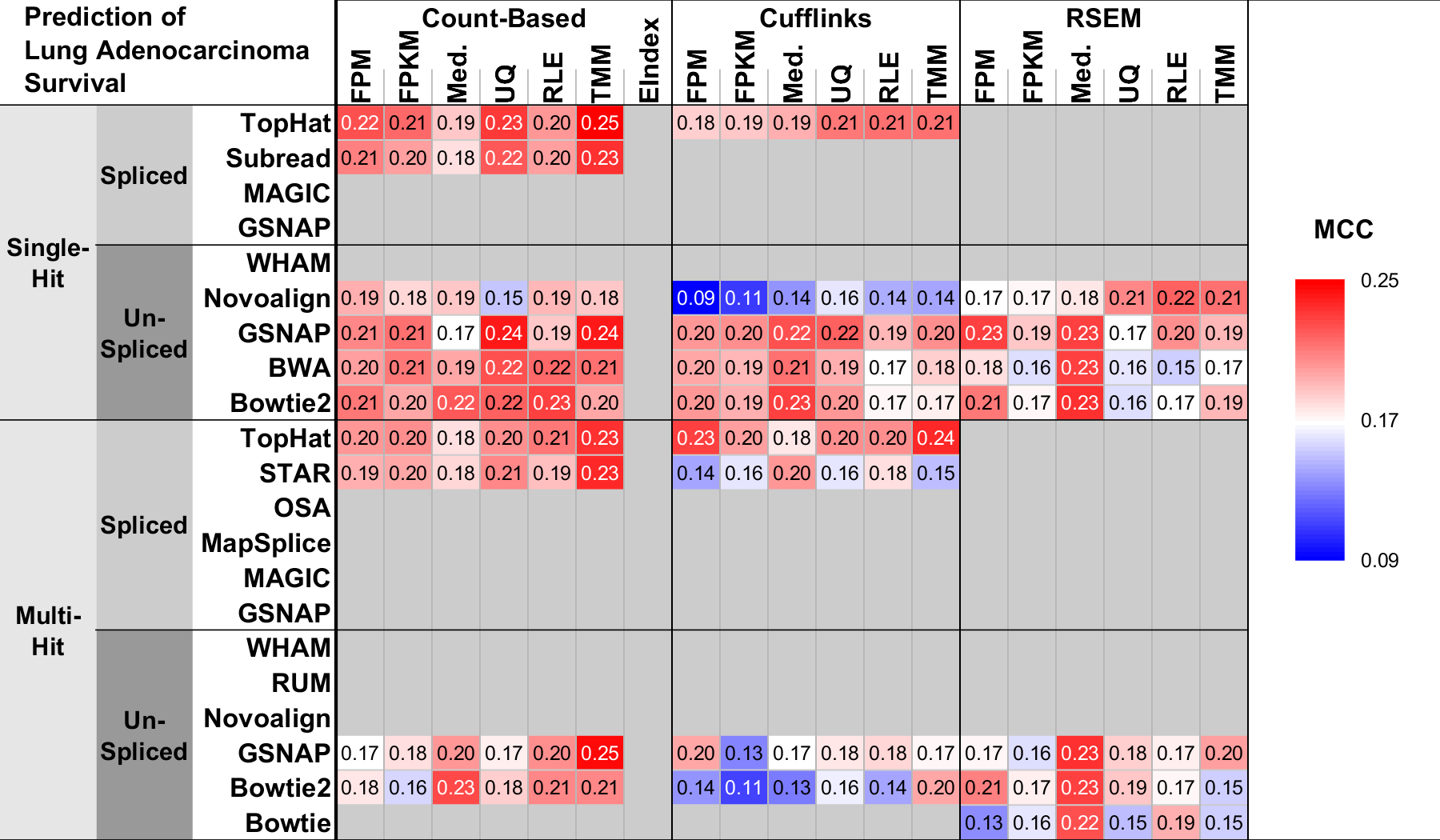
**

# Supplementary Figure 16: Prediction Performance of LUAD Survival Measured by AUC and MCC.

The 156 RNA-seq pipelines applied to the TCGA-lung-adenocarcinoma (LUAD) dataset differ in terms of prediction performance measured by (a) AUC and (b) MCC. The predictive modeling procedure is detailed in **Supplementary Figure 13**, and the prediction endpoint is dichotomized survival with the survival threshold of two years. Prediction performance is encoded as color, with red representing the highest AUC or MCC.

**a Prediction
Performance
of NB EFS (AUC)**

**
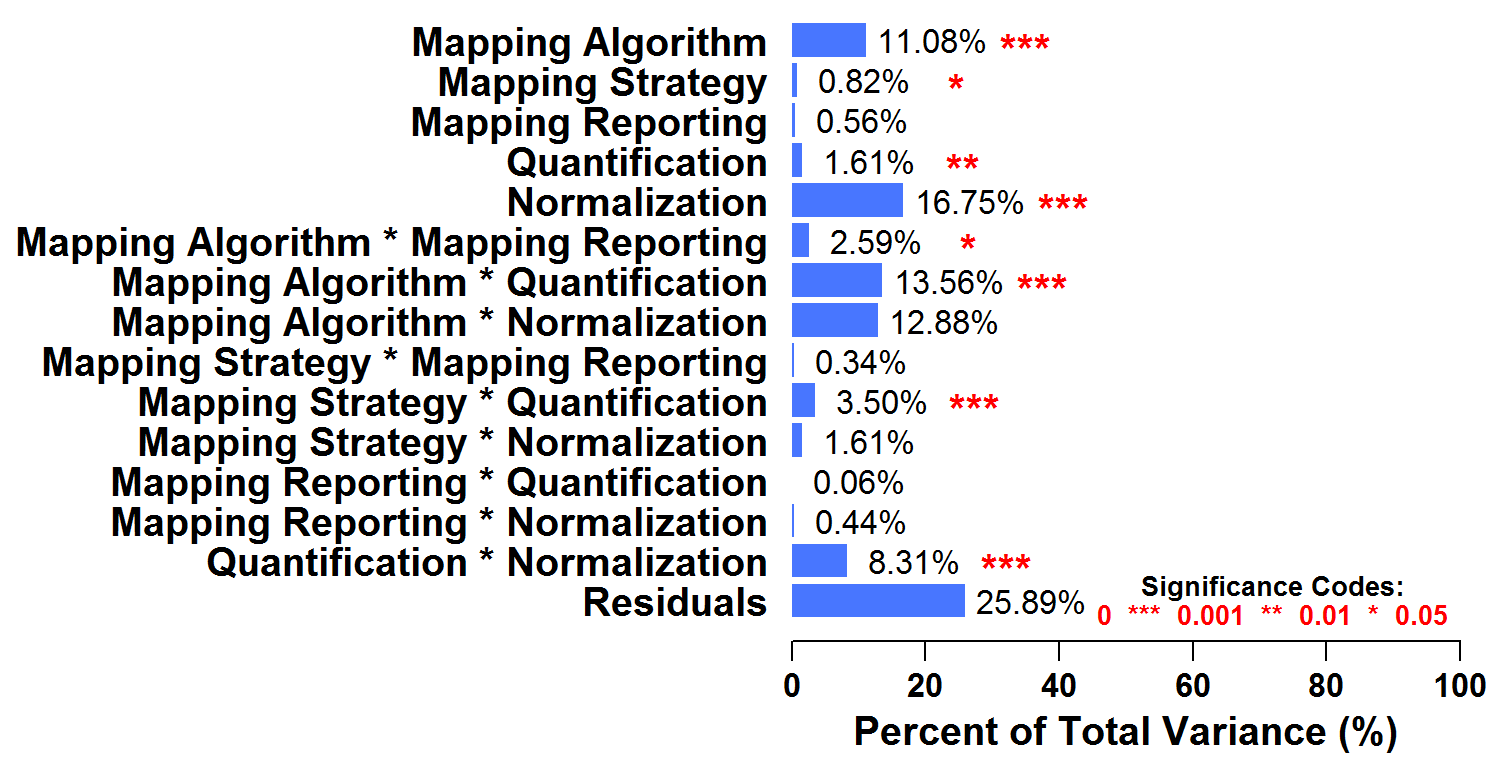
**

**
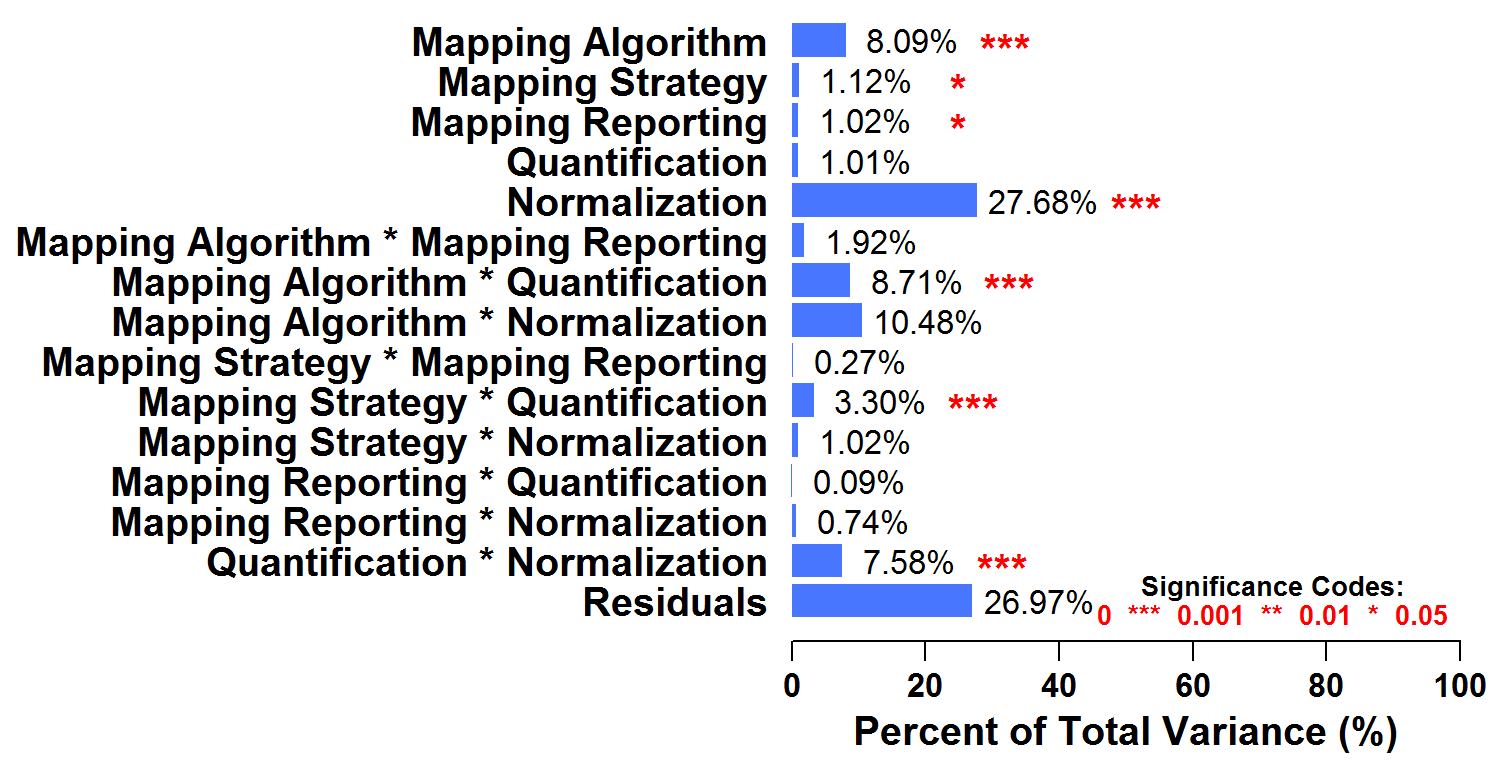
**

**b Prediction
 Performance
 of NB EFS (MCC)**

# Supplementary Figure 17: ANOVA for Prediction Performance of NB EFS.

Analysis of variance (ANOVA) decomposes the overall variance in prediction performance of NB EFS into various factors considered, including RNA-seq pipeline components and associated two-way interactions. Panels (a) and (b) show the ANOVA for prediction AUC and MCC, respectively. Prediction performance of various classifiers has been averaged before applying the ANOVA. The statistical significance of the contribution of each component and interaction is denoted by red asterisks, with ‘***’ indicates p-values are smaller than 0.001, ‘**’ indicates p-values are smaller than 0.01, and ‘*’ indicates p-values are smaller than 0.05. Among all components and interactions, the normalization contributes the most to the overall variance. Around a quarter of the overall variance belongs to residuals that cannot be explained by the factors considered.

**a Prediction
Performance
of NB OS (AUC)**

**
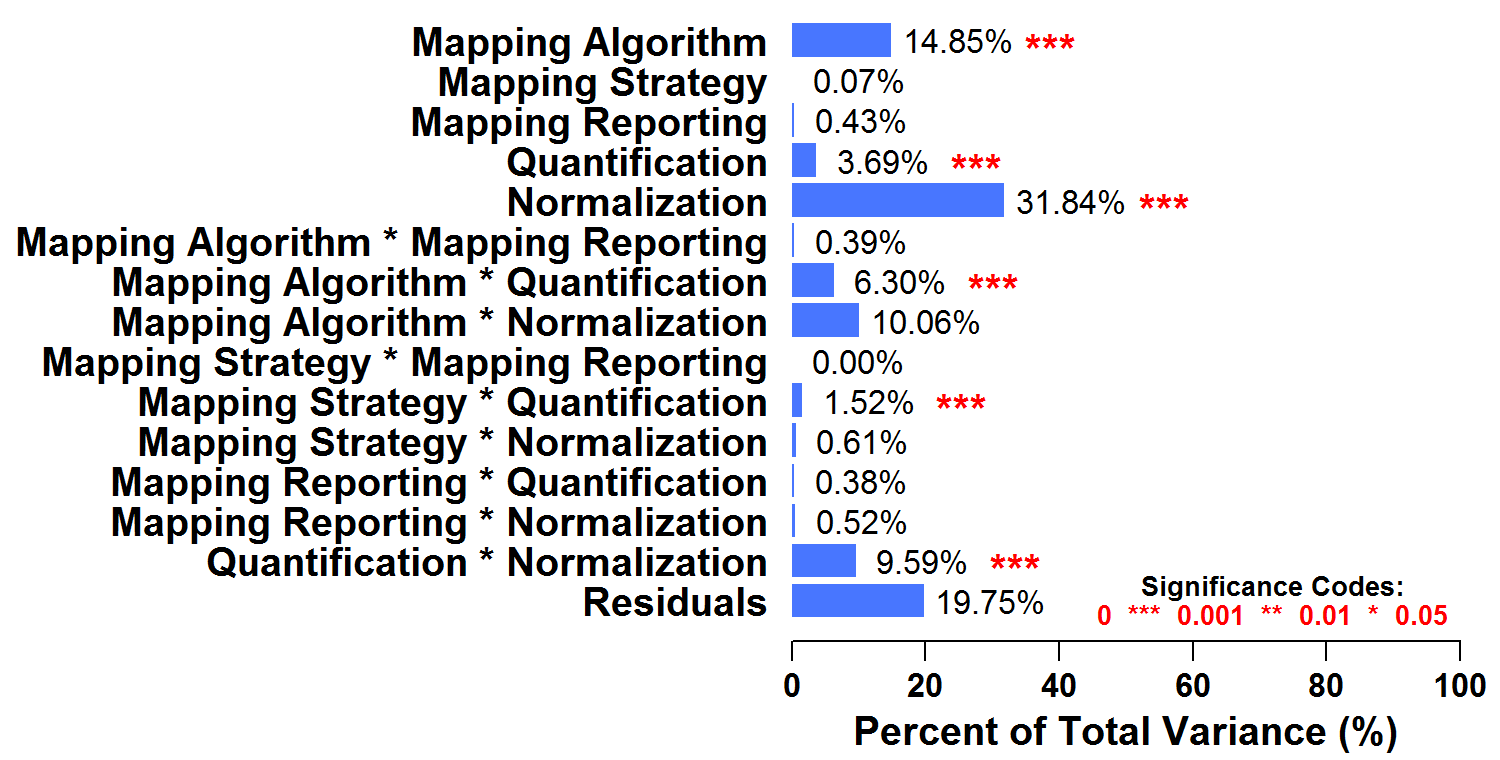
**

**
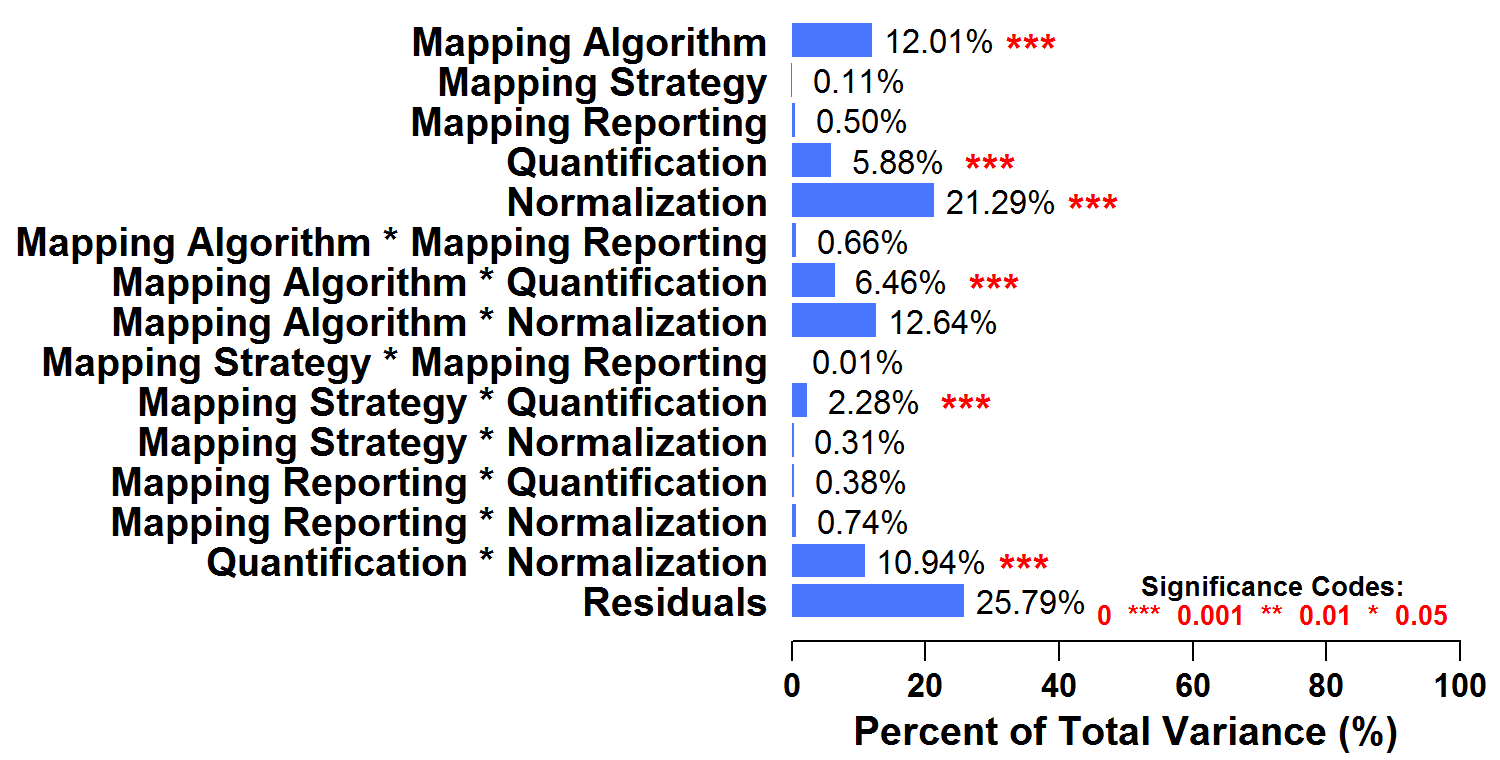
**

**b Prediction
 Performance
 of NB OS (MCC)**

# Supplementary Figure 18: ANOVA for Prediction Performance of NB OS.

Analysis of variance (ANOVA) decomposes the overall variance in prediction performance of NB OS into various factors considered, including RNA-seq pipeline components and associated two-way interactions. Panels (a) and (b) show the ANOVA for prediction AUC and MCC, respectively. Prediction performance of various classifiers has been averaged before applying the ANOVA. The statistical significance of the contribution of each component and interaction is denoted by red asterisks, with ‘***’ indicates p-values are smaller than 0.001, ‘**’ indicates p-values are smaller than 0.01, and ‘*’ indicates p-values are smaller than 0.05. Among all components and interactions, the normalization contributes the most to the overall variance. Around a quarter of the overall variance belongs to residuals that cannot be explained by the factors considered.

**a Prediction
Performance of
LUAD Survival (AUC)**

**
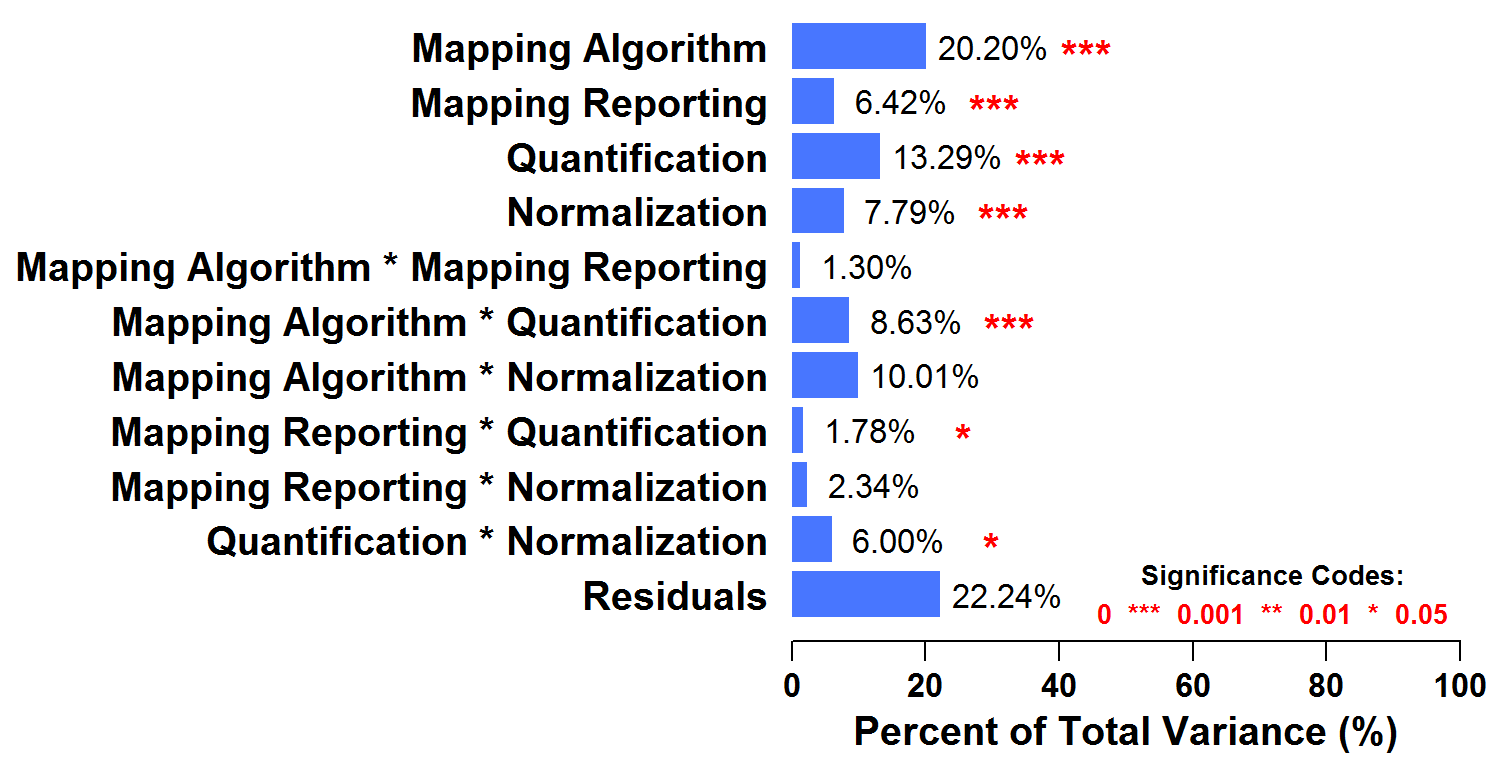
**

**
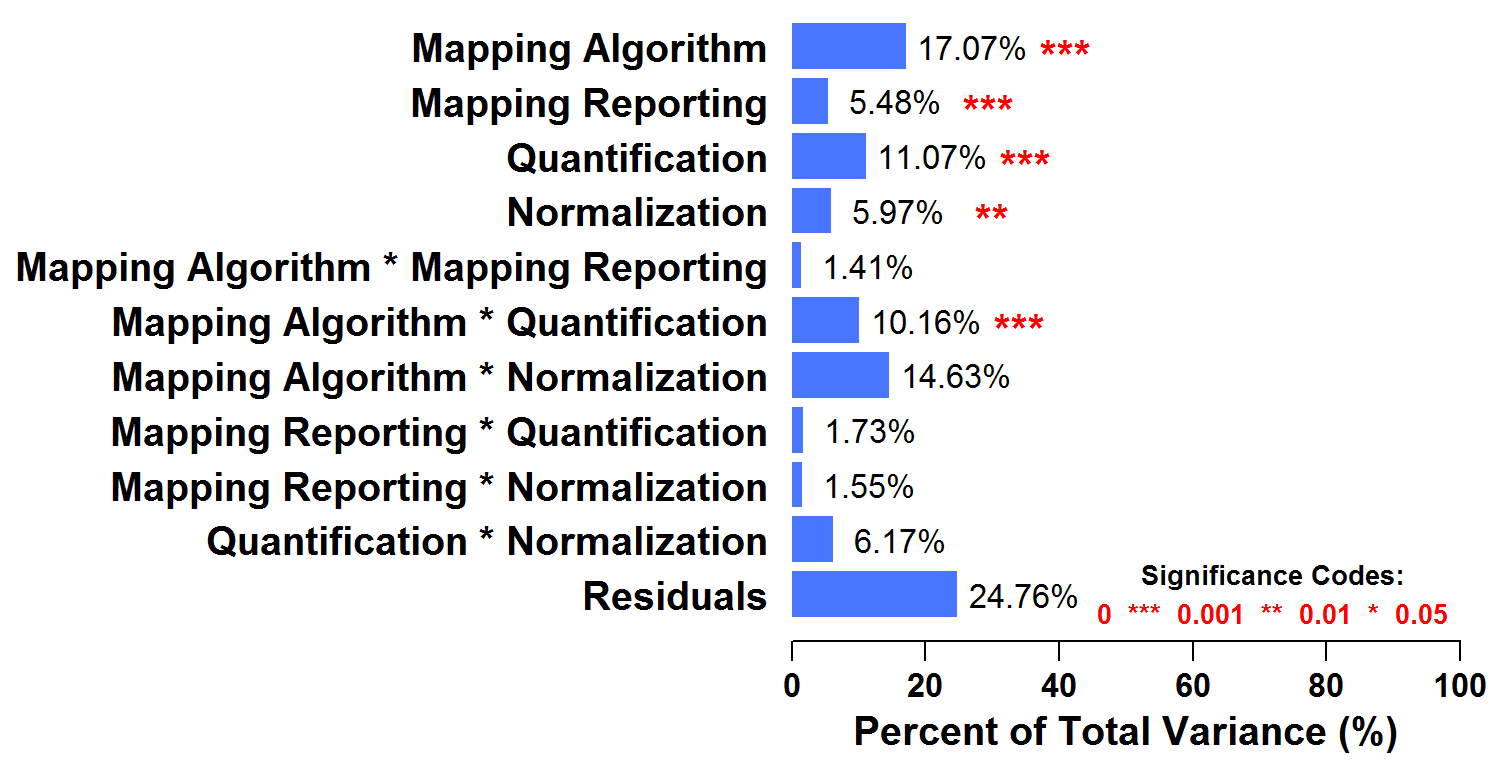
**

**b Prediction
Performance of
LUAD Survival (MCC)**

# Supplementary Figure 19: ANOVA for Prediction Performance of LUAD Survival.

Analysis of variance (ANOVA) decomposes the overall variance in prediction performance of LUAD survival into various factors considered, including RNA-seq pipeline components and associated two-way interactions. Panels (a) and (b) show the ANOVA for prediction AUC and MCC, respectively. Prediction performance of various classifiers has been averaged before applying the ANOVA. The statistical significance of the contribution of each component and interaction is denoted by red asterisks, with ‘***’ indicates p-values are smaller than 0.001, ‘**’ indicates p-values are smaller than 0.01, and ‘*’ indicates p-values are smaller than 0.05. Among all components and interactions, the mapping algorithm contributes the most to the overall variance. Note that more than a quarter of the overall variance belongs to residuals that cannot be explained by the factors considered.


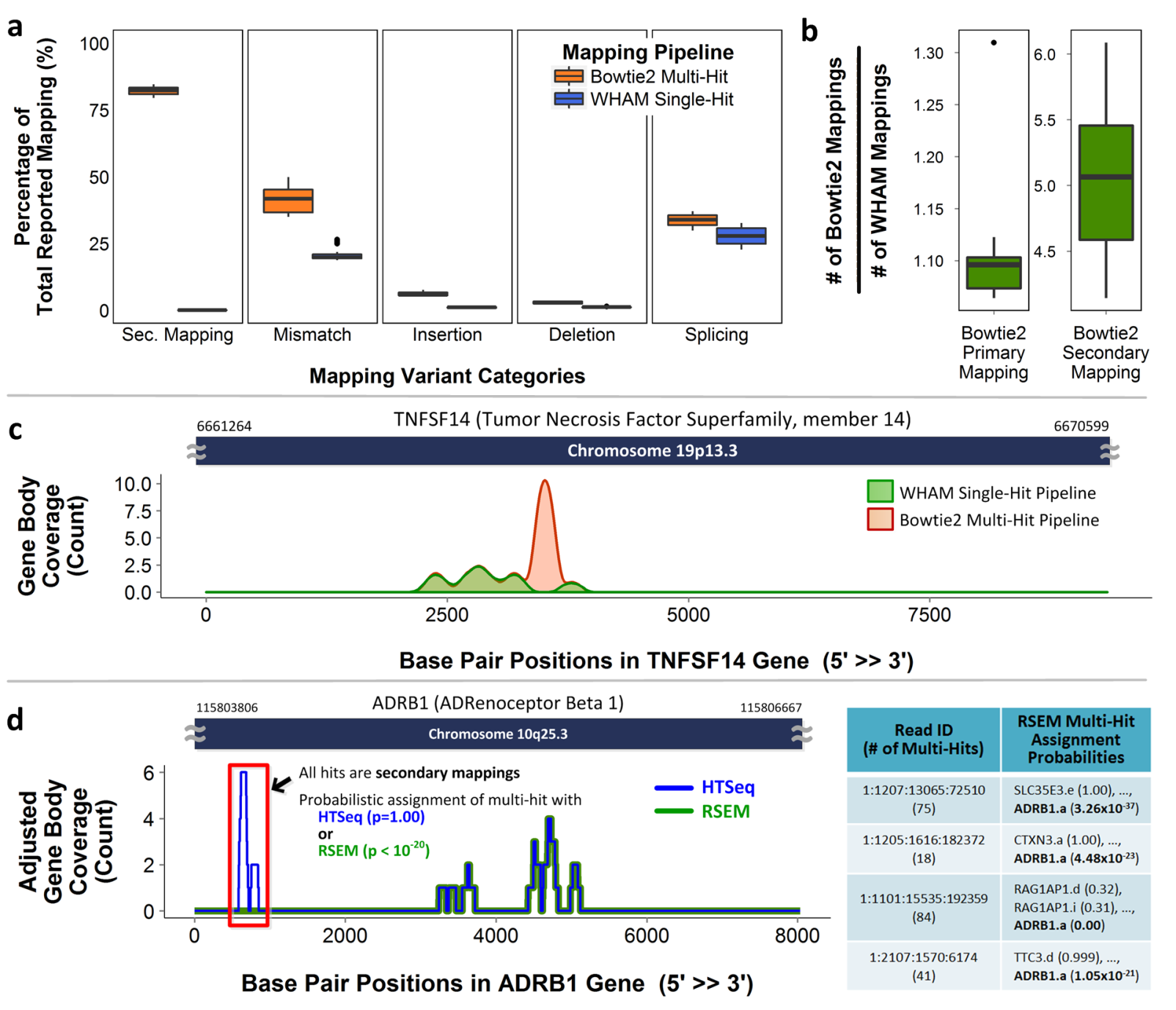


# Supplementary Figure 20: The Impact of RNA-seq Pipeline Choices on Sequence Mapping and Expression Quantification Outcome.

(a) Bowtie2 multi-hit and WHAM single-hit pipelines differ significantly in terms of the percentage of total reported mappings for each mapping variant (i.e., secondary mapping, mapping with mismatches, insertion, deletion, and splicing). Each box demonstrates the distribution of percentages calculated from multiple sample replicates in the SEQC-benchmark dataset. The Bowtie2 multi-hit pipeline reports a higher percentage of mapping variants than the WHAM single-hit pipeline. (b) The WHAM single-hit pipeline reports only primary mappings. The box plot shows the distribution of the ratios of total primary or secondary mappings of the Bowtie2 multi-hit pipeline to total primary mappings of the WHAM single-hit pipeline using multiple sample replicates in the SEQC-benchmark dataset. The Bowtie2 multi-hit pipeline reports slightly more primary mappings than the WHAM single-hit pipeline, and it reports approximately five times more secondary mappings than the WHAM single-hit pipeline. These additional secondary mappings are informative for some expression quantification algorithms. (c) Gene body coverage differs between the Bowtie2 multi-hit and WHAM single-hit pipelines. The former pipeline reports additional secondary mappings between position 3200 and 3800. (d) Gene body coverage adjusted by multi-hit assignment probabilities derived from the HTSeq or RSEM quantification pipeline.


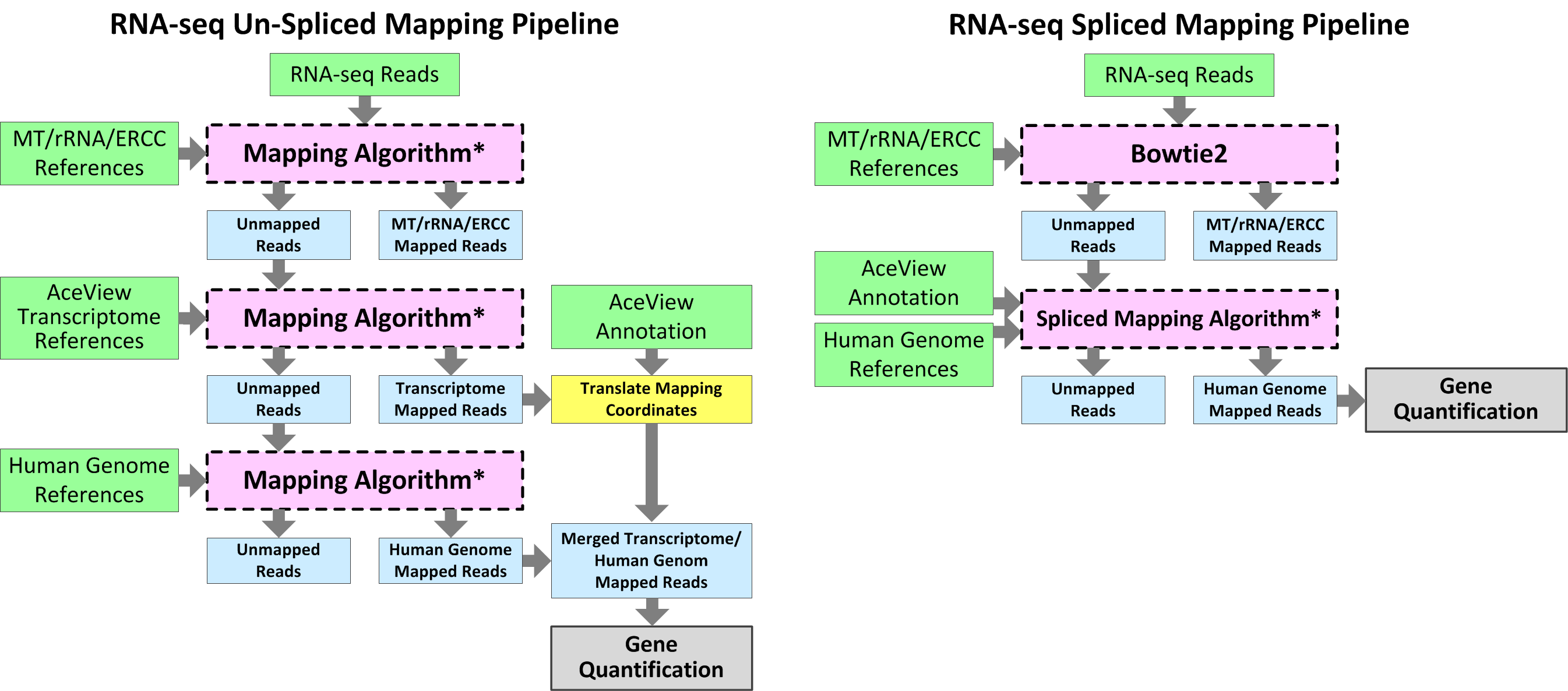


# Supplementary Figure 21: Un-spliced and Spliced RNA-seq Mapping Pipelines.

Both pipelines map to the MT/rRNA/ERCC (mitochondria, ribosomal RNA, and ERCC) reference first. The un-spliced mapping pipelines combine transcriptome and human genome mapping results into a single result for quantification (left). The spliced mapping pipelines either generate a single human genome mapping result or internally combine transcriptome and human genome mapping results into a single result for quantification (right). Thus, no additional merging step is required for spliced mapping pipelines.

# Supplementary Table 1: RNA-seq Pipelines.

The 278 RNA-seq pipelines include 13 mapping algorithms, three categories of quantification algorithms, and seven normalization methods.

| **RNA-seq Pipeline Factors** | | **# of Algorithm Choices** | **Algorithm Choices** |
| --- | --- | --- | --- |
| Mapping | Algorithm | 13 | Bowtie, Bowtie2, BWA, GSNAP, Magic, MapSplice, Novoalign, OSA, RUM, STAR, Subread, TopHat, WHAM |
|  | Strategy | 2 | Un-Spliced, Spliced |
|  | Reporting | 2 | Single-Hit, Multi-Hit |
| Quantification |  | 3 | Count-Based (i.e., HTSeq, and built-in quantifiers of Magic, RUM, and Subread), Cufflinks, RSEM |
| Normalization |  | 7 | FPM, FPKM, Median, Upper Quartile, RLE, TMM, Magic Expression Index |
| **Total Pipelines** | | **278*** |  |

*All combinations of mapping, quantification, and normalization are not possible because of incompatibility

# Supplementary Table 2: RNA-seq Mapping Tools.

| **Sequence Mapping Tool** | **Version** | **Mapping Strategy and Usage** | **Algorithmic Notes** |
| --- | --- | --- | --- |
| Bowtie[^1^](#_ENREF_1) | 0.12.8 | Un-spliced mapping to transcriptome or genome | Burrows-Wheeler transform and FM-index  (<http://bowtie-bio.sourceforge.net/>) |
| Bowtie2[^2^](#_ENREF_2) | 2.0.0-beta6 |  | Burrows-Wheeler transform, FM-index-assisted seed alignment, dynamic programming (<http://bowtie-bio.sourceforge.net/bowtie2>) |
| BWA[^3^](#_ENREF_3) | 0.6.1 |  | Burrows-Wheeler transform (<http://bio-bwa.sourceforge.net/>) |
| Novoalign[^4^](#_ENREF_4) | 2.08.02 |  | Commercial software, algorithm un-published (<http://www.novocraft.com/products/novoalign/>) |
| RUM[^5^](#_ENREF_5) |  |  | Uses Bowtie in a multi-phase mapping to transcriptome and genome (<https://github.com/itmat/rum/>) |
| WHAM[^6^](#_ENREF_6) | 0.1.4 |  | Hash-based indexing and bitwise operations for fast alignment (<http://research.cs.wisc.edu/wham/>) |
| GSNAP[^7^](#_ENREF_7) | 2012-07-03 | Spliced mapping to genome & un-spliced mapping to transcriptome or genome | Minimal sampling strategy, oligomer chaining for approximate alignment, sandwich dynamic programming (<http://research-pub.gene.com/gmap/>) |
| Magic[^8^](#_ENREF_8)^,^[^9^](#_ENREF_9) | 2013 |  | Strand-aware seed-and-extend alignment of read pairs, with at least 8 exact bases at the end of each aligned segment, allowing accurate analysis of introns, SNPs, indels and poly(A)s  (<ftp://ftp.ncbi.nlm.nih.gov/repository/acedb/Software/Magic/>) |
| MapSplice[^10^](#_ENREF_10) | 1.15.2 | Spliced mapping to genome | Uses Bowtie for alignment, segmented mapping (<http://www.netlab.uky.edu/p/bioinfo/MapSplice>) |
| OSA[^11^](#_ENREF_11) | 1.7.3 |  | Two-stage transcriptome and genome alignment, segmented mapping (<http://www.arrayserver.com/wiki/index.php?title=OSA>) |
| STAR[^12^](#_ENREF_12) | 2.3.0e |  | Sequential maximum mappable seed search, and seed clustering and stitching (<https://github.com/alexdobin/STAR>) |
| Subread[^13^](#_ENREF_13) | 1.3.2 |  | Segmented seed-and-vote mapping (<http://bioinf.wehi.edu.au/subread/>) |
| TopHat[^14^](#_ENREF_14) | 2.0.4 |  | Uses Bowtie or Bowtie2 for alignment, segmented mapping (<http://ccb.jhu.edu/software/tophat/>) |

BWA stands for Burrows-Wheeler aligner; RUM, RNA-seq unified mapper; WHAM, Wisconsin's high-throughput alignment method; GSNAP, genomic short-read nucleotide alignment program; OSA, Omicsoft sequence aligner; and STAR, spliced transcripts alignment to a reference.

# Supplementary Table 3: RNA-seq Gene Expression Quantification Tools.

| **Expression Quantification Tool** | **Version** | **Mathematical Model & Estimation Method** | **Quantification Targets** |
| --- | --- | --- | --- |
| HTSeq[^15^_ENREF_15](#_ENREF_15)  (<http://www-huber.embl.de/HTSeq/>) | 0.5.3p9 | Count-based; accumulated counts | Gene |
| Magic quantifier (for Magic mapper only)[^8^](#_ENREF_8)^,^[^9^](#_ENREF_9)  (<ftp://ftp.ncbi.nlm.nih.gov/repository/acedb/Software/Magic>) | 2013 |  | Gene, Isoform |
| featureCounts (for Subread mapper only)[^16^](#_ENREF_16)[_ENREF_13](#_ENREF_13)  (<http://bioinf.wehi.edu.au/featureCounts/>) | 1.3.2 |  | Gene, Isoform |
| RUM quantifier (for RUM mapper only)[^5^](#_ENREF_5)  (<https://github.com/itmat/rum/>) | 1.11 |  | Gene, Isoform |
| Cufflinks[^17^_ENREF_17](#_ENREF_17)  (<http://cole-trapnell-lab.github.io/cufflinks/>) | 2.0.2 | Poisson model; Maximum likelihood with maximum a posteriori estimates using Bayesian inference | Gene, Isoform |
| RSEM (RNA-Seq by Expectation-Maximization)[^18^](#_ENREF_18)  (<http://deweylab.github.io/RSEM/>) | 1.2.0 | Poisson model; Maximum likelihood estimation using expectation-maximization algorithm | Gene, Isoform |

# Supplementary Table 4: RNA-seq Gene Expression Normalization Methods.

| **Normalization Method** | **Description** |
| --- | --- |
| Reads/fragments per million mapped reads/fragments (RPM/FPM)[^19^](#_ENREF_19) | Scaling by library size |
| Reads/fragments per kilobase per million mapped reads/fragments (RPKM/FPKM)[^20^](#_ENREF_20)[_ENREF_17](#_ENREF_17) | Scaling by library size and gene/transcript length |
| Median[^21^](#_ENREF_21) | Scaling by median of all counts |
| Upper Quartile (UQ)[^19^](#_ENREF_19) | Scaling by upper quartile of all counts |
| Relative log expression (RLE)[^22^](#_ENREF_22) | Scaling by median ratio to median library |
| Trimmed mean of M-values (TMM)[^23^](#_ENREF_23) | Scaling by estimate of relative RNA production (<http://www.bioconductor.org/packages/release/bioc/html/edgeR.html>) |
| Magic expression index (EIndex)[^8^](#_ENREF_8)^,^[^9^](#_ENREF_9) | Magic pipeline only, scaling and thresholding to identify low-expression genes (<ftp://ftp.ncbi.nlm.nih.gov/repository/acedb/Software/Magic>) |

# Supplementary Table 5: SEQC Benchmark Datasets.

| **Platform** | **Data Acquisition Site(s)** | **Samples Acquired** | **Replicates per Sample** | **Notes** |
| --- | --- | --- | --- | --- |
| Illumina HiSeq 2000 | Beijing Genomics Institute (BGI) and Mayo Clinic (MAY) | A, B, C, D | 4 | Each sample replicate was sequenced in 16 lanes across two flow cells, but we used data from only two lanes of a single flow cell for this study. |
| Bio-Rad PrimePCR | FDA | A, B, C, D | 1 | The assay contains 20,801 genes. |

# Supplementary Table 6: SEQC Benchmark Samples.

| **Sample Name** | **Sample Description** |
| --- | --- |
| A | Stratagene’s Universal Human Reference RNA (UHRR) |
| B | Ambion’s Human Brain Reference RNA (HBRR) |
| C | A mixture of 75% A and 25% B |
| D | A mixture of 25% A and 75% B |

# Supplementary Table 7: RNA-seq Pipeline Metrics.

| **Metric** | **Description** | **Number of Raw Metric Values** | **Summary Statistics** |
| --- | --- | --- | --- |
| Accuracy | Accuracy is defined as the deviation of RNA-seq pipeline-derived log ratios from the corresponding qPCR-based log ratios. | 10,222 or 2,044 | Median |
| Precision | Precision is defined as the coefficient of variation over sample replicate libraries | 40,888 or 8,176  (10,222 or 2,044 genes × 4 samples) |  |
| Reliability | Reliability is defined as the intraclass (or intra-sample in our case) correlation that quantifies how similar replicate libraries of a sample are to one another using ANOVA techniques | 10,222 or 2,044 |  |

*All metrics are computed based on either 10,222 genes (denoted as “All Genes”) or 2,044 genes (denoted as “Low-Expressing Genes”)

# Supplementary Table 8: Benchmark Metric Performance for the 278 RNA-seq Pipelines.

(See Additional Files)

The table summarizes the performance of the 6 benchmark metrics (i.e., 1. Accuracy for all genes, 2. Accuracy for low-expression genes, 3. Precision for all genes, 4. Precision for low-expression genes, 5. Reliability for all genes, 6. Reliability for low-expression genes) for the 278 RNA-seq pipelines. RNA-seq pipelines were factored into “Mapping Algorithm,” “Mapping Strategy,” “Mapping Reporting,” “Quantification,” and “Normalization.”

# Supplementary Table 9: Prediction Endpoints for the SEQC Neuroblastoma Dataset.

| **Endpoint** | **Grouping Criteria** | **Number of Samples** |
| --- | --- | --- |
| Event-Free Survival (EFS)  [Threshold = 2 years] | Event occurred after the threshold  OR  Patient’s last follow-up exceeded the threshold  (no information about patient’s event occurrence after the last follow-up) | 67 |
|  | Event occurred before the threshold | 97 |
| Overall Survival  (OS)  [Threshold = 3 years] | Patient died after the threshold  OR  Patient’s last follow-up exceeded the threshold  (no information about patient’s survival after the last follow-up) | 83 |
|  | Patient died before the threshold | 70 |

# Supplementary Table 10: Prediction Endpoint for the TCGA Lung Adenocarcinoma Dataset.

| **Endpoint** | **Grouping Criteria** | **Number of Samples** |
| --- | --- | --- |
| Survival  [Threshold = 2 years] | Patient died after the threshold  OR  Patient’s last follow-up exceeded the threshold  (no information about patient’s survival after the last follow-up) | 47 |
|  | Patient died before the threshold | 40 |

# Supplementary Table 11: Predictive Modeling Performance for the SEQC Neuroblastoma Dataset.

(See Additional Files)

The table summarizes 1668 predictive modeling results (278 pipelines × 2 endpoints × 3 classifiers) for the 278 RNA-seq pipelines applied to the two SEQC neuroblastoma endpoints—event-free survival (Event) and overall survival (Death). The three classifiers are adaptive boosting (AdaBoost), logistic regression (LR), and support vector machines (SVM), and the two performance measures are the area under the curve (AUC) and the Matthews correlation coefficient (MCC). RNA-seq pipelines were factored into “Mapping Algorithm,” “Mapping Strategy,” “Mapping Reporting,” “Quantification,” and “Normalization.”

# Supplementary Table 12: Predictive Modeling Performance for the Lung Adenocarcinoma Dataset.

(See Additional Files)

The table summarizes 468 predictive modeling results (156 pipelines × 1 endpoint × 3 classifiers) for the 156 RNA-seq pipelines applied to the TCGA lung adenocarcinoma endpoint, survival (Death). The three classifiers are adaptive boosting (AdaBoost), logistic regression (LR), and support vector machines (SVM), and the two performance measures are the area under the curve (AUC) and the Matthews correlation coefficient (MCC). RNA-seq pipelines were factored into “Mapping Algorithm,” “Mapping Strategy,” “Mapping Reporting,” “Quantification,” and “Normalization.”

# Supplementary Table 13: Predictive Modeling Performance Measured by the Patient Stratification Success Rate (P-value Threshold=0.05).

(See Additional Files)

The table summarizes the success rates of patient stratification based on Kaplan-Meier survival analysis with the p-value threshold of 0.05 for the 278 and 156 RNA-seq pipelines applied to the neuroblastoma and lung adenocarcinoma endpoints, respectively. RNA-seq pipelines were factored into “Mapping Algorithm,” “Mapping Strategy,” “Mapping Reporting,” “Quantification,” and “Normalization.”

# Supplementary Table 14: Mapping Statistics of SEQC-Benchmark Dataset.

(See Additional Files)

# Supplementary Table 15: Comparison of the SEQC Pipeline Study to Previous Studies.

| **Pipeline Component** | **Ref.** | **Observations from Previous Studies** | **Top 20 Pipelines from the SEQC Pipeline Study**  **[Format—Mapping + Quantification + Normalization]** | |
| --- | --- | --- | --- | --- |
| Sequence Mapping | Grant  *et al.*[^5^](#_ENREF_5) | RUM, GSNAP (spliced), and MapSplice achieved the most accurate base-level alignment and junction detection | Accuracy   - **GSNAP** (**Spliced**, Single-Hit / Multi-Hit) + Count-Based / Cufflinks + Med. - **GSNAP** (Un-Spliced, Single-Hit / Multi-Hit) + RSEM + Med. - **RUM** + Count-Based + Med.   Precision   - **GSNAP** (Un-Spliced, Single-Hit / Multi-Hit) + Count-Based + UQ / FPM / FPKM - **GSNAP** (Un-Spliced, Single-Hit / Multi-Hit) + Cufflinks + UQ / RLE / TMM / FPM / FPKM   Reliability   - **GSNAP** (**Spliced**, Single-Hit / Multi-Hit) + Count-Based / Cufflinks + Med. - **GSNAP** (Un-Spliced / **Spliced**, Single-Hit / Multi-Hit) + Count-Based + UQ - **RUM** + Count-Based + Med. - **GSNAP** (Un-Spliced, Single-Hit / Multi-Hit) + Cufflinks + Med. / UQ - **GSNAP** (Un-Spliced, Single-Hit) + Count-Based + Med. - **GSNAP** (Un-Spliced, Single-Hit / Multi-Hit) + RSEM + Med.   Reproducibility   - **GSNAP** (Un-Spliced, Single-Hit / Multi-Hit) + Cufflinks + FPM / UQ / RLE / Med. / TMM | |
| Sequence Mapping | Lindner  *et al.*[^24^](#_ENREF_24) | Bowtie is a recommended tool for RNA-seq mapping.  Bowtie2 and BWA also show good performance in parameter stability and error tolerance | Accuracy   - **Bowtie2** (Single-Hit / Multi-Hit) + Count-based / Cufflinks / RSEM + Med. - **BWA** + Cufflinks / Count-Based / RSEM + Med. - **Bowtie** + RSEM + Med.   Precision   - **Bowtie2** (Multi-Hit) + Cufflinks + FPM / FPKM / UQ / RLE / TMM   Reliability   - **Bowtie2** (Single-Hit / Multi-Hit) + Count-based / Cufflinks / RSEM + Med. - **BWA** + Cufflinks / Count-Based / RSEM + Med. - **Bowtie** + RSEM + Med.   Reproducibility   - **Bowtie2** (Single-Hit / Multi-Hit) + Cufflinks + FPM / Med. / UQ / RLE / TMM - **BWA** + Cufflinks + FPM / Med. / UQ / RLE / TMM | |
| Sequence Mapping | Engström  *et al.*[^25^](#_ENREF_25) | GSNAP (spliced), MapSplice, and STAR compared favorably to the other methods in terms of read placement accuracy, indel alignment accuracy, and impact on transcript assembly | Accuracy   - **GSNAP** (**Spliced**, Single-Hit / Multi-Hit) + Count-Based / Cufflinks + Med. - **GSNAP** (**Spliced**, Single-Hit) + Cufflinks + FPM / FPKM - **STAR** + Cufflinks / Count-Based + Med. - **MapSplice** + Cufflinks / Count-Based + Med.   Reliability   - **GSNAP** (**Spliced**, Single-Hit / Multi-Hit) + Count-Based / Cufflinks + Med. - **STAR** + Cufflinks / Count-Based + Med. - **MapSplice** + Cufflinks / Count-Based + Med. | |
| Sequence Mapping | Hatem  *et al.*[^26^](#_ENREF_26) | “…no single tool outperforms all others in all metrics. Bowtie generally performed well, and BWA performed better for longer read lengths.” | No single pipeline or pipeline component can yield the best performance for all metrics.  Accuracy   - **BWA** + Count-Based / Cufflinks / RSEM + Med. - **Bowtie** + RSEM + Med.   Reliability   - **BWA** + Count-Based / Cufflinks / RSEM + Med. - **Bowtie** + RSEM + Med.   Reproducibility   - **BWA** + Cufflinks + FPM / Med. / UQ / RLE / TMM | |
| Sequence Mapping | Borozan  *et al.*[^27^](#_ENREF_27) | “When information about splice junctions was not available, our results showed that GSNAP has the best performance and scores highest according to the FS measure when aligning RNA-Seq human reads back to the human reference and across splice junctions, …” | Accuracy   - **GSNAP** (**Spliced**, Single-Hit / Multi-Hit) + Count-Based + Med. - **GSNAP** (**Spliced**, Single-Hit) + Cufflinks + FPM / FPKM + Med. - **GSNAP** (Un-Spliced, Single-Hit / Multi-Hit) + RSEM + Med.   Precision   - **GSNAP** (Un-Spliced, Single-Hit / Multi-Hit) + Count-Based / Cufflinks + UQ / FPM / FPKM / RLE / TMM   Reliability   - **GSNAP** (Un-Spliced, Multi-Hit) + Cufflinks + UQ - **GSNAP** (Un-Spliced / **Spliced**, Single-Hit / Multi-Hit) + Cufflinks + Med. - **GSNAP** (Un-Spliced / **Spliced**, Single-Hit / Multi-Hit) + Count-Based + UQ / Med. - **GSNAP** (Un-Spliced, Single-Hit / Multi-Hit) + RSEM + Med.   Reproducibility   - **GSNAP** (Un-Spliced, Single-hit / Multi-Hit) + Cufflinks + FPM / UQ / RLE / Med. / TMM | |
| Expression Quantification | Li  *et al.*[^18^](#_ENREF_18) | RSEM resulted in the most accurate expression estimates. | Multiple sequence mapping tools combined with RSEM lead to high accuracy and reliability.  Accuracy   - Bowtie2 (Single-Hit / Multi-Hit) / BWA / Bowtie / WHAM (Single-Hit / Multi-Hit) / GSANP (Un-Spliced, Single-Hit / Multi-Hit) / Novoalign (Single-Hit / Multi-Hit) + **RSEM** + Med.   Reliability   - Bowtie2 (Single-Hit / Multi-Hit) / BWA / Bowtie / WHAM (Single-Hit / Multi-Hit) / GSANP (Un-Spliced, Single-Hit / Multi-Hit) + **RSEM** + Med. | |
| Expression Quantification | Kanitz  *et al.*[^28^](#_ENREF_28) | RSEM is among the best performers in terms of accuracy measured by Spearman’s rank correlation coefficient using both simulated and replicate data. | Multiple sequence mapping tools combined with RSEM lead to high accuracy and reliability.  Accuracy   - Bowtie2 (Single-Hit / Multi-Hit) / BWA / Bowtie / WHAM (Single-Hit / Multi-Hit) / GSANP (Un-Spliced, Single-Hit / Multi-Hit) / Novoalign (Single-Hit / Multi-Hit) + **RSEM** + Med.   Reliability  Bowtie2 (Single-Hit / Multi-Hit) / BWA / Bowtie / WHAM (Single-Hit / Multi-Hit) / GSANP (Un-Spliced, Single-Hit / Multi-Hit) + **RSEM** + Med. | |
| Expression Quantification | Bray  *et al.*[^29^](#_ENREF_29) | RSEM performed the best in terms of accuracy measured by median relative difference. | Multiple sequence mapping tools combined with RSEM lead to high accuracy and reliability.  Accuracy   - Bowtie2 (Single-Hit / Multi-Hit) / BWA / Bowtie / WHAM (Single-Hit / Multi-Hit) / GSANP (Un-Spliced, Single-Hit / Multi-Hit) / Novoalign (Single-Hit / Multi-Hit) + **RSEM** + Med.   Reliability  Bowtie2 (Single-Hit / Multi-Hit) / BWA / Bowtie / WHAM (Single-Hit / Multi-Hit) / GSANP (Un-Spliced, Single-Hit / Multi-Hit) + **RSEM** + Med. | |
|  | Teng *el al.*[^30^](#_ENREF_30) | RSEM slightly outperforms the other quantification methods compared. | Multiple sequence mapping tools combined with RSEM lead to high accuracy and reliability.  Accuracy   - Bowtie2 (Single-Hit / Multi-Hit) / BWA / Bowtie / WHAM (Single-Hit / Multi-Hit) / GSANP (Un-Spliced, Single-Hit / Multi-Hit) / Novoalign (Single-Hit / Multi-Hit) + **RSEM** + Med.   Reliability  Bowtie2 (Single-Hit / Multi-Hit) / BWA / Bowtie / WHAM (Single-Hit / Multi-Hit) / GSANP (Un-Spliced, Single-Hit / Multi-Hit) + **RSEM** + Med. | |
| Expression Normalization | Dillies  *et al.*[^21^](#_ENREF_21) | TMM and DESeq (i.e., RLE) normalization resulted in the lowest coefficient of variation | Precision   - - - GSNAP (Un-Spliced, Single-Hit / Multi-Hit) + Count-Based / Cufflinks + **RLE** / **TMM**     - Subread + Count-Based + **RLE** / **TMM**     - Bowtie2 (Multi-Hit) + Cufflinks + **RLE** / **TMM**   Reproducibility   - - - GSNAP (Un-Spliced, Single-Hit / Multi-Hit) + Cufflinks + **RLE** / **TMM**     - Subread + Count-Based + **RLE** / **TMM**     - Bowtie2 (Single-Hit / Multi-Hit) + Cufflinks + **RLE** / **TMM**     - BWA + Cufflinks + **RLE** / **TMM**     - TopHat (Single-Hit / Multi-Hit) + Cufflinks + **RLE** / **TMM** | |
| Expression Normalization | Maza  *et al.*[^31^](#_ENREF_31) | Total count (i.e., FPM) resulted in the best DEG detection performance. | Accuracy   - - - GSNAP (Spliced, Single-Hit) + Cufflinks + **FPM**   Precision   - - - Bowtie2 (Multi-Hit) + Cufflinks + **FPM**     - GSNAP (Un-Spliced, Single-Hit / Multi-Hit) + Count-Based / Cufflinks + **FPM**   Reproducibility   - - - Bowtie2 (Single-Hit / Multi-Hit) / GSNAP (Un-Spliced, Single-Hit / Multi-Hit) + Cufflinks + **FPM**     - Subread + Count-Based + **FPM**     - BWA + Cufflinks + **FPM**     - TopHat (Single-Hit / Multi-Hit) + Cufflinks + **FPM** | |
| Expression Normalization | Aanes  *et al.*[^32^](#_ENREF_32) | Total count (i.e., FPM) normalization outperformed TMM normalization in terms of fold-change estimates | Accuracy   - - - GSNAP (Spliced, Single-Hit) + Cufflinks + **FPM**   Precision   - - - Bowtie2 (Multi-Hit) + Cufflinks + **FPM**     - GSNAP (Un-Spliced, Single-Hit / Multi-Hit) + Count-Based / Cufflinks + **FPM**   Reproducibility   - - - Bowtie2 (Single-Hit / Multi-Hit) / GSNAP (Un-Spliced, Single-Hit / Multi-Hit) + Cufflinks + **FPM**     - Subread + Count-Based + **FPM**     - BWA + Cufflinks + **FPM**     - TopHat (Single-Hit / Multi-Hit) + Cufflinks + **FPM** | |
| **Full Pipeline** | **Ref.** | **Observations from Previous Studies** | | **Observations from the SEQC Pipeline Study** |
| Pipeline Comparison | Fonseca  *et al.*[^33^](#_ENREF_33) | Quantification methods have larger impact on the results than alignment methods. Any of OSA, STAR, or TopHat mapping with HTSeq quantification resulted in the most accurate expression estimates. | | Normalization is the dominant factor for all the four metrics, while the precision metric also significantly depends on mapping, quantification algorithms and their interaction.  Any of OSA, STAR, or TopHat mapping with HTSeq quantification is a good pipeline but not the best-performing pipelines in our study. |
| Pipeline Comparison | Nookaew  *et al.*[^34^](#_ENREF_34) | “…accurate mapping is of fundamental importance to estimate gene expression level…” | | While accurate mapping is important, we found that several other RNA-seq pipeline components are also important factors in estimating gene expression level. |

# Supplementary Note 1: Sequence Mapping and Expression Quantification Protocols.

***Sequence Mapping***

1. **Reference Sequence Preparation**

The mapping stage of the RNA-seq pipelines was divided into three steps that mapped the RNA-seq reads to three different references. The first reference included External RNA Controls Consortium (ERCC) sequences, the mitochondrial genome (the NCBI reference—NC_012920.1), and ribosomal RNA (the NCBI references—NR_003287.2, NR_003286.2, NR_003285.2, and NR_023363.1) sequences. We combined these sequences into a single multi-FASTA file. The second reference was the AceView transcriptome. The AceView annotation (i.e., the GTF file) was exported from the AceView repository on November 14th, 2011, and it included only transcripts from the 2010 release constructed from GenBank mRNAs and ESTs (expressed sequence tag). We removed single-exon genes and all non-exon elements from the GTF file, and then used the GTF file to extract all transcript sequences from the human genome using a TopHat utility called gtf_to_fasta. The third reference was the human genome, which was also used to construct the transcriptome reference in the previous step. We used the hg19 reference downloaded from UCSC on November 12th, 2011 and prepared the sequences by removing the mitochondrial genome (chrM) and the haplotype variants. Thus, the final human genome reference included only the main chromosomes, un-localized contigs (sequences with unknown chromosome), and un-placed contigs (sequences with known chromosome but unknown chromosomal location).

1. **Bowtie (with RSEM quantification)**

Index Preparation

rsem-prepare-reference --gtf [GTF File] [Genome Fasta] [Output Prefix]

Mapping and Quantification

rsem-calculate-expression --paired-end -p [CPUs] --bowtie-path [Path to Bowtie] --phred33/64-quals --no-bam-output Read_1.fastq Read_2.fastq [RSEM Index] [Output Prefix]

1. **Bowtie2**

Index Preparation

bowtie2-build [Reference Fasta File] [Reference Index Output]

Single-Hit Mapping

bowtie2 -p [CPUs] -x [Reference Index (either AceView or HG)] -1 [read1.fastq] -2 [read2.fastq] > [output file.sam]

Multi-Hit Mapping

bowtie2 -p [CPUs] -k 200 -x [Reference Index (either AceView or HG)] -1 [read1.fastq] -2 [read2.fastq] > [output file.sam]

1. **BWA**

Index Preparation

bwa_index [Reference Fasta File]

Mapping

bwa aln -1 -t [CPUs] [Reference Index (either AceView or HG)] read1.fastq > output1.sai

bwa aln -2 -t [CPUs] [Reference Index (either AceView or HG)] read2.fastq > output2.sai

bwa sampe [Reference Index (either AceView or HG)] output1.sai output2.sai read1.fastq read2.fastq > output.sam

1. **GSNAP**

Index Preparation

gmap_build -D [Reference Index Output Directory] -d [Reference Index Output Name] [Reference Fasta File]

Spliced Index Preparation (for Spliced Mapping)

iit_store -o transcriptome.iit [Reference Transcriptome GTF]

Single-Hit Un-Spliced Mapping

gsnap --nthreads [CPUs] -B 5 -D [Reference Index Directory] --format=sam -n 1 -J 33 -d [Reference Index Name] read1.fastq read2.fastq > output.sam

Multi-Hit Un-Spliced Mapping

gsnap --nthreads [CPUs] -B 5 -D [Reference Index Directory] --format=sam -n 200 -J 33 -d [Reference Index Name] read1.fastq read2.fastq > output.sam

Single-Hit Spliced Mapping

gsnap --nthreads [CPUs] -B 5 -D [Reference Index Directory] --format=sam -n 1 -J 33 --d [HG Reference Index Name] -s [Transcriptome Spliced Index (*.iit)] --nofails read1.fastq read2.fastq > output.sam

Multi-Hit Spliced Mapping

gsnap --nthreads [CPUs] -B 5 -D [Reference Index Directory] --format=sam -n 200 -J 33 --d [HG Reference Index Name] -s [Transcriptome Spliced Index (*.iit)] --nofails read1.fastq read2.fastq > output.sam

1. **MapSplice**

Index Preparation

Same as Bowtie

Mapping (SEQC-benchmark Data)

python mapsplice_segments.py -u Read_1.fastq,Read_2.fastq -B [Bowtie Genome Index] -c [Per Contig Genome FASTA Files] -o [Output Directory] [Configuration File]

*Configuration File:*

reads_format = FASTQ

segment_mismatches = 1

segment_length = 25

read_length = 100

paired_end = yes

junction_type = non-canonical

full_running = yes

anchor_length = 8

remove_temp_files = yes

remap_mismatches = 2

splice_mismatches = 0

min_intron_length = 70

max_intron_length = 500000

threads = [CPUs]

search_whole_chromosome = no

map_segment_directly = no

run_MapPER = no

do_fusion = no

do_cluster = no

Mapping (SEQC-application, Neuroblastoma data)

python mapsplice -p [CPUs] -o [Output Directory] --min-intron 70 --max-intron 500000 --bam -c [Per Contig Genome FASTA Files] -x [Bowtie Genome Index] -1 Read_1.fastq -2 Read_2.fastq

1. **Novoalign**

Index Preparation

novoindex -t [CPUs] [Reference Output Index (*.ndx)] [Reference Fasta File]

Single-Hit Mapping

novoalign -c [CPUs] -F ILMFQ -o SAM -r Random -d [Reference Index] -f read1.fastq read2.fastq > output.sam

Multi-Hit Mapping

novoalign -c [CPUs] -F ILMFQ -o SAM -r all -e 200 -d [Reference Index] -f read1.fastq read2.fastq > output.sam

1. **OSA**

Index Preparation

osa.exe --buildref [Base_Dir] [genome_fasta_file_name] [ref_lib_prefix]

osa.exe --buildgm [Base_Dir] [gtf_file_name] [ref_lib_prefix] [gene_model_prefix]

Mapping

mono osa.exe --alignrna [OSA Index Directory] [Reference Genome Prefix] [Genome Annotation Prefix] [Configuration File]

*Configuration File:*

<Files>

Read_1.fastq.gz

Read_2.fastq.gz

<Options>

ThreadNumber=CPUs

PairedEnd=True

FileFormat=FASTQ

AutoPenalty=True

FixedPenalty=2

Gzip=True

ExpressionMeasurement=TPM

SearchNovelExonJunction=False

GenerateSamFiles=False

WriteReadsInSeparateFiles=False

InsertSizeStandardDeviation=70

ExpectedInsertSize=170

ExcludeUnmappedInBam=True

<Output>

OutputName=OSA_ILM

OutputPath=XYZ

1. **RUM**

Index Preparation

perl create_indexes_from_ucsc.pl NAME_genome.txt NAME_refseq_ucsc

Mapping

rum_runner align -i [RUM Genome Index] -o [Output Directory] --name [Sample Name] --chunks [CPUs] Read_1.fastq Read_2.fastq

1. **STAR**

Index Preparation

STAR --runThreadN 24 --runMode genomeGenerate --genomeDir aceview_seqc --genomeFastaFiles [HG Reference Fasta] --sjdbGTFfile [Transcriptome Reference GTF] --sjdbOverhang 100

Mapping

STAR --genomeDir [STAR Genome Index] --readFilesIn [Read_1.fastq.gz] [Read_2.fastq.gz] --runThreadN [CPUs] --readFilesCommand zcat --outFileNamePrefix [Output Prefix] --outFilterMultimapNmax 200 --outSAMstrandField intronMotif --outFilterIntronMotifs RemoveNoncanonical --genomeLoad LoadAndKeep

1. **Subread**

Index Preparation

subread-buildindex -B [HG Reference Index Name] [HG Reference Fasta]

Mapping

subread-align -r read1.fastq -R read2.fastq -T [CPUs] -P 6 -u -H -i [HG Reference Index] -o output.sam

featureCounts Quantification

featureCounts -p -T [CPUs] -t exon -g gene_id -a [Transcriptome GTF] -o output.counts input.sam

1. **TopHat**

Index Preparation

Same as Bowtie2

Single-Hit Mapping

tophat -p [CPUs] -o [Output Directory] --max-multihits 1 --library-type fr-unstranded --solexa1.3-quals --no-novel-juncs -G [GTF File] --transcriptome-index [Transcriptome Reference Index] [HG Reference Index] [Read_1.fastq.gz] [Read_2.fastq.gz]

Multi-Hit Mapping

tophat -p [CPUs] -o [Output Directory] --max-multihits 200 --library-type fr-unstranded --solexa1.3-quals --no-novel-juncs -G [GTF File] --transcriptome-index [Transcriptome Reference Index] [HG Reference Index] [Read_1.fastq.gz] [Read_2.fastq.gz]

1. **WHAM**

Index Preparation

wham-build -l 90 [Reference Fasta File] [Reference Index Output]

Single-Hit Mapping

wham -l 90 -g 1 -X 500 --best -S -t [CPUs] -k 1 -1 read1.fastq -2 read2.fastq [Reference Index] output.sam

Multi-Hit Mapping

wham -l 90 -g 1 -X 500 --best -S -t [CPUs] -k 200 -1 read1.fastq -2 read2.fastq [Reference Index] output.sam

***Expression Quantification***

1. **HTSeq**

samtools view -h [BAM File] | htseq-count --mode=intersection-nonempty --stranded=no --type=exon --idattr=gene_id - [GTF File] > [Output File]

1. **Cufflinks**

cuffdiff -o [Output Directory] -p [CPUs] -b [Reference Genome FASTA] -u -L [Sample Labels] [GTF Files] [BAM Files]

1. **RSEM**

rsem-calculate-expression --bam --paired-end -p [CPUs] --no-bam-output [BAM File] [RSEM Index] [Output Prefix]

**Supplementary Note 2: Reproducibility Measured as Inter-replicate Correlation of Gene Expression.**

According to the definition described in Bartlett’s article published in 2008, reproducibility refers to “the variation in measurements made on a subject under changing conditions[^35^](#_ENREF_35).” The changing conditions may involve with different instruments for measurements, different measurement time points, different observers, and many other factors. In the context of our study, since no two replicate libraries of RNA-seq are exactly identical to each other due to various biological, chemical, instrumental, and experimental factors, the assessment of variation between replicate libraries falls within the scope of reproducibility. We chose to use the Spearman correlation coefficient, a commonly applied metric, as the measure of ***reproducibility***. Higher Spearman correlation coefficients indicate higher reproducibility.

We computed the pairwise Spearman correlation coefficient between replicate libraries for the same sample *s*, where $s\in\left\{ A,B,C,D \right\}$. Since each sample has four replicate libraries, six and 24 pairwise comparisons are available for each sample the entire dataset, respectively. We then used the median of all 24 Spearman correlation coefficients as the final measure of reproducibility.

We defined the ***reproducibility*** metric as the Spearman correlation between two replicate libraries of the same sample, and visualized the median reproducibility of AllGenes and LowExpressGenes using heatmaps (**Supplementary Figure 8** and **Supplementary Figure 10**). We observed the following results:

1. Using AllGenes, the Spearman correlation ranged from 0.993 to 0.996 (**Supplementary Figure 8**). Larger Spearman correlation represents higher reproducibility. Since the Spearman correlation is based on the rank values of gene expression, normalization methods scaling all expression with the same factor (i.e., all but the FPKM method) will lead to the same reproducibility values. The FPKM normalization method always exhibited the lower Spearman correlation, or the lower reproducibility, than the other normalization methods. In addition, pipelines with either Novoalign or GSNAP un-spliced mapping and RSEM quantification resulted in lower Spearman correlation despite the choice of normalization methods. Normalization, two-way [mapping algorithm*quantification] interaction, quantification, and mapping algorithm were the largest statistically significant (p<0.05) source of variation (**Supplementary Figure 9**).
2. The Spearman correlation using LowExpressGenes was smaller than that using AllGenes, and it ranged from 0.962 to 0.987 (**Supplementary Figure 10**). The trends of pipeline performance were similar to those using all genes. Normalization, quantification, two-way [mapping algorithm*quantification] interaction, and mapping algorithm were the largest statistically significant (p<0.05) source of variation (**Supplementary Figure 11**).

In summary, all but the FPKM normalization method with either Subread mapping and count-based quantification or GSNAP un-spliced mapping and Cufflinks quantification were the best choice for quantifying genes with high reproducibility, or high Spearman correlation.

**References**

1 Langmead, B., Trapnell, C., Pop, M. & Salzberg, S. L. Ultrafast and memory-efficient alignment of short DNA sequences to the human genome. *Genome Biol* **10**, R25 (2009).

2 Langmead, B. & Salzberg, S. L. Fast gapped-read alignment with Bowtie 2. *Nature methods* **9**, 357-359 (2012).

3 Li, H. & Durbin, R. Fast and accurate short read alignment with Burrows–Wheeler transform. *Bioinformatics* **25**, 1754-1760 (2009).

4 Novocraft. *NovoAlign*, <<http://www.novocraft.com/products/novoalign/>> (

5 Grant, G. R. *et al.* Comparative analysis of RNA-Seq alignment algorithms and the RNA-Seq unified mapper (RUM). *Bioinformatics* **27**, 2518-2528 (2011).

6 Li, Y., Terrell, A. & Patel, J. M. in *SIGMOD Conference.* 445-456.

7 Wu, T. D. & Nacu, S. Fast and SNP-tolerant detection of complex variants and splicing in short reads. *Bioinformatics* **26**, 873-881 (2010).

8 Thierry-Mieg, D. & Thierry-Mieg, J. AceView: a comprehensive cDNA-supported gene and transcripts annotation. *Genome Biology* **7**, doi:Doi 10.1186/Gb-2006-7-S1-S12 (2006).

9 Thierry-Mieg, D. & Thierry-Mieg, J. *Magic Analysis Tool*, <<ftp://ftp.ncbi.nlm.nih.gov/repository/acedb/Software/Magic/>> (

10 Wang, K. *et al.* MapSplice: accurate mapping of RNA-seq reads for splice junction discovery. *Nucleic acids research* **38**, e178-e178 (2010).

11 Hu, J., Ge, H. Y., Newman, M. & Liu, K. J. OSA: a fast and accurate alignment tool for RNA-Seq. *Bioinformatics* **28**, 1933-1934, doi:10.1093/bioinformatics/bts294 (2012).

12 Dobin, A. *et al.* STAR: ultrafast universal RNA-seq aligner. *Bioinformatics* **29**, 15-21 (2013).

13 Liao, Y., Smyth, G. K. & Shi, W. The Subread aligner: fast, accurate and scalable read mapping by seed-and-vote. *Nucleic acids research* (2013).

14 Trapnell, C., Pachter, L. & Salzberg, S. L. TopHat: discovering splice junctions with RNA-Seq. *Bioinformatics* **25**, 1105-1111 (2009).

15 Anders, S., Pyl, P. T. & Huber, W. HTSeq-a Python framework to work with high-throughput sequencing data. *Bioinformatics* **31**, 166-169, doi:10.1093/bioinformatics/btu638 (2015).

16 Liao, Y., Smyth, G. K. & Shi, W. featureCounts: an efficient general purpose program for assigning sequence reads to genomic features. *Bioinformatics* **30**, 923-930, doi:10.1093/bioinformatics/btt656 (2014).

17 Trapnell, C. *et al.* Transcript assembly and quantification by RNA-Seq reveals unannotated transcripts and isoform switching during cell differentiation. *Nat Biotechnol* **28**, 511-U174, doi:10.1038/nbt.1621 (2010).

18 Li, B. & Dewey, C. N. RSEM: accurate transcript quantification from RNA-Seq data with or without a reference genome. *BMC bioinformatics* **12**, 323 (2011).

19 Bullard, J. H., Purdom, E., Hansen, K. D. & Dudoit, S. Evaluation of statistical methods for normalization and differential expression in mRNA-Seq experiments. *Bmc Bioinformatics* **11**, doi:10.1186/1471-2105-11-94 (2010).

20 Mortazavi, A., Williams, B. A., Mccue, K., Schaeffer, L. & Wold, B. Mapping and quantifying mammalian transcriptomes by RNA-Seq. *Nature Methods* **5**, 621-628, doi:10.1038/nmeth.1226 (2008).

21 Dillies, M. A. *et al.* A comprehensive evaluation of normalization methods for Illumina high-throughput RNA sequencing data analysis. *Briefings in bioinformatics*, doi:10.1093/bib/bbs046 (2012).

22 Anders, S. & Huber, W. Differential expression analysis for sequence count data. *Genome Biol* **11**, R106 (2010).

23 Robinson, M. D. & Oshlack, A. A scaling normalization method for differential expression analysis of RNA-seq data. *Genome Biology* **11**, doi:10.1186/Gb-2010-11-3-R25 (2010).

24 Lindner, R. & Friedel, C. C. A Comprehensive Evaluation of Alignment Algorithms in the Context of RNA-Seq. *PloS one* **7**, e52403, doi:10.1371/journal.pone.0052403 (2012).

25 Engström, P. G. *et al.* Systematic evaluation of spliced alignment programs for RNA-seq data. *Nature methods* **10**, 1185-1191 (2013).

26 Hatem, A., Bozdağ, D., Toland, A. E. & Çatalyürek, Ü. V. Benchmarking short sequence mapping tools. *BMC bioinformatics* **14**, 184 (2013).

27 Borozan, I., Watt, S. N. & Ferretti, V. Evaluation of alignment algorithms for discovery and identification of pathogens using RNA-Seq. *PloS one* **8**, e76935 (2013).

28 Kanitz, A. *et al.* Comparative assessment of methods for the computational inference of transcript isoform abundance from RNA-seq data. *Genome biology* **16**, 1-26 (2015).

29 Bray, N. L., Pimentel, H., Melsted, P. & Pachter, L. Near-optimal probabilistic RNA-seq quantification. *Nat Biotechnol* **34**, 525-527, doi:10.1038/nbt.3519 (2016).

30 Teng, M. *et al.* A benchmark for RNA-seq quantification pipelines. *Genome Biol* **17**, 74, doi:10.1186/s13059-016-0940-1 (2016).

31 Maza, E., Frasse, P., Senin, P., Bouzayen, M. & Zouine, M. Comparison of normalization methods for differential gene expression analysis in RNA-Seq experiments: A matter of relative size of studied transcriptomes. *Communicative & Integrative Biology* **6** (2013).

32 Aanes, H. *et al.* Normalization of RNA-sequencing data from samples with varying mRNA levels. *PloS one* **9**, e89158 (2014).

33 Fonseca, N. A., Marioni, J. & Brazma, A. RNA-seq gene profiling-a systematic empirical comparison. *PloS one* **9**, e107026 (2014).

34 Nookaew, I. *et al.* A comprehensive comparison of RNA-Seq-based transcriptome analysis from reads to differential gene expression and cross-comparison with microarrays: a case study in Saccharomyces cerevisiae. *Nucleic acids research*, gks804 (2012).

35 Bartlett, J. W. & Frost, C. Reliability, repeatability and reproducibility: analysis of measurement errors in continuous variables. *Ultrasound in obstetrics & gynecology : the official journal of the International Society of Ultrasound in Obstetrics and Gynecology* **31**, 466-475, doi:10.1002/uog.5256 (2008).

# Author List for SEQC Consortium

Wendell D. Jones^8^, Leming Shi^5,9^, Matthias Fischer^10^, Christopher E. Mason^11,12^, Sheng Li^11,12^, Joshua Xu^5^, Wei Shi^13,14^, Jian Wang^15^, Jean Thierry-Mieg^16^, Danielle Thierry-Mieg^16^, Falk Hertwig^10^, Frank Berthold^10^, Barbara Hero^10^, Yang Liao^13,14^, Gordon K. Smyth^13,17^, David Kreil^18,19^, Paweł P. Łabaj^18^, Dalila Megherbi^20^, Gary Schroth^21^, Hong Fang^5^

^8^Genomic Laboratories, Q^2^ Solutions - EA Genomics, Morrisville, North Carolina, USA

^9^School of Pharmacy and School of Life Sciences, Fudan University, Shanghai, China

^10^Department of Pediatric Oncology and Hematology and Center of Molecular Medicine Cologne, University of Cologne, Cologne, Germany

^11^Department of Physiology and Biophysics, Weill Cornell Medical College, New York, New York, USA

^12^The HRH Prince Alwaleed Bin Talal Bin Abdulaziz Alsaud Institute for Computational Biomedicine, Weill Cornell Medical College, New York, New York, USA

^13^Bioinformatics Division, The Walter and Eliza Hall Institute of Medical Research, Parkville, Victoria, Australia

^14^Department of Computing and Information Systems, The University of Melbourne, Parkville, Victoria, Australia

^15^Research Informatics, Lilly Corporate Center, Eli Lilly and Company, Indianapolis, Indiana, USA

^16^National Center for Biotechnology Information, National Library of Medicine, National Institutes of Health, Bethesda, Maryland, USA

^17^School of Mathematics and Statistics, The University of Melbourne, Parkville, Victoria, Australia

^18^Chair of Bioinformatics Research Group, Boku University Vienna, Vienna, Austria

^19^School of Life Sciences, University of Warwick, Coventry, UK

^20^CMINDS Research Center, Department of Electrical and Computer Engineering, University of Massachusetts, Lowell, Massachusetts, USA

^21^Illumina Inc., Hayward, California, USA
